# Chiral Salen-Based Organic Salts: Synthesis and Potential Antibacterial Activity

**DOI:** 10.3390/molecules30102173

**Published:** 2025-05-15

**Authors:** Marcin Gano, Michał Wójcicki, Ewa Janus

**Affiliations:** 1 Department of Organic Chemical Technology and Polymer Materials, Faculty of Chemical Technology and Engineering, West Pomeranian University of Technology in Szczecin, Pułaskiego 10 St., 70-322 Szczecin, Poland; marcin.gano@zut.edu.pl; 2 Center for Advanced Materials and Manufacturing Process Engineering (CAMMPE), West Pomeranian University of Technology in Szczecin, 70-310 Szczecin, Poland; 3 Department of Microbiology, Prof. Wacław Dąbrowski Institute of Agricultural and Food Biotechnology—State Research Institute, Rakowiecka 36 St., 02-532 Warsaw, Poland; michal.wojcicki@ibprs.pl

**Keywords:** chemical synthesis, chloride anion, tetrafluoroborate anion, trifluoromethanesulfonate anion, bis(trifluoromethylsulfonyl)imide anion, minimum inhibitory concentration, minimum bactericidal concentration, growth kinetics analysis, virulent bacteriophages, synergistic effect

## Abstract

New chiral salen-based organic salts were synthesised and evaluated for their antibacterial activity against *Serratia fonticola*, *Escherichia coli*, and *Enterobacter cloacae*. Their structures and physicochemical properties, namely their specific rotation, melting point, thermal stability, and antibacterial efficacy, including minimum inhibitory concentration (MIC) and minimum bactericidal concentration (MBC), were determined. The synergy between chiral organic salts and bacteriophages was also demonstrated. [(RR)Sal.5C1.PhIM][Cl], [(RR)Sal.5C1.PhIM][BF_4_], and [(RR)Sal.5C1.Pyr][OTf] had the lowest MIC values (from 500 mg mL^−1^ for *S. fonticola* strain KKP 3685 to 2000 mg mL^−1^ for *E. cloacae* strain KKP 3692), while the highest MICs (>4000 mg mL^−1^) were observed for [(RR)Sal.5C1.Pyr][OTf] and [(RR)Sal.5C1.PhIM][NTf_2_] against *E. cloacae* strain KKP 3692. The impact of the tested compounds on phage activity was strain-specific. A synergistic effect of [(RR)Sal.5C1.PhIM][BF_4_] at 0.5 mg mL^−1^ in microcultures with Escherichia phage KKP 3710 (at MOI of 10 and 100) on the complete inhibition of the growth of *E. coli* strain KKP 3688 was observed. The combination of [(RR)Sal.5C1.PhIM])][OTf] at 1 mg mL^−1^ with the addition of phages (at each MOI) and at 0.5 mg mL^−1^ and MOI = 100 completely inhibited the growth of *E. coli* strain KKP 3688. Moreover, [(RR)Sal.5C1.PhIM])][OTf] at 1 mg mL^−1^ and 0.5 mg mL^−1^, when combined with Enterobacter phage KKP 3716, inhibited the growth of *E. cloacae* strain KKP 3692 slightly more effectively than the compound alone at the same concentrations. These results suggest that combining our antibacterial agents can reduce chemical compound concentrations, with effects depending on the bacteria.

## 1. Introduction

Antibiotic resistance has become a serious problem in modern medicine. The increase in the number of microorganisms resistant to traditional treatment methods has led to a growing interest among scientists in searching for new forms of therapy. According to WHO (World Health Organisation) data, humanity has entered the post-antibiotic era, in which many deadly bacteria are resistant to currently used antibiotics [1]. A significant problem is antibiotic resistance transmission among bacterial strains within the food microbiota, highlighting the urgent need for alternative food preservation methods to inhibit bacterial growth and/or development in food [2,3,4,5]. Previous research conducted by Wójcicki et al. (2023) revealed that 18.6% of saprophytic bacteria isolated from minimally processed plant-based food products were multidrug-resistant (MDR) [6]. The biotechnology industry is currently focused on developing and commercialising biopreparations containing specific lytic bacteriophages to eliminate pathogenic bacteria in food [7,8,9]. However, when it comes to saprophytic bacteria, whose growth and development in food products lead to quality degradation and shortened shelf life [10,11], creating effective bacteriophage treatments remains a significant challenge. It is important to note that saprophytic bacteria in the food matrix can also serve as vectors for transmitting antibiotic resistance genes. These bacteria may contribute to the spread of antibiotic resistance within the food chain, threatening food safety and consumer health [12,13]. There is an intensive search for new compounds with antimicrobial activity that can replace currently used antibiotics, which many bacteria have become resistant to. Interesting compounds with antibacterial properties include ionic liquids, especially those containing imidazolium-, pyridinium-, pyrrolidinium-, phosphonium-, and ammonium cations in their structure in combination with different anions [14,15,16,17,18,19,20,21,22]. In some studies, antibiotic-based ionic liquids composed of either imidazolium or pyridinium cations showed improved antimicrobial activity on various bacterial strains compared to an antibiotic in sodium salt form [23]. The special antimicrobial properties of ionic liquids depend on the substituents in the cation. Moreover, the importance of the anion on the capability of ionic liquids to kill bacteria was indicated [24]. It has been shown that the combination of [NTf_2_]^−^ anion with pyrrolidinium cation containing butyl and octyl chains has the highest efficiency against *E. coli* and *Staphylococcus aureus*. In contrast, changing anion to [Br]^−^ showed the highest efficacy against these bacteria with the longer decyl and dodecyl substituents in the cation [25].

Salen-type Schiff bases are a class of chemical compounds with tremendous potential for numerous applications in pharmacy and organic synthesis. One of the most important features of salen-type Schiff bases is their ability to form complexes with metals. Metal complexes with salen-type ligands have been successfully applied in asymmetric synthesis and catalysis, e.g., in the Diels–Alder reaction [26,27] and epoxidation [28,29]. Their ability to form complexes has been explored in medicinal science for various applications, such as antibiotics, anticancer agents, and antiallergic drugs [30]. Recently, it has been shown that the incorporation of an ionic group, e.g., an ammonium, imidazolium, pyridinium, or morpholinium cation into the salen structure, led to enhanced activity and enantioselectivity compared to those obtained with a ligand without a positively charged substituent. The counterion also played an important role in this improvement. [31,32,33]. Moreover, imidazolium salts with an *N*-methylimidazolium core attached to *N*,*N*′-bis(salicylidene)-(±)*trans*-1,2-diaminocyclohexane, and Fe(II) complexed with a cation-functionalised salen ligand, revealing remarkable extra-potent activity against *Staphylococcus aureus* and *Bacillus subtilis* [34].

The aim of the present studies is the synthesis of chiral salen-based organic salts with various cationic fragments in the structure of salicylaldehyde combined with various counterions, as well as to investigate the impact of different structural features on the physicochemical and antimicrobial properties of the target salts. Moreover, introducing an ionic fragment into the salen structure changes its physicochemical properties and antimicrobial activity, depending on the cation structure and type of anion. Furthermore, by introducing an ionic core to a salen structure by quaternisation of amines with a modified salicylaldehyde structure, it will be possible to create organic salts with expanded structures. The second main objective of the research undertaken in this article is to assess the possibility of a synergistic effect of chiral salen-based organic salts and lytic bacteriophages in the biocontrol of selected saprophytic bacteria isolated from food matrices.

## 2. Results and Discussion

### 2.1. Synthesis of Chiral Salen Organic Salts

**Figure 1:**
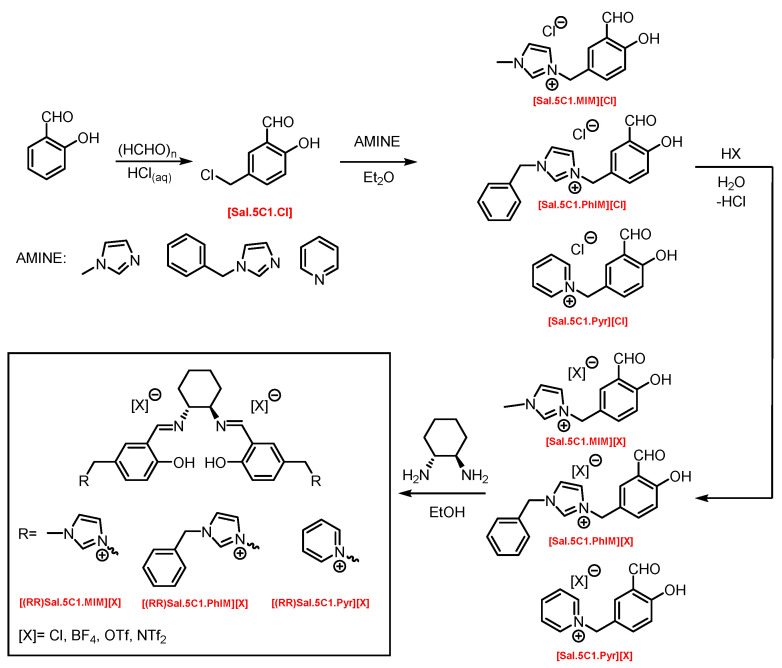
General synthesis scheme of chiral salen-based organic salts.

In a multistep synthesis, new chiral salen-based organic salts (SSO) with fragments of N-methylimidazole, N-benzylimidazole, and pyridine in their cation structures were prepared as shown in Figure 1.

In the first step, a salicylaldehyde was converted into 5-(2-chloromethyl)-salicylaldehyde [Sal.5C1.Cl] in the reaction between salicylaldehyde, paraformaldehyde, and concentrated hydrochloric acid. Then, the obtained product was used for the quaternisation of N-methylimidazole, N-benzylimidazole, and pyridine in anhydrous diethyl ether or toluene. In the next step, the chloride anion in the resulting organic salts was exchanged to other anions using an appropriate acid, namely tetrafluoroboric acid (HBF_4_), trifluoromethanesulfonic acid (CF_3_SO_3_H–HOTf), or bis(trifluoromethanesulfonyl)imide ((CF_3_SO_2_)_2_NH–HNTf_2_). In the last step, prepared organic salts with [Cl]^−^, [BF_4_]^−^, [OTf]^−^, and [NTf_2_]^−^ anions were used to synthesis chiral salen-based organic salts via a condensation with (R,R)-trans-1,2-diaminocyclohexane in anhydrous ethanol under an inert atmosphere (Figure 1). Each synthesis step proceeded with a high efficiency greater than 90%. The structure and purity of all salts were successfully characterised by NMR, IR, and UV–Vis analysis.

### 2.2. Identification

One of the most characteristic signals in the ^1^H NMR spectra for all synthesised chiral salen-based organic salts is a singlet corresponding to the -OH proton attached to the aromatic salen ring (Table 1). In general, this proton has a very similar chemical shift (13.50–13.66 ppm) in all salen salts. The small differences of 0.06–0.11 ppm can be seen with the type of anion. There is a lower chemical shift of the OH proton for salts with chloride than with other anions ([BF_4_]]^−^, [OTf]^−^ or [NTf_2_]^−^) and the same cation. However, considering SSO containing the same anion, the slightly higher chemical shift of the OH proton was found for salts that with pyridinium than in those with an N-benzylimidazolium or N-methylimidazolium moiety. It can be concluded that large anions and the pyridinium moiety have a greater effect on the deshielding of the OH proton in salen-based organic salts. The highest chemical shift of the OH proton was registered for the [(RR)Sal.5C1.Pyr][Cl] salt. Moreover, the signals of the two H12 protons located between the phenyl ring and the quaternary nitrogen atom exhibited the highest chemical shift (5.72–5.85 ppm) for salts with a pyridinium moiety, [(RR)Sal.5C1.Pyr][X], compared to salts with a methylimidazolium, [(RR)Sal.5C1.MIM][X], and benzimidazolium moiety, [(RR)Sal.5C1.PhIM][X]. The signals for the H6 proton showed almost identical chemical shifts. The type of anion did not significantly affect the chemical shift of protons, although the most notable differences in chemical shifts occurred for salts with the same structure of cation and [Cl]^−^ anions compared to other anions. The full characterisation of all protons in each compound is provided in the experimental section.

In the IR spectra, the absorption band at ~1630 cm^−1^ is characteristic of the stretching vibration of a C=N bond of the imine group. The broad absorption band observed at ~3350 cm^−1^ suggests the presence of -OH group and imine nitrogen. Moreover, the absorption band at ~1280 cm^−1^ confirms a medium stretch Ar-O bond. The value ~2860 cm^−1^ has been identified as the strong stretch -CH_2_ group between quaternary nitrogen and the salicylic ring.

### 2.3. Melting Point, TG, and DSC

Chiral salen-based organic salts with [Cl]^−^, [BF_4_]^−^, and [OTf]^−^ anions were yellow solids at room temperature, while those a comprising [NTf_2_]^−^ anion were highly viscous liquids orange in colour. The solid salts’ melting point and SSO’s glass transition temperature with an [NTf_2_]^−^ anion were determined (Table 2).

Melting points higher than 100 °C were determined for chiral salen-based organic salts with [BF_4_]^−^ anion and different cationic moieties. The highest melting point, 161–164 °C, was measured for a salt containing the pyridinium cation attached to the salene structure, [(RR)Sal.5C1.Pyr][BF_4_]. For [(RR)Sal.5C1.MIM][BF_4_] and [(RR)Sal.5C1.PhIM][BF_4_], the melting points were 138–140 °C and 147–150 °C, respectively. In the group of solids the lowest melting points were observed for salts with an [OTf]^−^ anion—they were 53–57 °C, 54–57 °C, and 58–62 °C, respectively, for [(RR)Sal.5C1.MIM][OTf], [(RR)Sal.5C1.PhIM][OTf], and [(RR)Sal.5C1.Pyr][OTf]. Salen-based organic salts with chloride anions melted at temperatures between those of the salts with a [BF_4_]^−^ anion and [OTf]^−^ anion, but still below 100 °C. Therefore, all chiral salen-based organic salts, except those with a [BF_4_]^−^ anion, can be classified as chiral salen-type ionic liquids. The salts with an [NTf_2_]^−^ anion were liquids at room temperature and transformed into a glassy state at temperatures ranging from 4.13 to 16.81 °C (Table 2).

In conclusion, the melting points of the chiral salen organic salts, the majority of which are solids at room temperature, except for the liquids salts with the [NTf_2_]^−^ anion, depend on the anion in their structure. The lowest melting point salts have an [OTf]^−^ anion, whereas the highest melting point salts have a [BF_4_]^−^ anion. The melting point increases in the following order of anions: [OTf]^−^ < [Cl]^−^ < [BF_4_]^−^. The cationic moiety has little effect on the melting point. However, for salts with the [BF_4_]^−^ anion, some differences in melting points for different cationic moieties are noticeable and can be arranged according to their descending melting point as follows: pyridinium (Pyr) > benzylimidazolium (PhIM) > methylimidazolium (MIM).

The chiral salen-based organic salts with the [NTf_2_]^−^ anion were the most thermally resistant—the onset temperatures of decomposition were 327.7 °C, 297.2 °C, and 280.6 °C for [(RR)Sal.5C1.PhIM][NTf_2_], [(RR)Sal.5C1.MIM][NTf_2_], and [(RR)Sal.5C1.Pyr][NTf_2_], respectively. The salts with other anions, namely [Cl]^−^, [BF_4_]^−^, and [OTf]^−^, with the same cationic moiety were less thermally stable. Their onset decomposition temperature was more than 100 °C lower. Moreover, salts with anions and the same cationic core decompose within a similar range. Furthermore, chiral organic salen salts decompose in multiple steps depending on the structure of the cation and anion. SSOs with a [Cl]^−^ anion decompose in two stages, SSOs with [OTf]^−^ and [NTf_2_]^−^ anions decompose in three stages, whereas SSOs with a [BF_4_]^−^ anion decompose in four stages.

All the synthesised chiral salen organic salts are optically active and exhibit specific optical rotation with the same sign (minus) as the chiral amine (1*R*,2*R*)-trans-cyclohexanediamine (Table 3). The highest values of specific rotation were observed in each group with the same cation for compounds containing the [Cl]^−^ anion, namely [(RR)Sal.5C1.MIM][Cl], [(RR)Sal.5C1.PhIM][Cl], and [(RR)Sal.5C1.Pyr][Cl], with values of −67.8, −46.4, and −45.1, respectively. Moreover, the absolute value of the specific rotation decreased as the size of the anion increased, as follows: [Cl]^−^ > [BF_4_]^−^ > [OTf]^−^ > [NTf_2_]^−^. In the case of [(rac)Sal.5C1.MIM][Cl], where racemic CHDA (±)-trans-cyclohexanediamine was used in the synthesis, a slight change in optical rotation of −3.7 was observed. This is a consequence of the purity of the commercially available CHDA, which contains a small excess of (1*R*,2*R*)-trans-cyclohexanediamine. The maximum absorbance in the UV–Vis spectra was registered at the same wavelength of λ_max_ 320–323 nm for all compounds.

### 2.4. Minimum Inhibitory Concentration (MIC) and Minimum Bactericidal Concentration (MBC) for the Tested Chiral Organic Salts and Their Substrates

The minimum inhibitory concentration (MIC) is the lowest concentration of an antibacterial agent, expressed in mg L^−1^ (or μg mL^−1^), that completely inhibits the visible growth of the test organism under strictly controlled in vitro conditions [35]. The bactericidal efficacy of the compounds was demonstrated by determining the MBC. According to international guidelines (American Society of Microbiology (ASM) or the National Committee for Clinical Laboratory Standards, NCCLS (currently the Clinical and Laboratory Standards Institute, CLSI)), a 99.9% reduction in bacteria is a criterion for bactericidal activity [36,37].

The MIC and MBC values for the compounds tested on the three strains of saprophytic bacteria are presented in Table 4. [(RR)Sal.5C1.PhIM][Cl], [(RR)Sal.5C1.PhIM][BF_4_], and [(RR)Sal.5C1.Pyr][OTf] were the compounds showing the lowest MIC values, ranging from 500 µg mL^−1^ for *S. fonticola* strain KKP 3685 to 2000 µg mL^−1^ for *E. cloacae* strain KKP 3692. The highest MIC values (>4000 µg mL^−1^) were determined for [(RR)Sal.5C1.Pyr][OTf] and [(RR)Sal.5C1.PhIM][NTf_2_] against *E. cloacae* strain KKP 3692. The values recorded for MBC were equal to or greater than the MIC values, ranging from 1000 µg mL^−1^ for [(RR)Sal.5C1.PhIM][OTf] against *S. fonticola* strain KKP 3685 and [(RR)Sal.5C1.PhIM][Cl] and [(RR)Sal.5C1.PhIM][BF_4_] against *E. coli* strain KKP 3688 to >4000 µg mL^−1^ for most compounds against *E. cloacae* strain KKP 3692.

The MIC and MBC values for all tested substrates for a given strain were equal: for *S. fonticola* strain KKP 3685 and *E. coli* strain KKP 3688, they were 2000 µg mL^−1^, while for the *E. cloacae* strain KKP 3692, they were 4000 µg mL^−1^. For the control compound, benzalkonium chloride (BC), the MIC and MBC values were the lowest for each tested strain, as they were 7.813 µg mL^−1^ (Table 4).

In the case of antibiotics, bacteria tolerant and intolerant to these compounds may have the same MIC value [35]. MBC is usually identical to or within one or two doubling dilutions of the MIC [37]. A strain is defined as tolerant when an antibiotic (or compound) at a concentration 32 times higher than the MIC does not contribute to a 99.9% reduction in the number of bacteria used in the test after 18–24 h of incubation [35,37]. The MBC/MIC ratio reflects the bactericidal capacity of the analysed compound [37]. For our compounds, it was not possible to determine the MBC/MIC ratio. For some compounds, the MIC or MBC values were higher than the highest tested compound concentration (i.e., 4000 µg mL^−1^).

Janiak et al. [34] tested the antibacterial activity of salen ligands imidazolium salts with a similar structure to our salts. Complexes with Mn(III) and Fe(III) were also examined by the authors. The tested compounds were derivatives of the racemic *trans*-1,2-diaminocyclohexane and salicylaldehyde or 3-tert-butylsalicylaldehyde, and have an imidazolium cation attached to a salicylidene backbone. The authors observed that the ligands as well as their complexes showed a significant degree of antibacterial activity against Gram-positive bacteria (*S. aureus*, *B. subtilis*), with MIC values between 8.74 µg mL^−1^ and >250 µg mL^−1^, and against *E. coli* from Gram-negative bacteria, with MIC values from 10.16 to >250 µg mL^−1^, while slight activity or no activity was observed toward *P. aeruginosa* (MIC from >250 to >10,000 µg mL^−1^). It is worth noting that the antibacterial activity of the compounds—similarly to that observed for our compounds—was highly dependent on the strain of the tested bacteria. When comparing the inhibition of *E.coli* growth, our compounds (Table 4) have slightly lower efficacy than those presented by Janiak et al. [34].

### 2.5. Influence of Chiral Salen-Based Organic Salts on the Bacteriophage Activity

**Figure 2:**
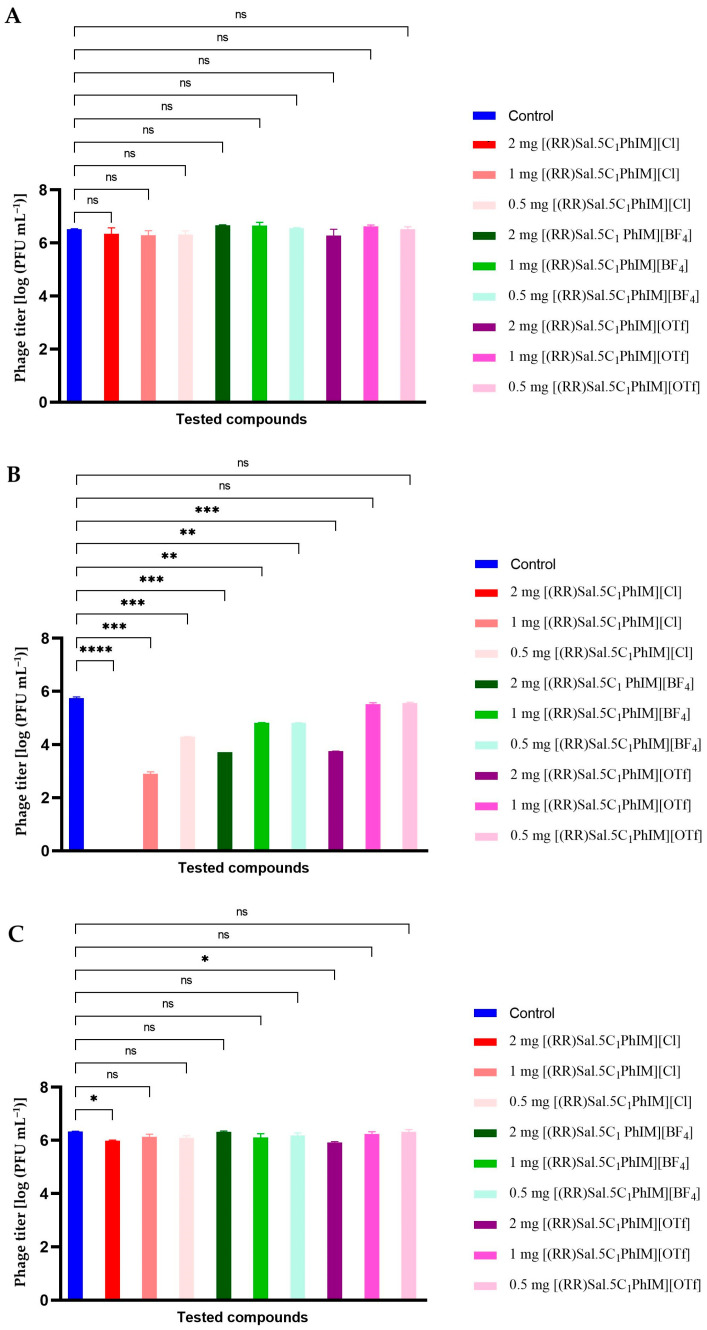
Influence of chiral organic salts on the bacteriophage activity: (**A**) Escherichia phage KKP 3710; (**B**) Serratia phage KKP 3712; (**C**) Enterobacter phage KKP 3716. **** means a significant difference (*p* ≤ 0.0001); *** means a significant difference (*p* ≤ 0.001); ** means a significant difference (*p* ≤ 0.01); * means a significant difference (*p* ≤ 0.05); ns means not significant (*p* > 0.05) of the control phage titre compared to the titre from the compound-treated phage lysate (*n* = 3).

The possibility of a synergistic effect of chiral organic salts in combination with lytic bacteriophages was assessed in the next stage. For this purpose, the effect of chiral organic salts, namely [(RR)Sal.5C1.PhIM]][Cl], [(RR)Sal.5C1.PhIM]][BF_4_] and [(RR)Sal.5C1.PhIM]][OTf] was tested in the initial stage at three different concentrations (i.e., 2 mg mL^−1^, 1 mg mL^−1^, and 0.5 mg mL^−1^) on the activity of three bacteriophages, namely Escherichia phage KKP 3710, Serratia phage KKP 3712, and Enterobacter phage KKP 3716 (Figure 2). The concentrations of the tested compounds were selected based on the experimentally determined MIC and MBC values.

The effect of the tested compounds on phage activity was strain-specific. In the case of Escherichia phage KKP 3710, none of the tested compounds at any of the three concentrations caused a significant reduction in the phage titre (Figure 2A). For Enterobacter phage KKP 3716, [(RR)Sal.5C1.PhIM]][Cl], and [(RR)Sal.5C1.PhIM]][OTf] at the highest concentration (2 mg mL^−1^) caused a significant (*p* ≤ 0.05) reduction in the phage titre. At the remaining concentrations, no significant reduction in the phage titre was observed (Figure 2C). The least stable was Serratia phage KKP 3712. Only [(RR)Sal.5C1.PhIM]][OTf] at a concentration of 1 mg mL^−1^ and 0.5 mg mL^−1^ did not cause a significant reduction in the phage titre. In all other variants, phage reduction was observed, with [(RR)Sal.5C1.PhIM]][Cl] at the highest concentration (2 mg mL^−1^) causing complete phage inactivation (Figure 2B).

These results indicate that when intending to use new antibacterial compounds in combination with bacteriophages, the effect of these compounds on inhibiting virus activity should be considered individually.

### 2.6. Synergistic Effect of Chiral Organic Salts and Lytic Bacteriophages Against Target Bacteria

**Figure 3:**
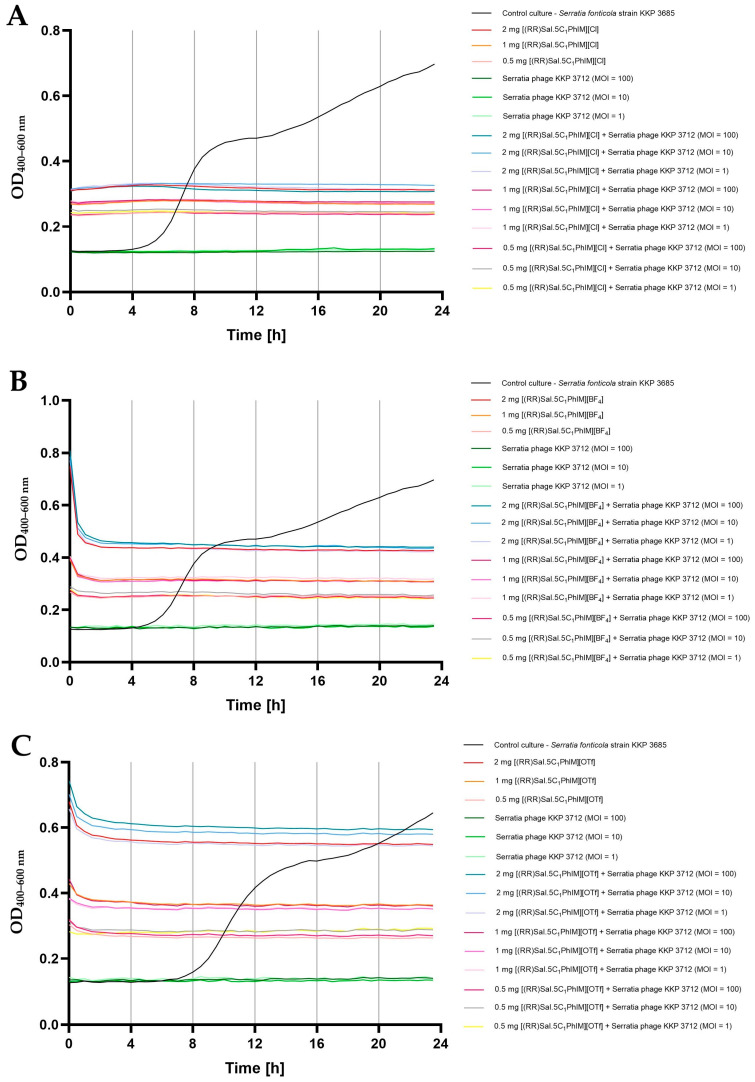
Synergistic effect of chiral organic salts and Serratia phage KKP 3712 against *Serratia fonticola* strain KKP 3685 (*n* = 5): (**A**) [(RR)Sal.5C1.PhIM][Cl]; (**B**) [(RR)Sal.5C1.PhIM][BF_4_]; (**C**) [(RR)Sal.5C1.PhIM][OTf].

**Figure 4:**
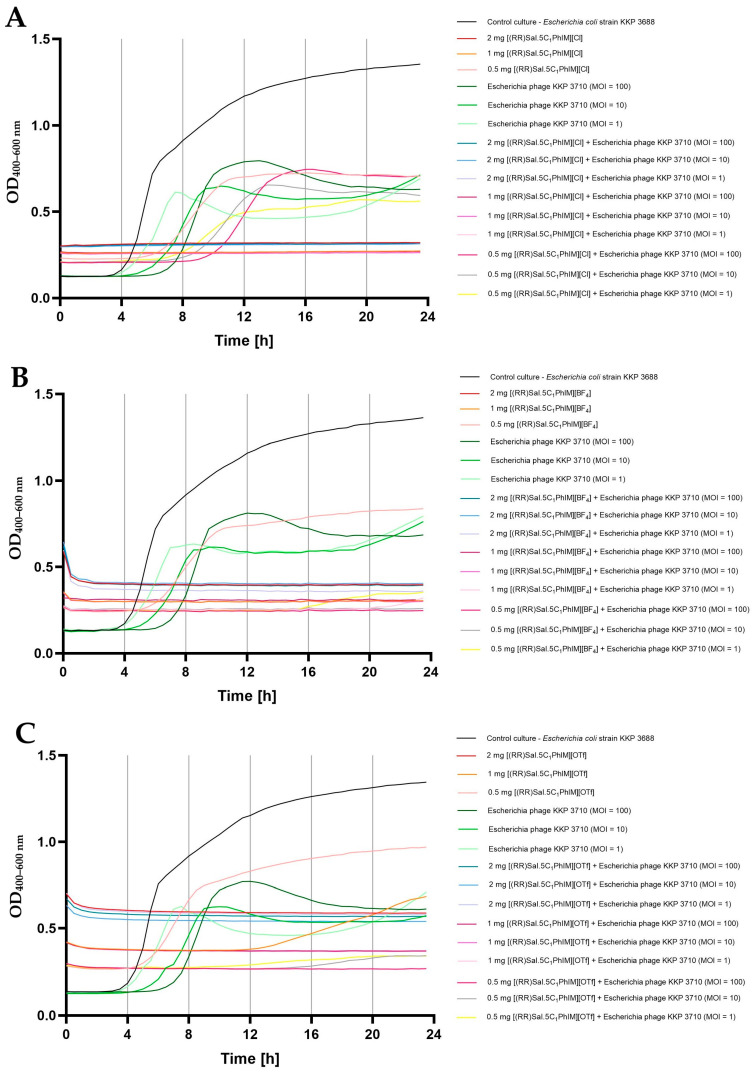
Synergistic effect of chiral organic salts and Escherichia phage KKP 3710 against *Escherichia coli* strain KKP 3688 (*n* = 5): (**A**) [(RR)Sal.5C1.PhIM][Cl]; (**B**) [(RR)Sal.5C1.PhIM][BF_4_]; (**C**) [(RR)Sal.5C1.PhIM][OTf].

**Figure 5:**
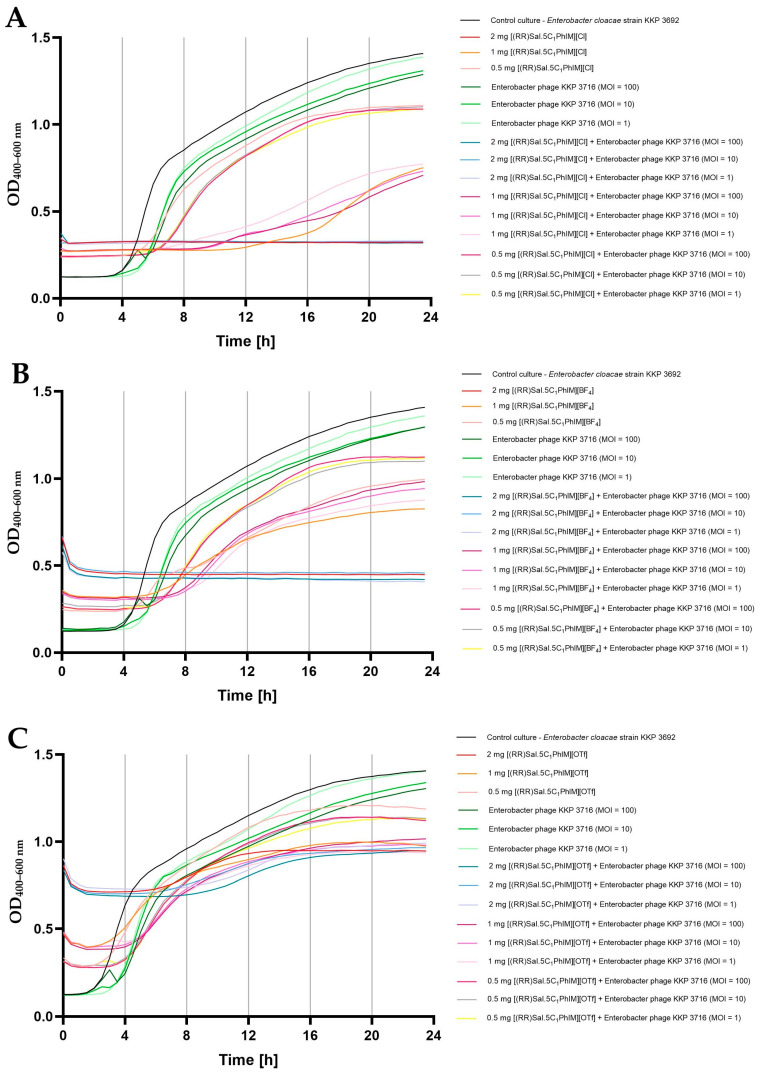
Synergistic effect of chiral organic salts and Enterobacter phage KKP 3716 against *Enterobacter cloacae* strain KKP 3692 (*n* = 5): (**A**) [(RR)Sal.5C1.PhIM][Cl]; (**B**) [(RR)Sal.5C1.PhIM][BF_4_]; (**C**) [(RR)Sal.5C1.PhIM][OTf].

In the final stage of this study, the effect of the combination of chiral organic salts, namely [(RR)Sal.5C1.PhIM]][Cl], [(RR)Sal.5C1.PhIM]][BF_4_] and [(RR)Sal.5C1.PhIM]][OTf], in three different concentrations (i.e., 2 mg mL^−1^, 1 mg mL^−1^, and 0.5 mg mL^−1^) in combination with bacteriophages (in three different MOIs, namely 100, 10, and 1) on the bacterial growth kinetics was determined (Figure 3, Figure 4 and Figure 5).

Figure 3 shows the growth kinetics of *S. fonticola* strain KKP 3685 after the action of the tested compounds, phages, and the combination of these antibacterial agents. Regardless of the agent used, the growth of bacteria was completely inhibited, regardless of the concentration of the compounds, as well as the number of added bacteriophage particles. The obtained effect is consistent with the experimentally determined MIC values of 500 µg mL^−1^ for [(RR)Sal.5C1.PhIM][Cl] and [(RR)Sal.5C1.PhIM][BF_4_], or 1000 µg mL^−1^ for [(RR)Sal.5C1.PhIM][OTf] (Table 4).

Figure 4 presents the growth kinetics of *E. coli* strain KKP 3688 following treatment with the tested compounds, phages, and their combination. The use of [(RR)Sal.5C1.PhIM]][Cl] and [(RR)Sal.5C1.PhIM]][BF_4_] at concentrations of 2 mg mL^−1^ and 1 mg mL^−1^ completely inhibited the growth of the tested bacteria (Figure 4A,B). The use of bacteriophages alone, regardless of the MOI, reduced but did not completely limit the growth of bacteria. In the case of using [(RR)Sal.5C1.PhIM][Cl] at a concentration of 0.5 mg mL^−1^, bacterial growth was observed at a level similar to that of the microculture treated with phages alone (Figure 4A). Although this compound did not negatively affect the activity of Escherichia phage KKP 3710 (Figure 2A), no synergistic effect with the phages in inhibiting bacterial growth was observed at any MOI. The addition of [(RR)Sal.5C1.PhIM][BF_4_] to microcultures at a concentration of 0.5 mg mL^−1^ did not completely limit the growth of bacteria (a stronger growth was observed compared to microcultures treated with phages alone). At the same time, a synergistic effect of this compound at a concentration of 0.5 mg mL^−1^ was observed in microcultures with Escherichia phage KKP 3710 (MOI = 10 and 100) on the complete inhibition of the growth of *E. coli* strain KKP 3688 (Figure 4B). No complete inhibition of growth of microcultures treated with [(RR)Sal.5C1.PhIM][OTf] at a concentration of 1 mg mL^−1^ and 0.5 mg mL^−1^ was demonstrated. Combining this compound at a concentration of 1 mg mL^−1^ with the addition of phages (at each MOI) and at a concentration of 0.5 mg mL^−1^ and MOI = 100 completely inhibited the growth of *E. coli* strain KKP 3688. In microcultures treated with [(RR)Sal.5C1.PhIM][OTf] at a concentration of 0.5 mg mL^−1^ and MOI = 10 or MOI = 1, a slight growth of bacteria was observed after 12 h, but at MOI = 100, the total inhibition of *E. coli* growth was found (Figure 4C). These results are also confirmed by the experimentally determined MIC values of the tested compounds against *E. coli* strain KKP 3688, which were 1000 µg mL^−1^ for [(RR)Sal.5C1.PhIM][Cl] and [(RR)Sal.5C1.PhIM][BF_4_] and 2000 µg mL^−1^ for [(RR)Sal.5C1.PhIM][OTf], respectively (Table 4).

Figure 5 displays the growth kinetics of *E. cloacae* strain KKP 3692 after exposure to the tested compounds, phages, and their combination. Treatment of *E. cloacae* strain KKP 3692 microcultures with phages alone, regardless of the MOI used, did not completely inhibit the growth of bacteria. The addition of [(RR)Sal.5C1.PhIM][Cl] and [(RR)Sal.5C1.PhIM][BF_4_] at the highest concentration (2 mg mL^−1^) completely limited the growth of bacteria, which confirms the experimentally determined MIC values of the tested compounds against *E. cloacae* strain KKP 3692 (Table 4). At the highest concentration of [(RR)Sal.5C1.PhIM][OTf], a slight increase in the optical density of the microcultures was noted.

In the case of [(RR)Sal.5C1.PhIM][Cl] and [(RR)Sal.5C1.PhIM][BF_4_] at concentrations of 1 mg mL^−1^ and 0.5 mg mL^−1^ and [(RR)Sal.5C1.PhIM][OTf] at a concentration of 1 mg mL^−1^, no synergistic effect was observed in combination with Enterobacter phage KKP 3716 regardless of the MOI (Figure 5). Additionally, no negative impact of these compounds on phage activity was detected (Figure 2C). [(RR)Sal.5C1.PhIM][OTf] at concentration of 0.5 mg mL^−1^ in combination with Enterobacter phage KKP 3716 limited the growth of *E. cloacae* KKP 3692 slightly more compared to the compound alone at this concentration (Figure 5C).

The results indicate that it is possible to combine both antibacterial agents, which will reduce the concentration of the chemical compound, but the effect is individually dependent on the specific bacteria.

It is hypothesised that the antibacterial activity of specific SSO is influenced by their amphiphilic nature, which may disrupt bacterial cell membranes, particularly through interactions with lipid bilayers. This membrane-targeting mechanism could vary with structural differences in the salts, particularly the nature of the cationic moiety (imidazolium, pyridinium, or benzimidazolium) and the associated counterion. For instance, salts with the [PhIM] cation and [Cl]^−^ or [BF_4_]^−^ anions showed the lowest MIC values, suggesting stronger interactions with bacterial surfaces and possibly better membrane penetration.

Moreover, the chirality of the diamine component in the salen structure may contribute to enantioselective binding to bacterial targets, such as membrane proteins or enzymes, potentially enhancing the antibacterial effect. Optical rotation measurements and structural characterization indicate that these chiral centres influence both the physical properties and bioactivity of the compounds.

In addition to their direct antibacterial effects, SSOs showed synergistic potential with bacteriophages, enabling reduced chemical concentrations while maintaining efficacy. This opens avenues for combination therapies targeting saprophytic and multidrug-resistant bacteria, especially in food safety contexts.

Altogether, understanding the structural basis of the antibacterial mechanisms of chiral organic salts will be crucial for the rational design of next-generation antimicrobial agents tailored to combat resistant pathogens.

## 3. Materials and Methods

### 3.1. Synthesis of Chiral Salen Organic Salts

#### 3.1.1. Materials Used in the Synthesis of Chiral Salen Organic Salts

All reagents were of analytical grade. Solvents, such as ethyl alcohol and toluene, were dried and stored over 3Å molecular sieves.

#### 3.1.2. Methods Used for the Characteristic of Chiral Salen Organic Salts

^1^H NMR and ^13^C NMR spectra were recorded using CDCl_3_ as a solvent with TMS as the internal standard on a BRUKER DPX-400 spectrometer (BRUKER, Billerica, MA, USA). LC-MS/MS analysis was performed using a Vanquish Flex UHPLC system connected to an Orbitrap Exploris 120 (UHPLC-HRMS) mass spectrometer (Thermo Fisher Scientific Inc., Waltham, MA, USA). A C18 chromatographic column (2.6 µm, 150 mm x 3.0 mm) and ACN as an eluent (flow 0.3 mL min^−1^) were used. High-resolution mass spectra (HRMS) spectra were recorded in a positive ESI+ and negative ESI− ionization mode. FT-IR (Fourier transform infrared) spectra were obtained using a Nicolet iS5 FTIR spectrometer (Thermo Fisher Scientific Inc., Waltham, MA, USA) with a DTGS detector and a platinum ATR sampling module with a solid diamond crystal. Spectra were collected in the frequency range of 4000–400 cm^−1^. For each sample, 16 scans with a resolution of 4 cm^−1^ were taken. The melting point was determined using the automatic melting point apparatus OPTIMELT (SRS - Stanford Research Systems, Sunnyvale, CA, USA). Thermogravimetric analysis was carried out using a thermo-microbalance TG 209 F1 Libra (Netzsch, Selb, Germany). The sample was loaded into an Al_2_O_3_ crucible and heated from 25 °C to 1000 °C at a rate of 5 K min^−1^, at a flow of air of 25 mL min^−1^ and a flow of nitrogen (as a purge gas) of 10 mL min^−1^. DSC curves for the chiral salts were registered using a Q100 Differential Scanning Calorimeter (TA Instruments, New Castle, DE, USA). The sample was loaded into an aluminium crucible within a temperature range of −90 °C ÷ 120 °C at a constant heating/cooling rate of 10 K min^−1^ in the nitrogen atmosphere. Optical rotation was measured using an automatic polarimeter AUTOPOL IV (Rudolph Research Analytical, Hackettstown, NJ, USA). Measurements were performed for DMSO solutions at 589 nm at 20 °C. The accuracy of measurement was ±0.002°. UV–Vis spectra were recorded in DMSO as a solvent on a microplate reader (Tecan, Infinite M Plex, Männedorf Switzerland) at wavelengths of 210–600 nm using a 96-hole quartz plate. The experimental data: ^1^H NMR, ^13^C NMR, UV-Vis spectra, and thermograms from thermogravimetric analysis (TG) were attached to Supplementary Materials.

#### 3.1.3. Synthesis Protocols

5-(2-chloromethyl)-salicylaldehyde, [Sal.5C1.Cl]

Here, 15 g of paraformaldehyde and 60 mL of 37% hydrochloric acid were added into a 500 mL two-necked round-bottomed flask equipped with a mechanical stirrer. Then, 18.3 g (150 mmol) of salicylaldehyde was added dropwise (around 1 drop per 3 s) and stirred for 72 h at room temperature. In the next step, 50 mL of cold distilled water was added and intensively stirred for 15 min. As a light-purple solid, the crude product was separated by vacuum filtration and washed with 3 × 5 mL of cold water. The yellow–grey solid was dried under reduced pressure at 50 °C for 24 h. Pure white crystal product was obtained by recrystallisation in hot hexane.
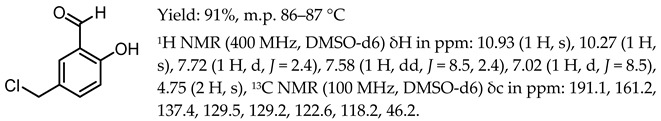


##### The General Procedure of Quaternisation Reaction

To an oven-dried 250 mL round-bottomed flask equipped with a magnetic stirrer, 10 g (0.0586 mol) of [Sal.5C1.Cl] was added to 100 mL of diethyl ether or toluene, dried over 3 Å molecular sieves, and intensively stirred until all the substrate was dissolved. Then, 0.615 mol of the appropriate amine (N-methylimidazole, pyridine, or N-benzylimidazole) in 30 mL of diethyl ether was added dropwise under an argon atmosphere. The reaction of quaternisation was carried out at room temperature for 24 h. The crude product was separated by vacuum filtration, washed 3× with 10 mL of dried diethyl ether, and dried overnight under reduced pressure at 40 °C. A pure light-yellow product was obtained by recrystallisation from hot isopropanol.

1-(3-formyl-4-hydroxybenzyl)-3-methylimidazolium chloride, [Sal.5C1.MIM][Cl]

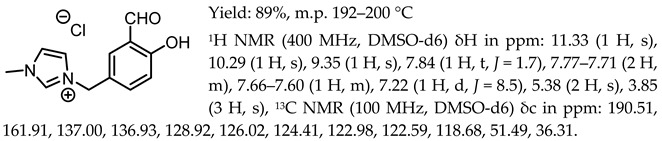

1-(3-formyl-4-hydroxybenzyl)-3-benzylimidazolium chloride, [Sal.5C1.PhIM][Cl]

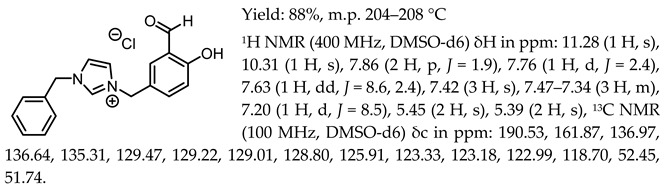

1-(3-formyl-4-hydroxybenzyl)-pyridinium chloride, [Sal.5C1.Pyr][Cl]

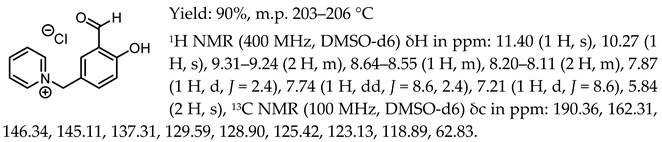


##### The General Procedure of Chloride Anion Exchange

In a 100 mL round-bottomed flask equipped with a magnetic stirrer, 0.01 mol of the appropriate chloride, i.e., [Sal.5C1.MIM][Cl], [Sal.5C1.PhIM][Cl], or [Sal.5C1.Pyr][Cl], was dissolved in 5 mL of deionised water. Then, 0.015 mol of the appropriate acid (HBF_4_, HOTf, or HNTf_2_) was added dropwise to a stirred chloride solution. The anion exchange was carried out for 24 h. The white solid was separated by vacuum filtration and washed several times with deionised water. The product was dried under reduced pressure for 24 h at 50 °C.

1-(3-formyl-4-hydroxybenzyl)-3-methylimidazolium tetrafluoroborate, [Sal.5C1.MIM][BF_4_]

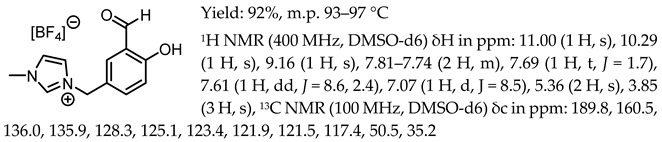

1-(3-formyl-4-hydroxybenzyl)-3-methylimidazolium trifluoromethanosulfonate, [Sal.5C1.MIM][OTf]

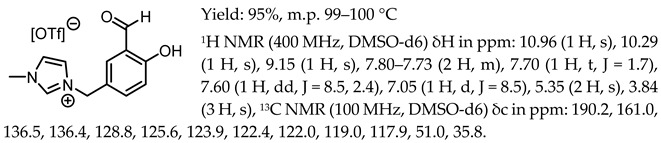

1-(3-formyl-4-hydroxybenzyl)-3-methylimidazolium bis(trifluoromethanesulfonyl)imide, [Sal.5C1.MIM][NTf_2_]

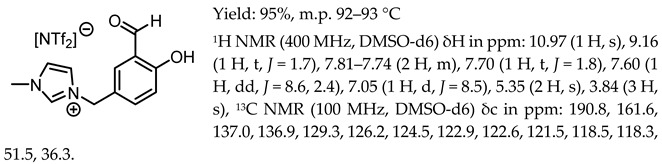

1-(3-formyl-4-hydroxybenzyl)-3-benzylimidazolium tetrafluoroborate, [Sal.5C1.PhIM][BF_4_]

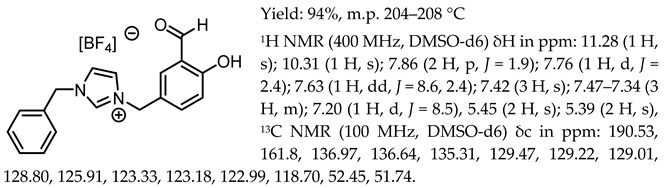

1-(3-formyl-4-hydroxybenzyl)-3-benzylimidazolium trifluoromethanesulfonate, [Sal.5C1.PhIM][OTf]

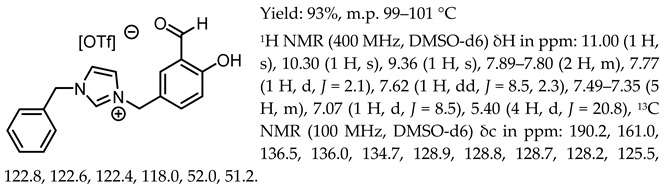

1-(3-formyl-4-hydroxybenzyl)-3-benzylimidazolium bis(trifluoromethylosulfonyl)imide, [Sal.5C1.PhIM][NTf_2_]

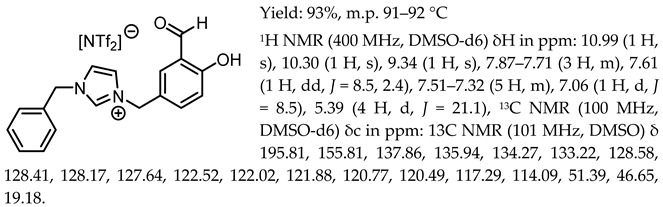

1-(3-formyl-4-hydroxybenzyl)-pyridinium tetrafluoroborate, [Sal.5C1.Pyr][BF_4_]

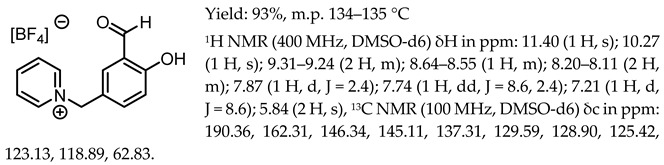

1-(3-formyl-4-hydroxybenzyl)-pyridinium trifluoromethanesulfonate, [Sal.5C1.Pyr][OTf]

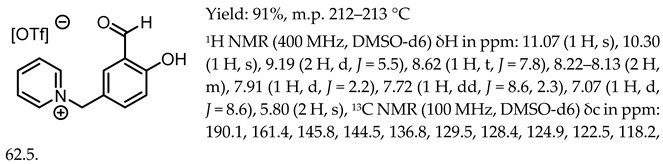

1-(3-formyl-4-hydroxybenzyl)-pyridinium bis(trifluoromethanesulfonyl)imide, [Sal.5C1.Pyr][NTf_2_]

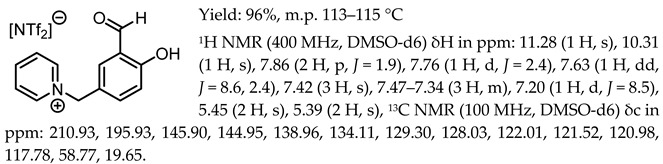


##### The General Procedure for the Synthesis of Chiral Salen Organic Salts

A 100 mL three-necked round-bottomed flask that had been oven-dried at 120 °C was equipped with a magnetic stir bar, doping funnel, cork with septa, and reflux condenser with a drying tube at the top. Then, 0.01 mmol of salt with [Cl]^−^, [BF_4_]^−^, [OTf]^−^ or [NTf_2_]^−^ anion was placed inside. Then, 5 mL of anhydrous ethyl alcohol was added, and the mixture was purged using argon as an inert gas. In a separate vial, 0.005 mmol of (1*R*,2*R*)-trans-cyclohexanediamine was weighed and dissolved in anhydrous ethyl alcohol. The ethanolic solution of CHDA was placed in a dropping funnel and dropped under an inert atmosphere (around 1 drop every 10 s). The reaction was carried out at 60 °C for 24 h. After this time, the solvent was evaporated, and the product as a yellow solid or as a viscous liquid in the case of SSOs with [NTf_2_]^−^ anion was washed with hexane, filtrated under reduced pressure, and dried overnight at 50 °C under reduced pressure. The pure product was recrystallised from ethyl acetate.

*N*,*N*′-bis-[5-((1-methylimidazol-3-ium)methylene)-salicylidene]-*trans*-(1*R*,2*R*)-cyclohexanediamine dichloride, [(RR)Sal.5C1.MIM][Cl]

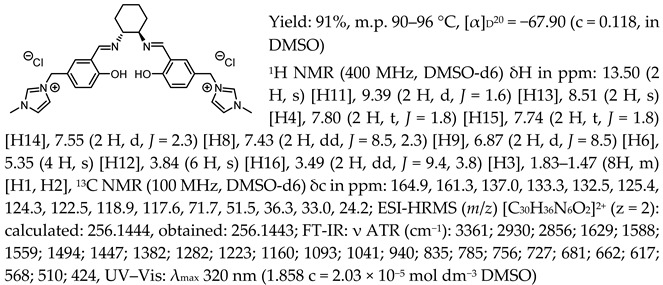

*N*,*N*′-bis-[5-((1-methylimidazol-3-ium)methylene)-salicylidene]-*trans*-(±)-cyclohexanediamine dichloride, [(RR)Sal.5C1.MIM][Cl]

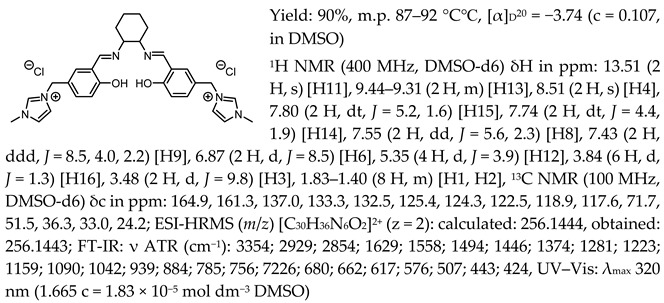

*N*,*N*′-bis-[5-((1-methylimidazol-3-ium)methylene)-salicylidene]-*trans*-(1*R*,2*R*)-cyclohexanediamine ditetrafluoroborate, [(RR)Sal.5C1.MIM][BF_4_]

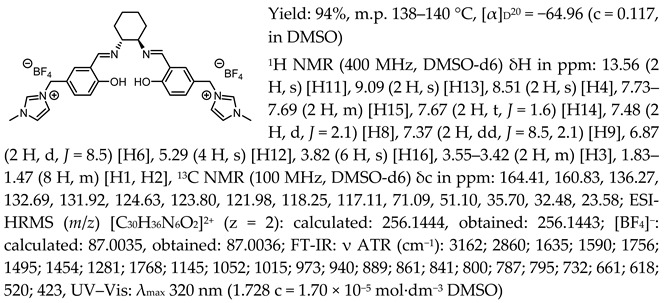

*N*,*N*′-bis-[5-((1-methylimidazol-3-ium)methylene)-salicylidene]-*trans*-(1*R*,2*R*)-cyclohexanediamine ditrifluoromethanesulfonate, [(RR)Sal.5C1.MIM][OTf]

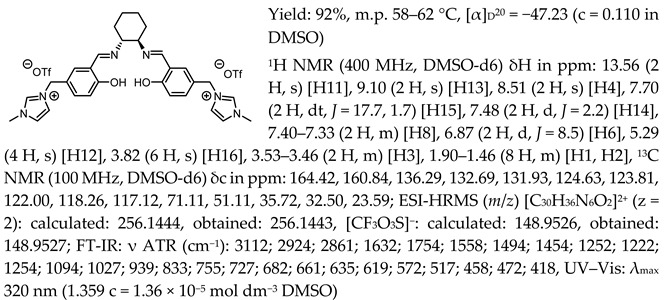

*N*,*N*′-bis-[5-((1-methylimidazol-3-ium)methylene)-salicylidene]-*trans*-(1*R*,2*R*)-cyclohexanediamine di[bis(trifluoromethanesulfonyl)imide], [(RR)Sal.5C1.MIM][NTf_2_]

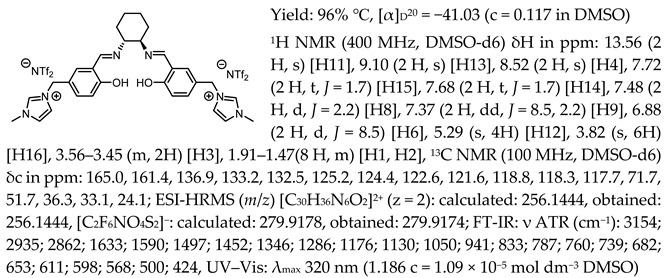

*N*,*N*′-bis-[5-((pyridinium)methylene)-salicylidene]-*trans*-(1*R*,2*R*)-cyclohexanediamine dichloride, [(RR)Sal.5C1.Pyr][Cl]

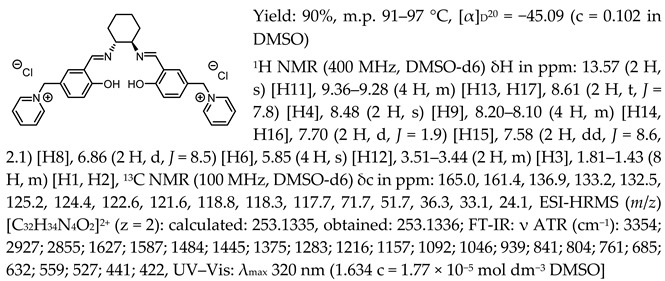

*N*,*N*′-bis-[5-((pyridinium)methylene)-salicylidene]-*trans*-(1*R*,2*R*)-cyclohexanediamine ditetrafluoroborate, [(RR)Sal.5C1.Pyr][BF_4_]

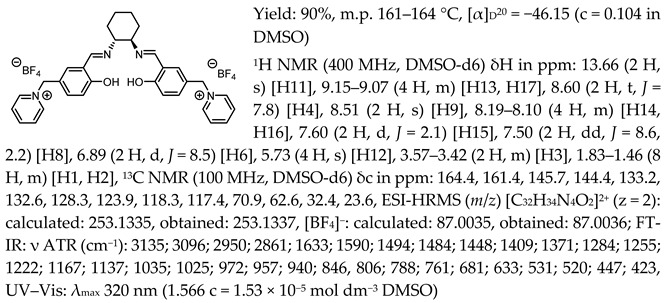

*N*,*N*′-bis-[5-((pyridinium)methylene)-salicylidene]-*trans*-(1*R*,2*R*)-cyclohexanediamine ditrifluoromethanesulfonate, [(RR)Sal.5C1.Pyr][OTf]

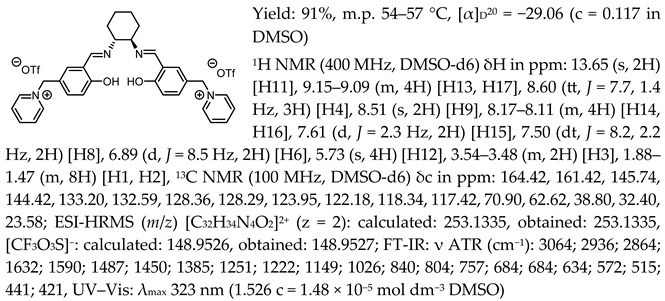

*N*,*N*′-bis-[5-((pyridinium)methylene)-salicylidene]-*trans*-(1*R*,2*R*)-cyclohexanediamine di[bis(trifluoromethanesulfonyl)imide], [(RR)Sal.5C1.Pyr][NTf_2_]

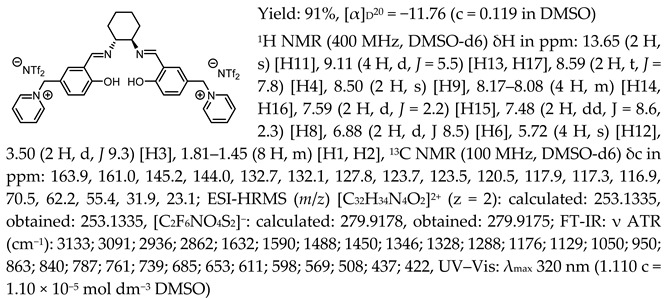

*N*,*N*′-bis-[5-((1-benzylimidazol-3-ium)methylene)-salicylidene]-*trans*-(1*R*,2*R*)-cyclohexanediamine dichloride, [(RR)Sal.5C1.PhIM][Cl]

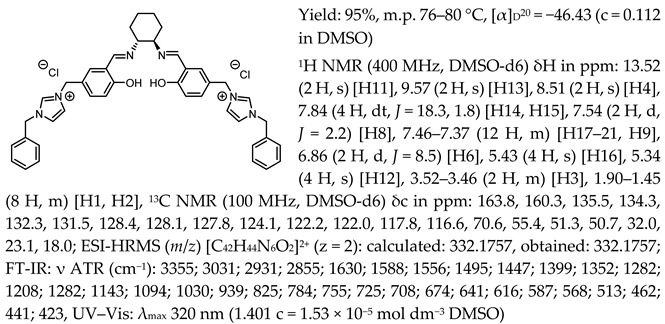

*N*,*N*′-bis-[5-((1-benzylimidazol-3-ium)methylene)-salicylidene]-*trans*-(1*R*,2*R*)-cyclohexanediamine ditetrafluoroborate, [(RR)Sal.5C1.PhIM][BF_4_]

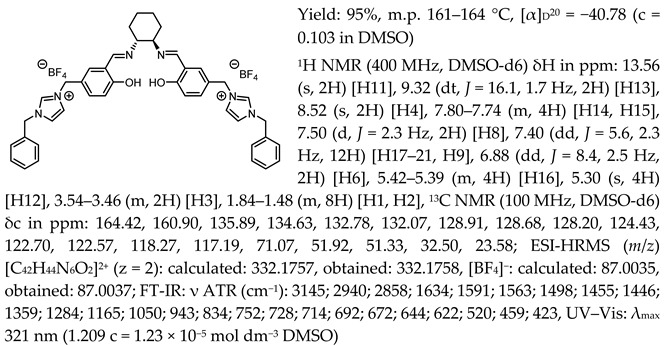

*N*,*N*′-bis-[5-((1-benzylimidazol-3-ium)methylene)-salicylidene]-*trans*-(1*R*,2*R*)-cyclohexanediamine ditrifluoromethanesulfonate, [(RR)Sal.5C1.PhIM][OTf]

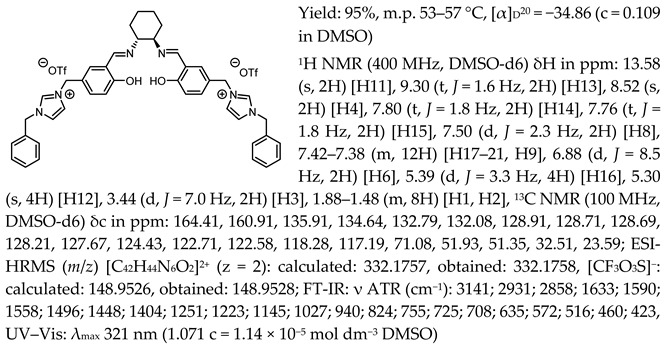

*N*,*N*′-bis-[5-((1-benzylimidazol-3-ium)methylene)-salicylidene]-*trans*-(1*R*,2*R*)-cyclohexanediamine di[bis(trifluoromethanesulfonyl)imide], [(RR)Sal.5C1.PhIM][NTf_2_]

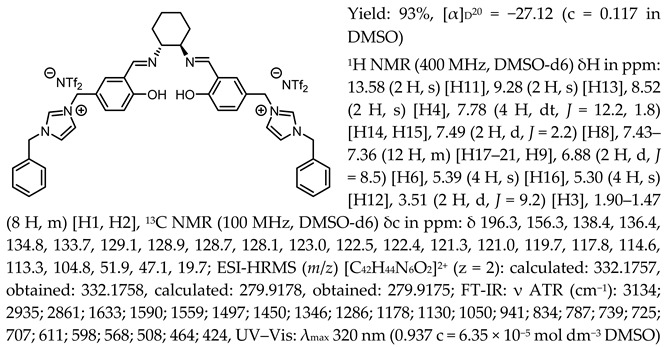


### 3.2. Microbiological Resources

The antibacterial activity of the tested compounds was examined on three selected strains of saprophytic bacteria isolated from minimally processed plant-based food products (ready-to-eat food products). The *Serratia fonticola* strain KKP 3685, *Escherichia coli* strain KKP 3688, and *Enterobacter cloacae* strain KKP 3692 were isolated from washed spinach, mixed leaf salad with beetroot, and unwashed spinach, respectively [6]. The studies revealed that the *S. fonticola* strain KKP 3685 exhibited phenotypic resistance to ampicillin and ceftaroline. In contrast, the *E. cloacae* strain KKP 3692 was resistant to ampicillin, sulbactam/ampicillin, amoxicillin/clavulanic acid, ceftaroline, ceftolozane/tazobactam, and ofloxacin (making it an MDR strain). In contrast, the *E. coli* strain KKP 3688 showed no phenotypic resistance to any antibiotics tested [6].

Specific lytic bacteriophages were used to assess the synergistic antibacterial effect of chiral organic salts, as follows: Serratia phage KKP 3712 targeted *S. fonticola* strain KKP 3685, Escherichia phage KKP 3710 targeted *E. coli* strain KKP 3688, and Enterobacter phage KKP 3716 targeted *E. cloacae* strain KKP 3692, which was isolated from municipal sewage as part of previously conducted research (unpublished data).

All bacterial and bacteriophage strains used in this research came from the Culture Collection of Industrial Microorganisms—Microbiological Resource Centre (in Polish: Kolekcja Kultur Drobnoustrojów Przemysłowych—Centrum Zasobów Mikrobiologicznych; KKP) of the Department of Microbiology at the Prof. Wacław Dąbrowski Institute of Agricultural and Food Biotechnology—State Research Institute (IAFB; Warsaw, Poland).

### 3.3. Determination of the Minimum Inhibitory Concentration (MIC)

The minimum inhibitory concentration (MIC) was determined by the microdilution method in Muller–Hinton culture broth (BTL, Lodz, Poland), according to the recommendations of the CLSI of the United States of America [38]. Chiral organic salts were used in the study, namely [(RR)Sal.5C1.MIM], [(RR)Sal.5C1.Pyr], and [(RR)Sal.5C1.PhIM], with four different anions, namely chloride [Cl]^−^, tetrafluoroborate [BF_4_]^−^, trifluoromethanesulfonate [OTf]^−^, and bis(trifluoromethylsulfonyl)imide [NTf_2_]^−^, as well as their substrates, namely CHDA (i.e., (1*R*,2*R*)-*trans*-cyclohexyldiamine), [Sal.5C1.PhIM][Cl], [Sal.5C1.PhIM][BF_4_], and [Sal.5C1.PhIM][OTf]. Benzalkonium chloride (BC) was used as a control.

All stock solutions were prepared under sterile conditions at 80 mg of the compound in 1 mL of dimethyl sulfoxide (DMSO; Merck, Darmstadt, Germany). An ultrasonic bath was used to dissolve each compound. Each prepared solution was filtered through a 0.22 μm syringe filter (Sartorius, Göttingen, Germany). Each stock solution was diluted 1:1 in Muller– Hinton broth to obtain a concentration of 40 mg mL^−1^. A series of 1:1 dilutions was then prepared in Muller–Hinton broth to obtain the following concentrations of the compounds: 40,000 μg mL^−1^; 20,000 μg mL^−1^; 10,000 μg mL^−1^; 5000 μg mL^−1^; 2500 µg mL^−1^; 1250 µg mL^−1^; 625 µg mL^−1^; 312.5 µg mL^−1^; 156.25 µg mL^−1^; 78.13 µg mL^−1^, and 39.06 µg mL^−1^.

A colony of each bacterial strain was collected from a Muller–Hinton agar plate (BTL, Lodz, Poland) and transferred to Muller–Hinton broth. Incubation was carried out at 37 °C for 18 h. Then, a bacterial suspension was prepared in Muller–Hinton broth to a final concentration of 5 × 10^5^ CFU mL^−1^ (previously, growth curves were prepared as a function of optical density at a 600 nm wavelength (OD_600_) and the number of colony-forming units (CFUs); unpublished data).

The experiment was performed in 96-well polystyrene plates (F type; Nest Scientific Biotechnology, Wuxi, China). The wells were filled to a volume of 200 µL, of which 180 µL was a suspension of a tested bacterial strain (5 × 10^5^ CFU mL^−1^), with 20 µL of the tested compound in a specified dilution (the final concentrations of each compound in the well were as follows: 4000 µg mL^−1^; 2000 µg mL^−1^; 1000 µg mL^−1^; 500 µg mL^−1^; 250 µg mL^−1^; 125 µg mL^−1^; 62.5 µg mL^−1^; 31.25 µg mL^−1^; 15.625 µg mL^−1^; 7.813 µg mL^−1^, and 3.906 µg mL^−1^).

Positive controls were wells with 180 µL of bacterial suspension and 20 µL of Muller–Hinton broth (to confirm bacterial growth) or 180 µL of bacterial suspension and 20 µL of DMSO (to exclude the influence of DMSO alone on the inhibition of bacterial growth). Negative controls were wells with 200 µL of Muller–Hinton broth. Cultures were performed at 37 °C for 18 h, and each variant was tested in triplicate (*n* = 3).

Growth or inhibition of bacterial growth was determined visually and by measuring the optical density of the suspension at 595 nm in a Synergy-H1 plate reader (BioTek, Agilent, Santa Clara, CA, USA). The MIC value was established as the minimum concentration of an antimicrobial substance required to prevent bacterial growth after 18 h of incubation at 37 °C. The limit of growth was considered to be 0.200 for OD_595_.

### 3.4. Determination of the Minimum Bactericidal Concentration (MBC)

The broth dilution method [38] was used to calculate the tested antimicrobials’ minimum bactericidal concentration (MBC). A volume of 10 µL was withdrawn with a loop from wells in multi-well plates (F type; Nest Scientific Biotechnology, Wuxi, China) in which no bacterial growth was observed after 18 h of incubation at 37 °C (when determining the MIC value) and then inoculated onto the surface of Muller–Hinton agar plates. Incubation was carried out at 37 °C for 18 h.

The MBC was considered the lowest concentration of the substance at which no bacterial colonies grew. Since the detection limit for this technique is 10 CFU mL^−1^, the absence of any growth on the plate indicated that the concentration was below this value. The initial concentration of ~10^5^ CFU mL^−1^ was, therefore, reduced to below CFU mL^−1^. Consequently, the MBC was considered the minimum concentration of antimicrobial agent capable of inactivating more than 99.9% of the bacteria initially present. Three independent replicates (*n* = 3) were performed for each bacterial strain and antimicrobial compound tested.

### 3.5. Synergistic Effect of Chiral Organic Salts and Other Antimicrobial Factors

In the next task of this study, the possibility of using an approach based on the synergistic effect of chiral organic salts with strain-specific bacterial viruses, called bacteriophages, was assessed.

#### 3.5.1. Influence of Chiral Organic Salts on the Bacteriophage Activity

The activity of bacteriophages in the broth with the addition of a selected chiral organic salt (i.e., [(RR)Sal.5C1.PhIM]) with three different anions, namely chloride [Cl]^−^, tetrafluoroborate [BF_4_]^−^, and trifluoromethanesulfonate [OTf]^−^, was assessed using the test tube method. For this purpose, 1.7 mL of Muller–Hinton broth, 200 µL of the test compound (at three different concentrations, namely 20,000 µg mL^−1^; 10,000 µg mL^−1^, and 5000 µg mL^−1^), and 100 µL of the specified bacteriophage (i.e., Escherichia phage KKP 3710, Serratia phage KKP 3712, and Enterobacter phage KKP 3716; each at a titre of ~10^7^ PFU mL^−1^) were added to each tube. Phage titre is the number of bacteriophage particles in 1 mL of lysate. As controls, 200 µL of the test compound was replaced by 200 µL of Muller–Hinton broth. Incubation was carried out at 37 °C for 24 h. The experiment was performed in three independent repetitions (*n* = 3).

Then, for each phage, its titre was compared to the control (after incubation in Muller–Hinton broth without adding any of the tested compounds). Phage titre was determined using the double-layer plate method described by [3]. Briefly, a series of ten-fold dilutions of phage lysates was prepared using a multi-well plate (well volume: 2 mL). To 500 µL of each phage lysate dilution, 100 µL of the host bacterial strain was added and left for 20 min. The entire phage suspension with bacteria was transferred to a Petri dish covered with a layer of Muller–Hinton agar and covered with 4 mL of soft agar (Muller–Hinton broth with 0.75% agar–agar (BTL, Lodz, Poland)). The solidified Petri dish plates were incubated at 37 °C for 24 h [3].

#### 3.5.2. Synergistic Effect of Chiral Organic Salts and Lytic Bacteriophages Against Target Bacteria

In the last stage, the synergistic effect of the chiral organic salts (i.e., [(RR)Sal.5C1.PhIM]) with three different anions, namely chloride [Cl]^−^, tetrafluoroborate [BF_4_]^−^, and trifluoromethanesulfonate [OTf]^−^, was determined along with the addition of specific phages. Serratia phage KKP 3712 was used against *S. fonticola* strain KKP 3685, Escherichia phage KKP 3710 was used against *E. coli* strain KKP 3688, and Enterobacter phage KKP 3716 was used against *E. cloacae* strain KKP 3692.

The study was performed in a Bioscreen C Pro automated growth analyser (Yo AB Ltd., Growth Curves, Helsinki, Finland) in dedicated 100-well plates. Microcultures were performed in a volume of 200 µL. The microcultures tested consisted of 160 µL of Muller–Hinton broth, 10 µL of host bacterial strain (10^5^ CFU mL^−1^), 20 µL of compound (in three different concentrations, namely 20,000 µg mL^−1^, 10,000 µg mL^−1^, or 5000 µg mL^−1^), and 10 µL of phage lysate (in Muller–Hinton broth) targeting a given bacterial strain (in a concentration of 10^7^ PFU mL^−1^, 10^6^ PFU mL^−1^, or 10^5^ PFU mL^−1^, providing three different multiplicity of infections, namely 100, 10, or 1). The multiplicity of infection (MOI) is the ratio of the number of bacteriophage particles to the number of bacterial host cells. Control microcultures consisted of the following: (1) bacteria alone (10 µL) in 190 µL of Muller–Hinton broth; (2) bacteria with the tested compound, where instead of the phage lysate, Muller–Hinton broth was added; and (3) bacteria with bacteriophage, where instead of the compound, Muller–Hinton broth was added.

The study was conducted at 37 °C for 24 h. The device measured optical density changes automatically every 30 min, with the microculture’s average shaking intensity immediately before the measurement. A broad-band filter (OD_400–600nm_) was used. Each study variant was performed in five replicates (*n* = 5).

### 3.6. Statistical Analysis

Each experiment was performed at least in triplicate (*n* = 3). Figure creation and statistical analyses of the presented results were performed in GraphPad Prism v8.0.2 (GraphPad Software Inc., San Diego, CA, USA). The multiple *t*-test followed by the two-stage step-up method of Benjamini, Krieger, and Yekutieli was used to assess the effect of the tested compounds on phage activity. Repeated measures two-way ANOVA with the Geisser–Greenhouse correction, and Dunnett’s multiple comparisons test, with individual variances computed for each comparison, were used to analyse the synergistic antibacterial effect of the tested compounds and phages.

## 4. Conclusions

New chiral salen-based organic salts were efficiently synthesised by attaching imidazolium and pyridinium cationic heads to a salicylidene backbone. The proposed synthesis method is effective and gives unlimited possibilities to introduce an ionic moiety to a salen ligand, followed by combining this with a different type of anion. All obtained salts were optically active and enantiomerically pure. The type of anion determines the physicochemical properties of the salts. The chiral salen organic salts with an [NTf_2_]^−^ anion were highly viscous liquids at room temperature and were characterised by the highest thermal stability. Moreover, the [(RR)Sal.5C1.MIM][Cl], [(rac)Sal.5C1.MIM][Cl], [(RR)Sal.5C1.MIM][OTf], [(RR)Sal.5C1.PhIM][Cl], [(RR)Sal.5C1.PhIM][OTf], [(RR)Sal.5C1.Pyr][Cl], and [(RR)Sal.5C1.Pyr][OTf] salts have a melting point below 100 °C, classifying them as ionic liquids. Novel chiral salen-based organic salts demonstrated varying levels of efficacy against *S. fonticola*, *E. coli*, and *E. cloacae*, with specific salts, such as [(RR)Sal.5C1.PhIM][Cl] and [(RR)Sal.5C1.PhIM][BF_4_], exhibiting the lowest MIC values. The synergistic potential between these salts (at specific concentrations) and bacteriophages was observed, offering a promising avenue for reducing the required concentrations of chemical agents while enhancing antibacterial effects. This study underscores the potential of chiral salen-based organic salts as viable alternatives to traditional antibiotics, particularly in combating antibiotic-resistant bacteria in food safety applications. Future research should focus on optimising these compounds for broader-spectrum applications and investigating their safety and efficacy in real-world environments.

## Figures and Tables

**Table 1:** ^1^H NMR chemical shifts of characteristic protons in chiral salen organic salts.

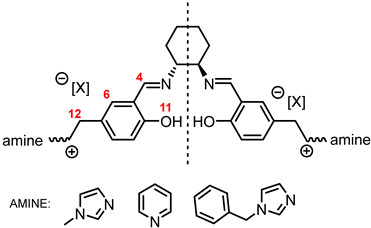				
	H4	H6	H11	H12
[(RR)Sal.5C1.MIM][Cl]	8.51	6.87	13.50	5.35
[(rac)Sal.5C1.MIM][Cl]	8.51	6.87	13.51	5.35
[(RR)Sal.5C1.MIM][BF_4_]	8.51	6.87	13.56	5.29
[(RR)Sal.5C1.MIM][OTf]	8.51	6.87	13.56	5.29
[(RR)Sal.5C1.MIM][NTf_2_]	8.52	6.88	13.56	5.29
[(RR)Sal.5C1.PhIM][Cl]	8.51	6.86	13.52	5.34
[(RR)Sal.5C1.PhIM][BF_4_]	8.52	6.88	13.56	5.30
[(RR)Sal.5C1.PhIM][OTf]	8.52	6.88	13.58	5.30
[(RR)Sal.5C1.PhIM][NTf_2_]	8.52	6.88	13.58	5.30
[(RR)Sal.5C1.Pyr][Cl]	8.61	6.86	13.57	5.85
[(RR)Sal.5C1.Pyr][BF_4_]	8.60	6.86	13.66	5.73
[(RR)Sal.5C1.Pyr][OTf]	8.60	6.89	13.65	5.73
[(RR)Sal.5C1.Pyr][NTf_2_]	8.59	6.88	13.65	5.72

**Table 2:** Melting point, thermal stability, and glass transition temperature of chiral salen-based organic salts.

Chiral Salen-Based Organic Salt	State *	T_m_ [°C]	T_g_ [℃]	T_onset_ [°C]
[(RR)Sal.5C1.MIM][Cl]	Solid	90–96	–^nd^	242.9
[(rac)Sal.5C1.MIM][Cl]	Solid	81–87	–^nd^	236.0
[(RR)Sal.5C1.MIM][BF_4_]	Solid	138–140	–^nd^	249.1
[(RR)Sal.5C1.MIM][OTf]	Solid	58–62	–^nd^	246.3
[(RR)Sal.5C1.MIM][NTf_2_]	Liquid	–^nd^	4.1	297.2
[(RR)Sal.5C1.PhIM][Cl]	Solid	76–80	–^nd^	246.4
[(RR)Sal.5C1.PhIM][BF_4_]	Solid	147–150	–^nd^	227.3
[(RR)Sal.5C1.PhIM][OTf]	Solid	53–57	–^nd^	219.5
[(RR)Sal.5C1.PhIM][NTf_2_]	Liquid	–^nd^	17.3	327.7
[(RR)Sal.5C1.Pyr][Cl]	Solid	91–97	–^nd^	181.9
[(RR)Sal.5C1.Pyr][BF_4_]	Solid	161–164	–^nd^	154.4
[(RR)Sal.5C1.Pyr][OTf]	Solid	54–57	–^nd^	190.5
[(RR)Sal.5C1.Pyr][NTf_2_]	Liquid	–^nd^	16.8	280.6

–^nd^—not determined; * at room temperature; T_m_—melting point determined by measurement in capillary; T_g_—glass transition temperature determined by DSC analysis; T_onset_—onset temperature of thermal decomposition determined by thermogravimetric analysis.

**Table 3:** Specific rotation and UV–Vis results of synthesised chiral salen-based organic salts.

Chiral Salen Organic Salt	Specific Rotation [α]_D_^20^	Absorbance * λ_max_
[(RR)Sal.5C1.MIM][Cl]	−67.8	320
[(rac)Sal.5C1.MIM][Cl]	−3.7	320
[(RR)Sal.5C1.MIM][BF_4_]	−64.9	320
[(RR)Sal.5C1.MIM][OTf]	−47.3	320
[(RR)Sal.5C1.MIM][NTf_2_]	−41.0	320
[(RR)Sal.5C1.PhIM][Cl]	−46.4	320
[(RR)Sal.5C1.PhIM][BF_4_]	−40.8	320
[(RR)Sal.5C1.PhIM][OTf]	−34.8	323
[(RR)Sal.5C1.PhIM][NTf_2_]	−27.1	320
[(RR)Sal.5C1.Pyr][Cl]	−45.1	320
[(RR)Sal.5C1.Pyr][BF_4_]	−40.8	321
[(RR)Sal.5C1.Pyr][OTf]	−34.9	321
[(RR)Sal.5C1.Pyr][NTf_2_]	−27.1	320

* c (~0.1 g mL^−1^) in DMSO.

**Table 4:** Minimum inhibitory concentration (MIC; µg mL^−1^) and minimum bactericidal concentration (MBC; µg mL^−1^) values for the tested chiral organic salts and their substrates determined for three strains of saprophytic bacteria.

Tested Compounds	*Serratia fonticola* Strain KKP 3685		*Escherichia coli* Strain KKP 3688		*Enterobacter cloacae* Strain KKP 3692	
	MIC [µg mL^−1^]	MBC [µg mL^−1^]	MIC [µg mL^−1^]	MBC [µg mL^−1^]	MIC [µg mL^−1^]	MBC [µg mL^−1^]
[(RR)Sal.5C1.MIM][Cl]	2000	2000	4000	4000	4000	>4000
[(rac)Sal.5C1.MIM][Cl]	1000	2000	4000	4000	4000	>4000
[(RR)Sal.5C1.MIM][BF_4_]	2000	2000	4000	4000	4000	>4000
[(RR)Sal.5C1.MIM][OTf]	1000	2000	4000	4000	4000	>4000
[(RR)Sal.5C1.MIM][NTf_2_]	2000	2000	2000	4000	2000	>4000
[(RR)Sal.5C1.Pyr][Cl]	1000	2000	2000	4000	4000	4000
[(RR)Sal.5C1.Pyr][BF_4_]	2000	2000	2000	4000	4000	>4000
[(RR)Sal.5C1.Pyr][OTf]	1000	4000	4000	4000	>4000	>4000
[(RR)Sal.5C1.Pyr][NTf_2_]	2000	2000	2000	4000	2000	>4000
[(RR)Sal.5C1.PhIM][Cl]	500	2000	1000	1000	2000	2000
[(RR)Sal.5C1.PhIM][BF_4_]	500	2000	1000	1000	2000	2000
[(RR)Sal.5C1.PhIM][OTf]	500	1000	2000	2000	2000	2000
[(RR)Sal.5C1.PhIM][NTf_2_]	1000	2000	2000	2000	>4000	>4000
Substrates						
CHDA	2000	2000	2000	2000	4000	4000
[Sal.5C1.PhIM][Cl]	2000	2000	2000	2000	4000	4000
[Sal.5C1.PhIM][BF_4_]	2000	2000	2000	2000	4000	4000
[Sal.5C1.PhIM][OTf]	2000	2000	2000	2000	4000	4000
Control compound						
BC	7.813	7.813	7.813	7.813	7.813	7.813

Notes: MIC—minimum inhibitory concentration; MBC—minimum bactericidal concentration; CHDA—(1*R*,2*R*)-(*trans*)-cyclohexanediamine; BC—benzalkonium chloride.

## Data Availability

The data presented in this study are included in the Supplementary Materials. Further inquiries can be directed to the corresponding author(s).

## Supplementary Materials

The following supporting information can be downloaded at: https://www.mdpi.com/article/10.3390/molecules30102173/s1, 1. Copies of 1H NMR and 13C NMR spectra of chiral salen organic salts; 2. Copies of FTIR spectra of chiral salen organic salts; 3. Curves from thermogravimetric analysis chiral salen organic salts.

## Abbreviations

The following abbreviations are used in this manuscript:

ASM	American Society of Microbiology
CLSI	Clinical and Laboratory Standards Institute
CFU	Colony-forming unit
DMSO	Dimethyl sulfoxide
IAFB	Prof. Wacław Dąbrowski Institute of Agricultural and Food Biotechnology—State Re-search Institute
MBC	Minimum bactericidal concentration
MIC	Minimum inhibitory concentration
MOI	Multiplicity of infection
NCCLS	National Committee for Clinical Laboratory Standards
OD	Optical density
PFU	Plaque-forming unit
SSO	Salen-based organic salts
WHO	World Health Organisation
