# Supplementary material for: Chiral Salen-Based Organic Salts: Synthesis and Potential Antibacterial Activity
Source: Molecules. 2025 May 15;30(10):2173. doi: 10.3390/molecules30102173 (PMC12114494; doi:10.3390/molecules30102173)

Chiral Salen-Based Organic Salts: Synthesis and Potential Antibacterial Activity

Marcin Gano [1,3*], Michał Wójcicki [2,†] and Ewa Janus [1,*]

[1] West Pomeranian University of Technology in Szczecin, Faculty of Chemical Technology and Engineering, Department of Organic Chemical Technology and Polymer Materials, Pułaskiego 10 St, 70-322 Szczecin, Poland; marcin.gano@zut.edu.pl [M.G.]; ewa.janus@zut.edu.pl (E.J.),

[2] Department of Microbiology, Prof. Wacław Dąbrowski Institute of Agricultural and Food Biotechnology – State Research Institute, Rakowiecka 36 St, 02-532 Warsaw, Poland; michal.wojcicki@ibprs.pl (M.W.)

[3] Center for Advanced Materials and Manufacturing Process Engineering (CAMMPE), Piastów, Ave. 45, 71-065 Szczecin, Poland; marcin.gano@zut.edu.pl (M.G.)

† Current address: Bacteriophage Laboratory, Department of Phage Therapy, Hirszfeld Institute of Immunology and Experimental Therapy, Polish Academy of Sciences, 53-114 Wrocław, Poland; michal.wojcicki@hirszfeld.pl

SUPPLEMENTARY MATERIAL

Table of content

1. Copies of ^{1}H NMR and ^{13}C NMR spectra of chiral salen organic salts 2
2. Copies of FTIR spectra of chiral salen organic salts 15
3. Curves from thermogravimetric analysis chiral salen organic salts 20

1. **Copies of ¹H NMR and ¹³C NMR spectra of chiral salen organic salts.**

N,N'-bis-[5-((1-methylimidazol-3-ium)methylene)-salicylidene]-*trans*-(1*R*,2*R*)-cyclohexanediamine dichloride, **[(RR)Sal.5C1.MIM][Cl]** in DMSO-d6

[(RR)Sal.5C1.MIM][Cl]

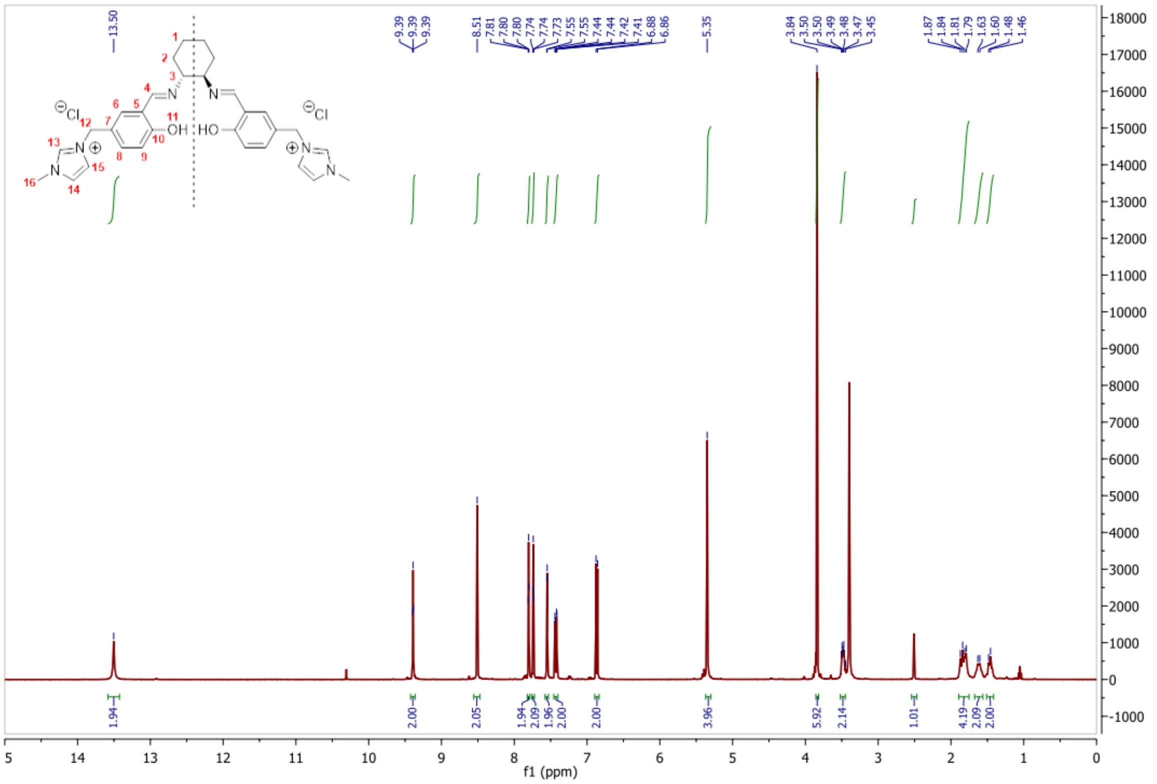

[(RR)Sal.5C1.MIM[Cl]

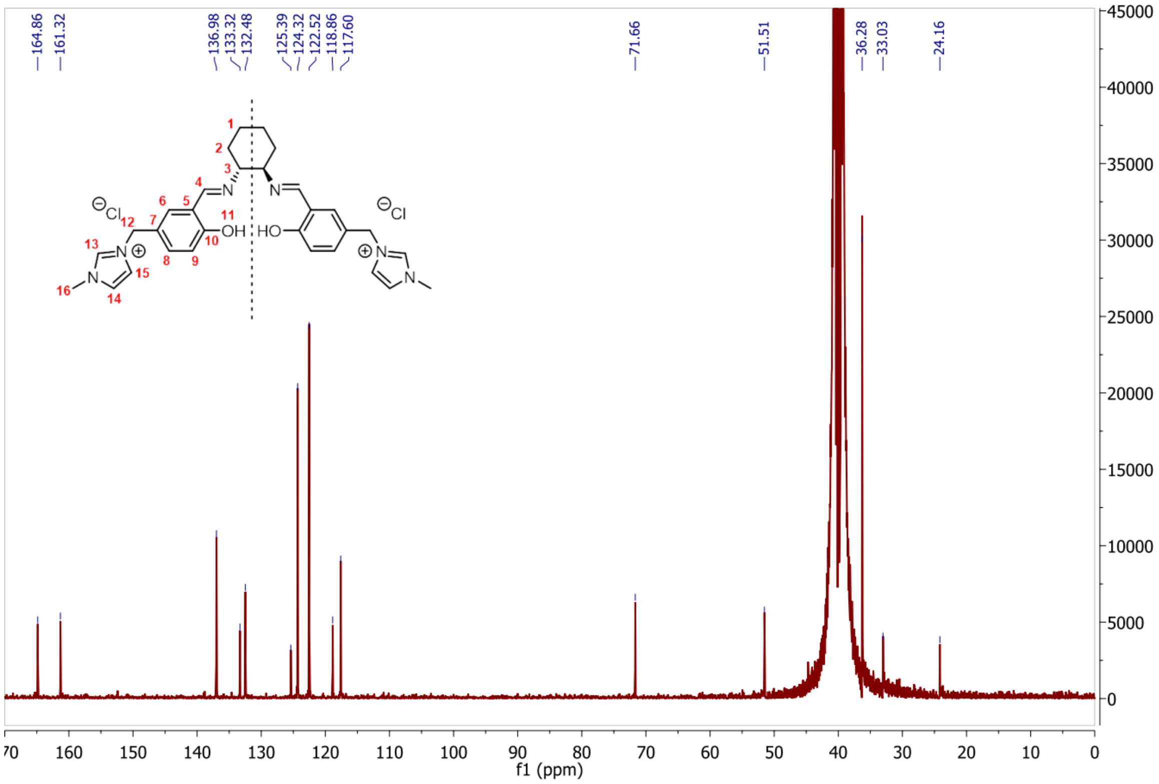

N,N'-bis-[5-((1-methylimidazol-3-ium)methylene)-salicylidene]-*trans*-(±)-cyclohexanediamine dichloride, **[(rac)Sal.5C1.MIM][Cl]** in DMSO-d6

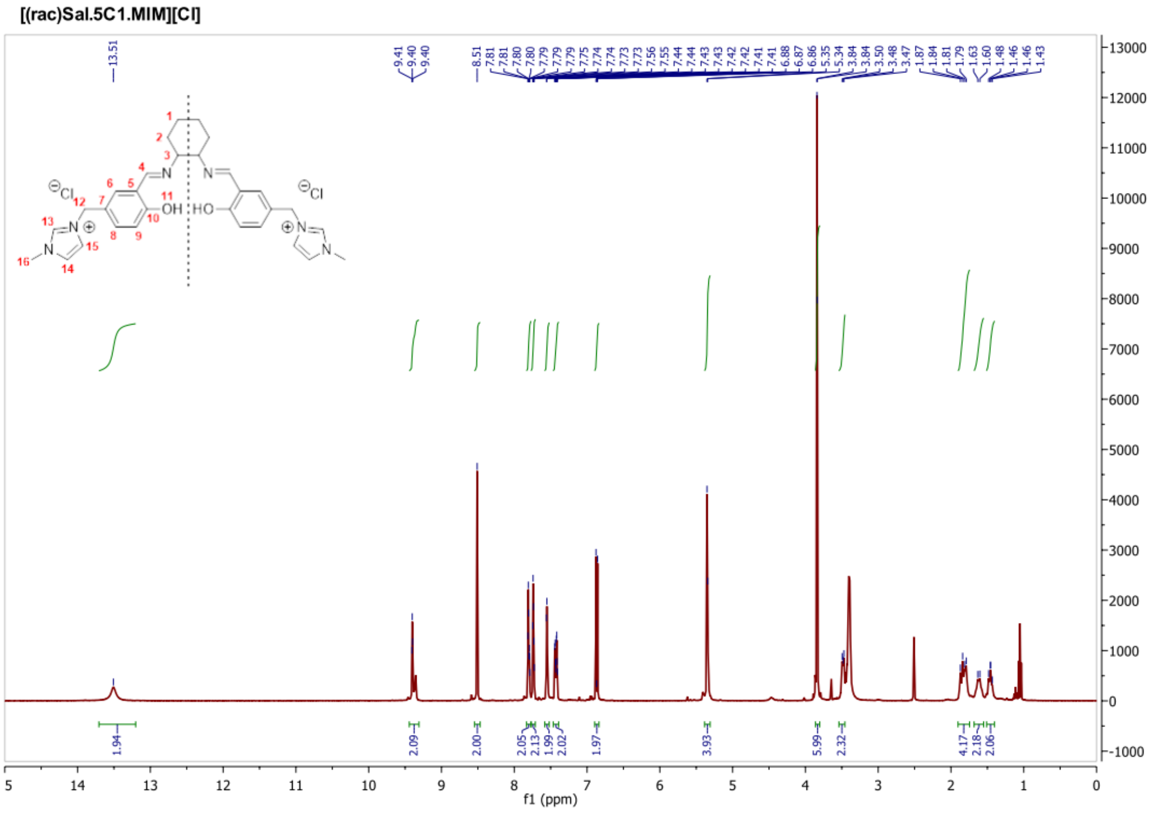

`

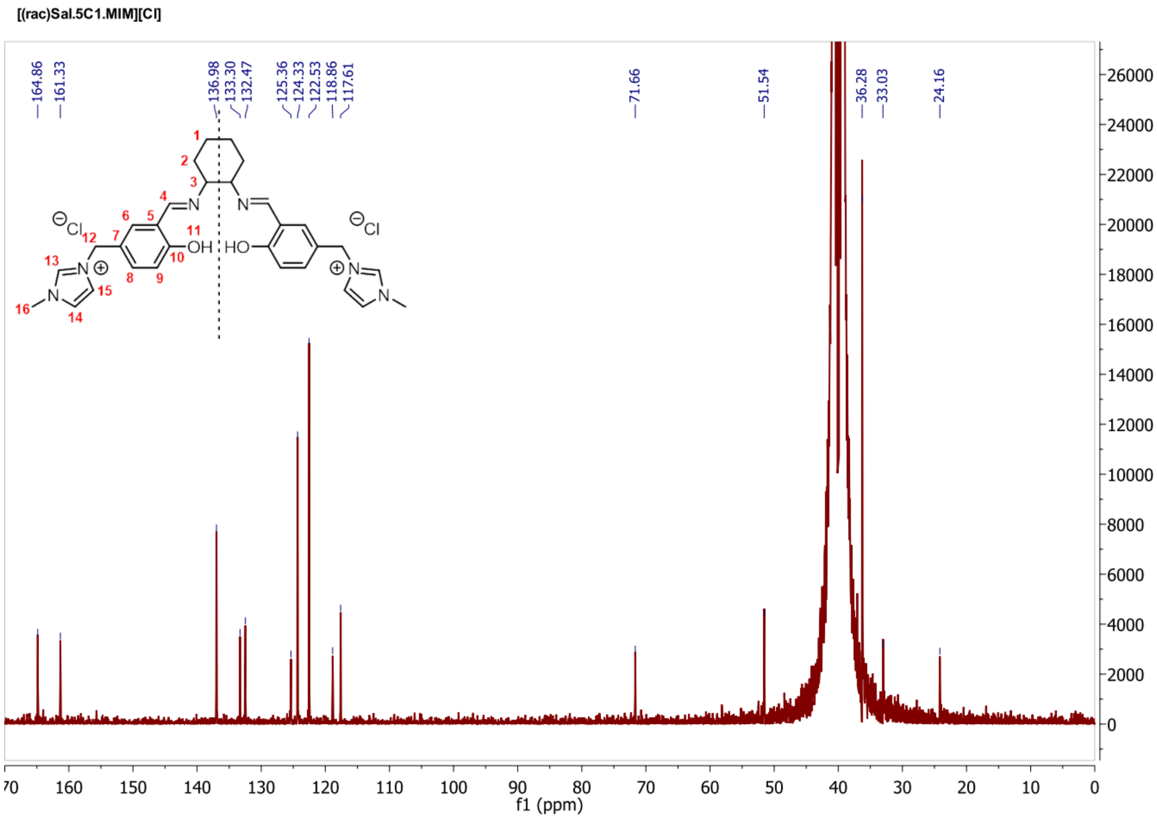

N,N′-bis-[5-((1-methylimidazol-3-ium)methylene)-salicylidene]-*trans*-(1*R*,2*R*)-cyclohexanediamine ditetrafluoroborate, **[(RR)Sal.5C1.MIM][BF$_4$]** in DMSO-d6

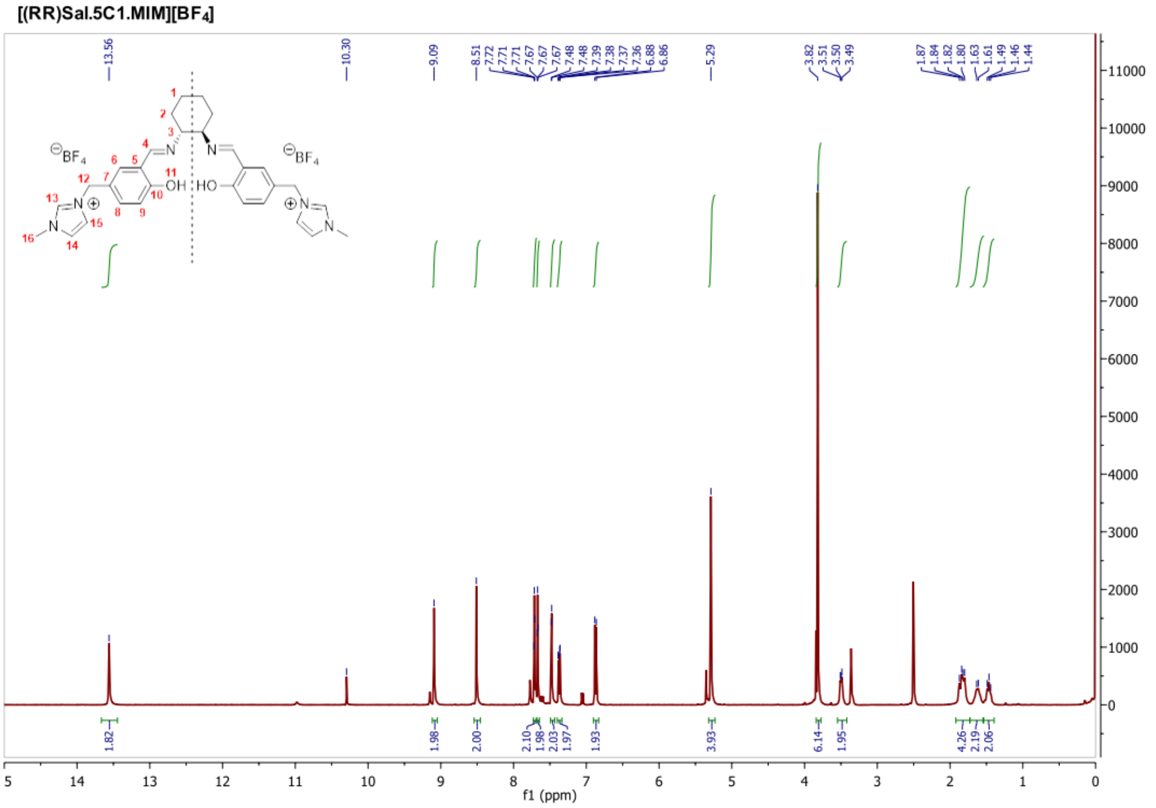

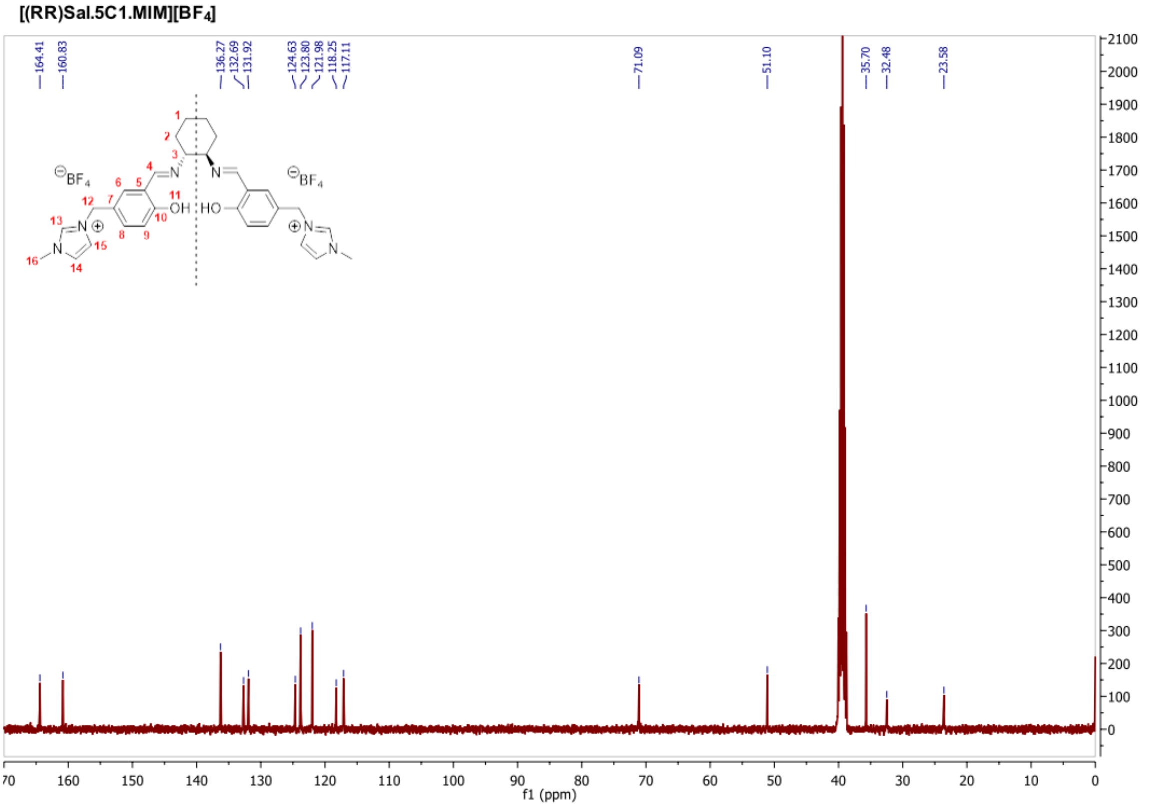

N,N′-bis-[5-((1-methylimidazol-3-ium)methylene)]-salicylidene]-*trans*-(1*R*,2*R*)-cyclohexanediamine ditrifluoromethanesulfonate **[(RR)Sal.5C1.MIM][OTf]** in DMSO-d6

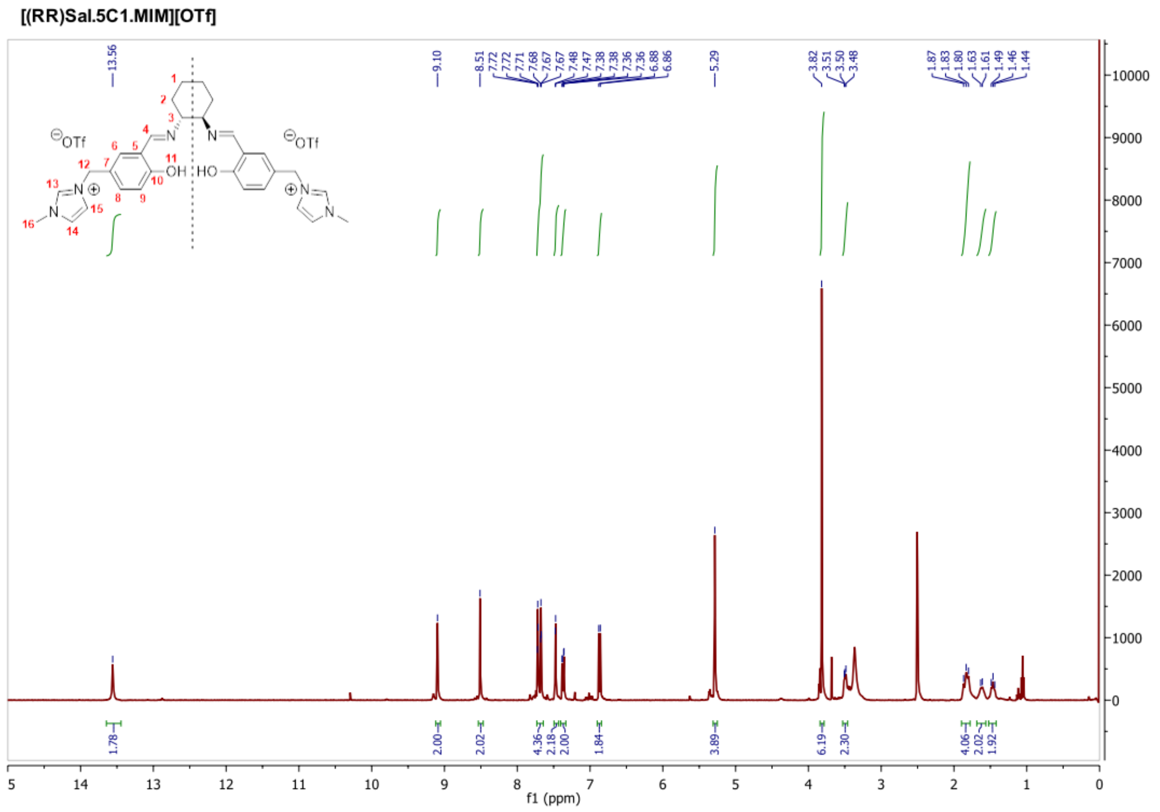

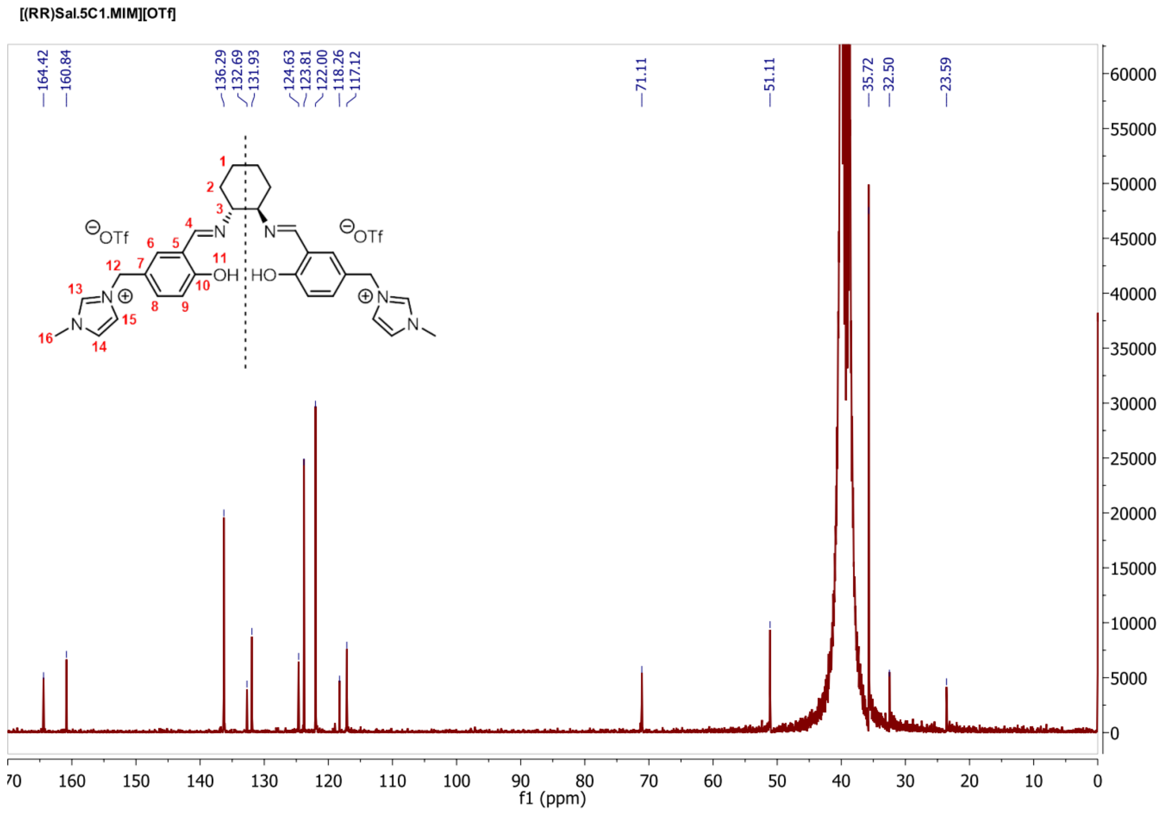

N,N′-bis-[5-((1-methylimidazol-3-ium)methylene)-salicylidene]-*trans*-(1*R*,2*R*)-cyclohexanediamine di[bis(trifluoromethanesulfonyl)imide], **[(RR)Sal.5C1.MIM][NTf₂]** in DMSO-d6

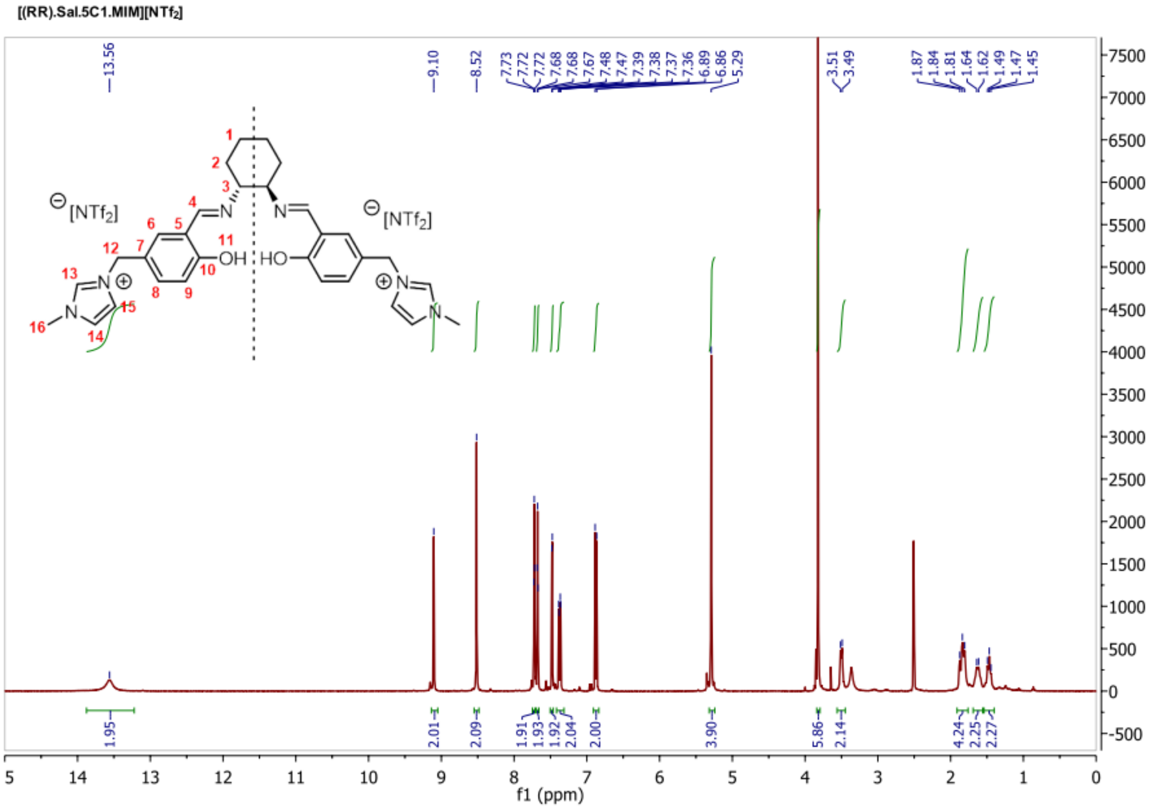

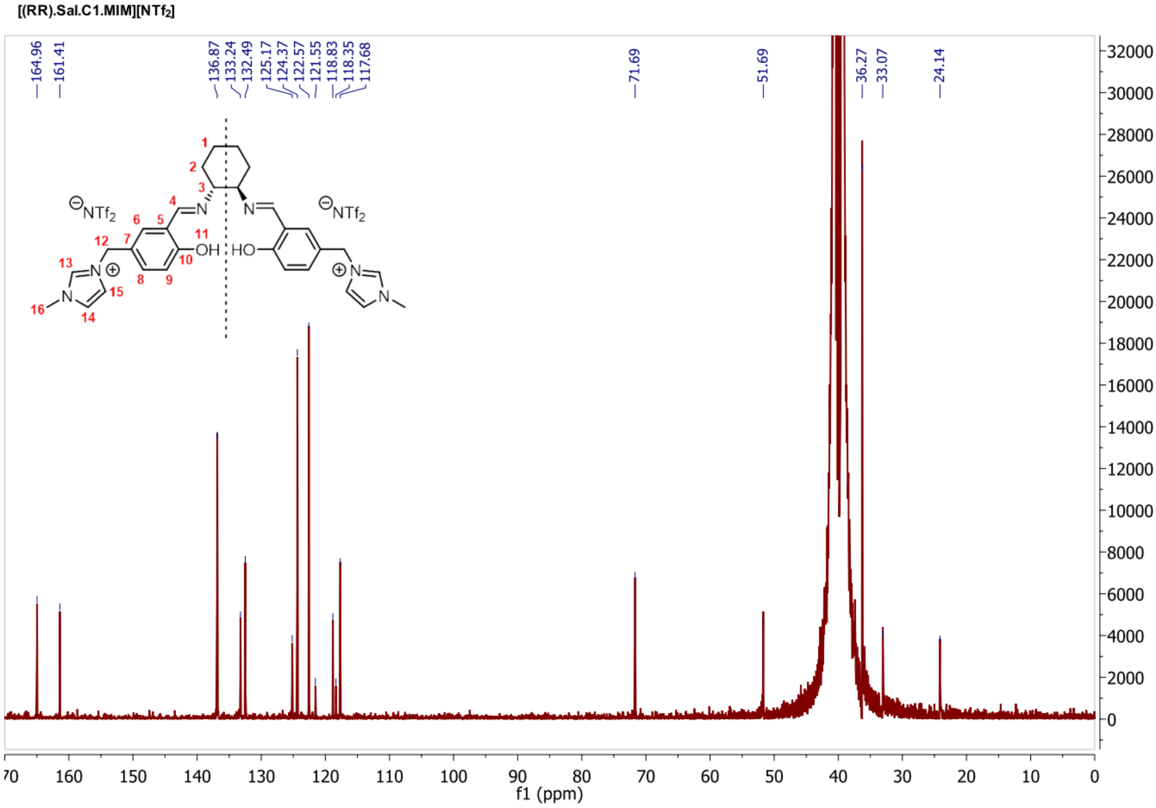

N,N'-bis-[5-((1-benzylimidazol-3-ium)methylene)-salicylidene]-*trans*-(1*R*,2*R*)-cyclohexanediamine dichloride, **[(RR)Sal.5C1.PhIM][Cl]** in DMSO-d6

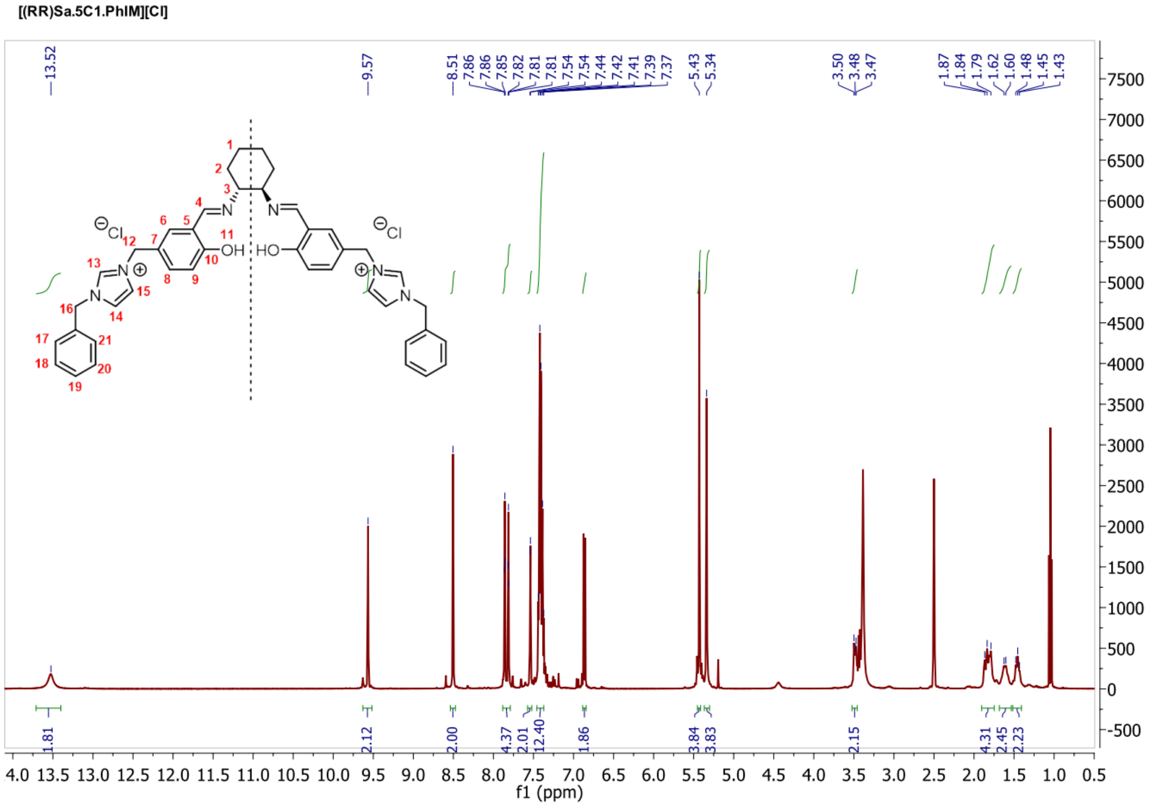

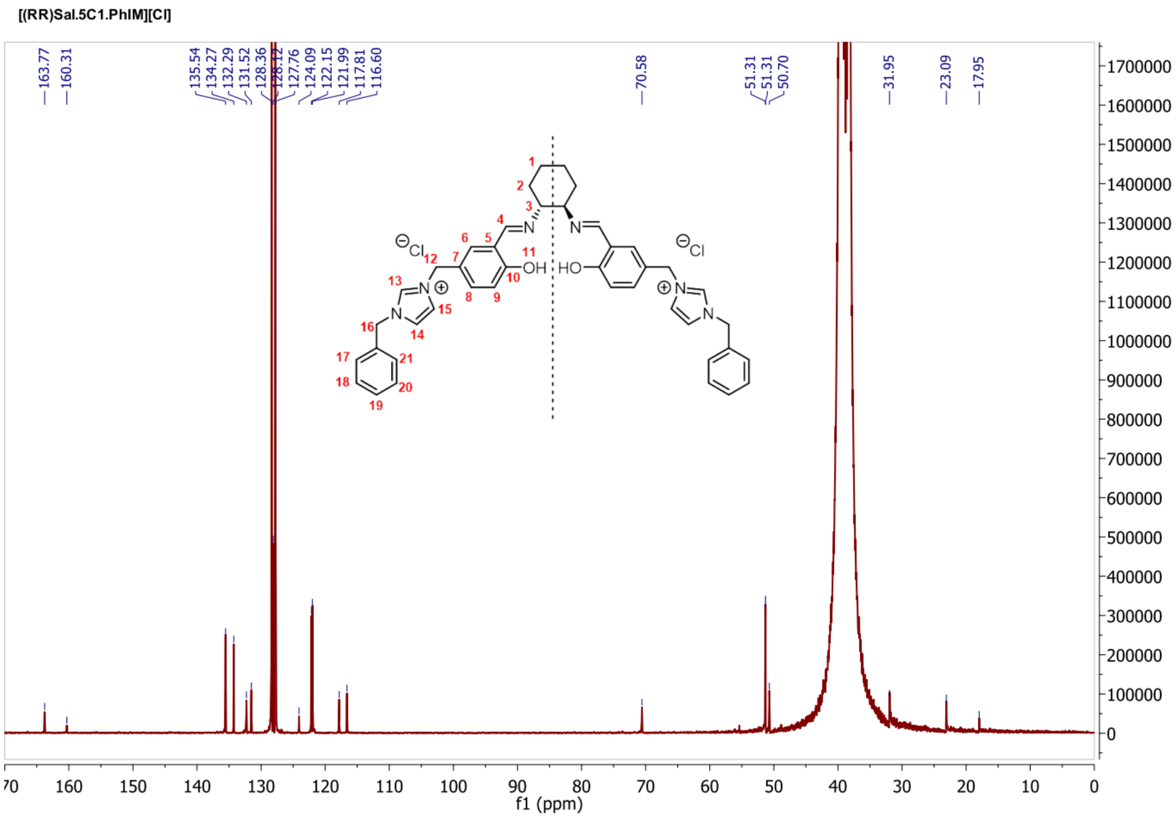

N,N′-bis-[5-((1-benzylimidazol-3-ium)methylene)-salicylidene]-*trans*-(1*R*,2*R*)-cyclohexanediamine ditetrafluoroborate, **[(RR)Sal.5C1.PhIM][BF4]** in DMSO-d6

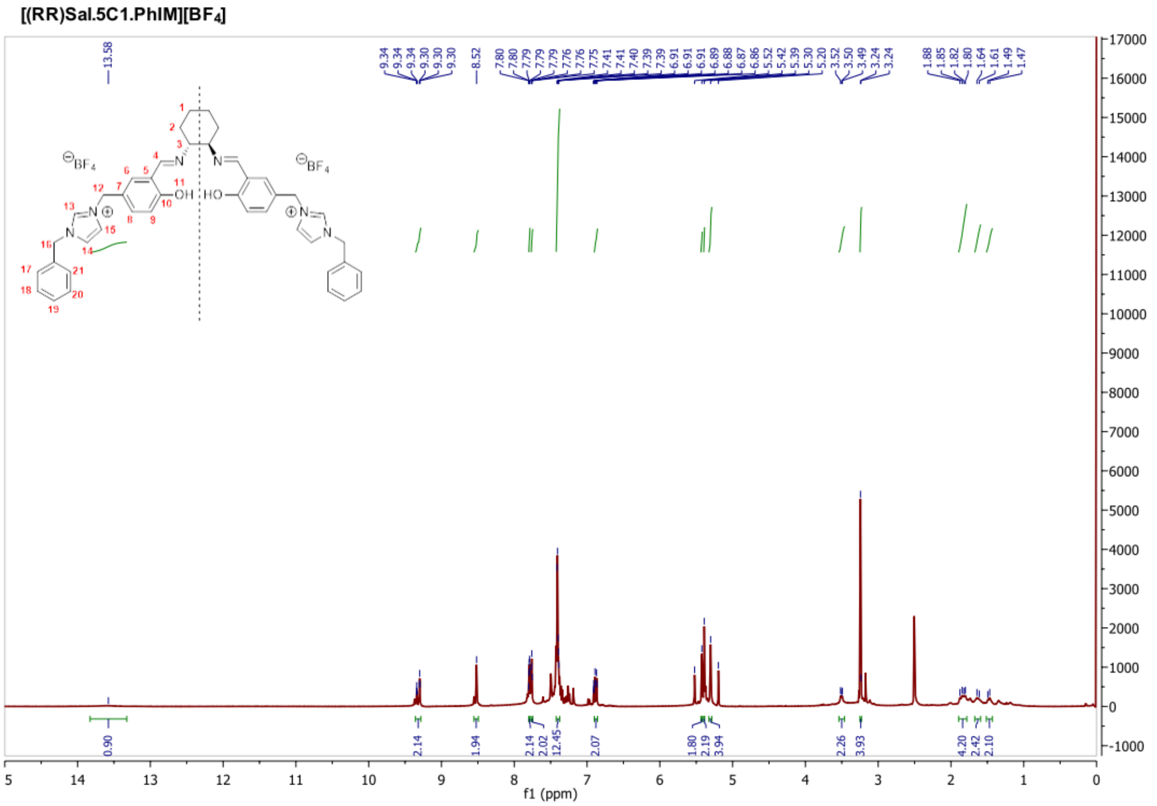

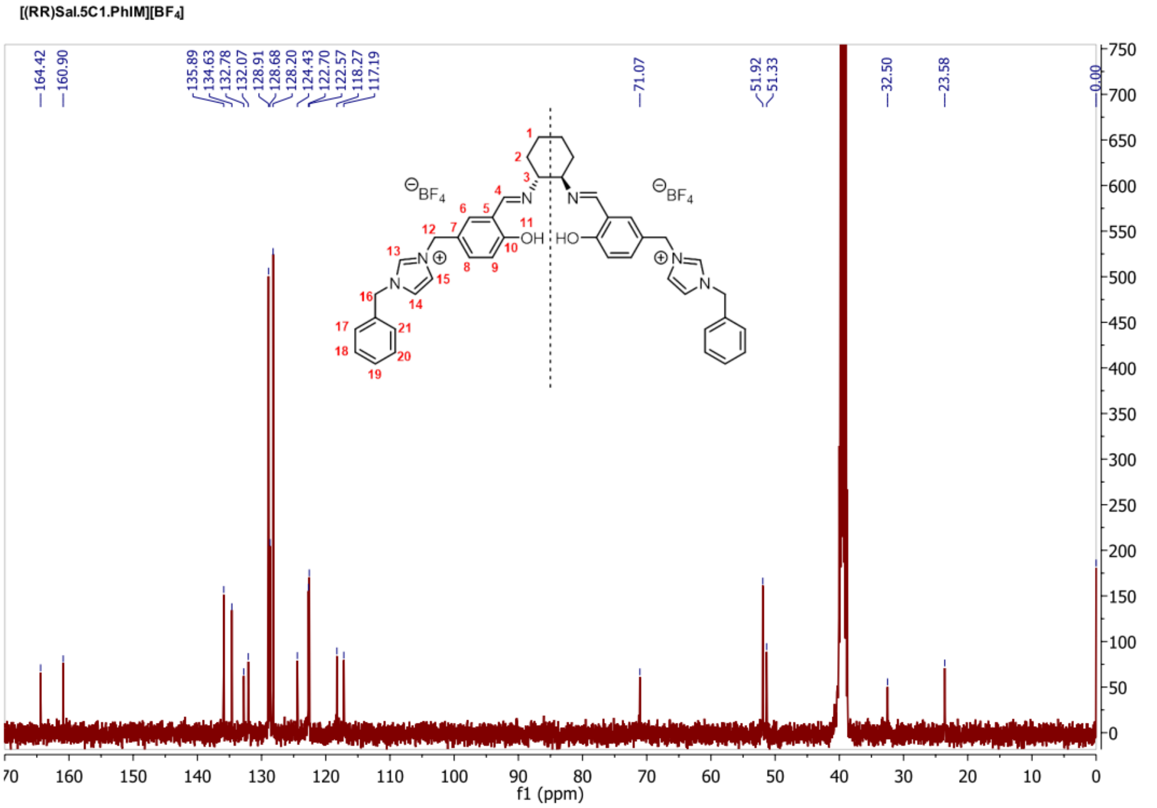

N,N'-bis-[5-((1-benzylimidazol-3-ium)methylene)-salicylidene]-*trans*-(1*R*,2*R*)-cyclohexanediamine ditrifluoromethanesulfonate, **[(RR)Sal.5C1.PhIM][OTf]** in DMSO-d6

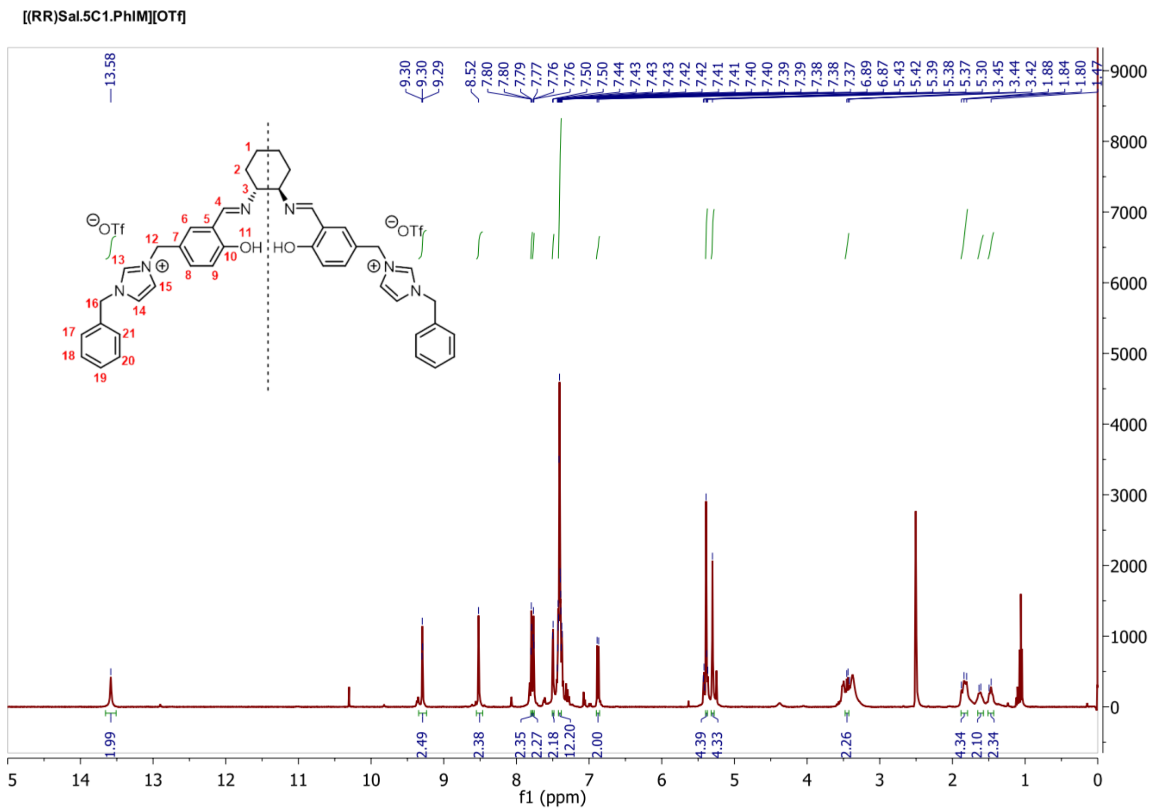

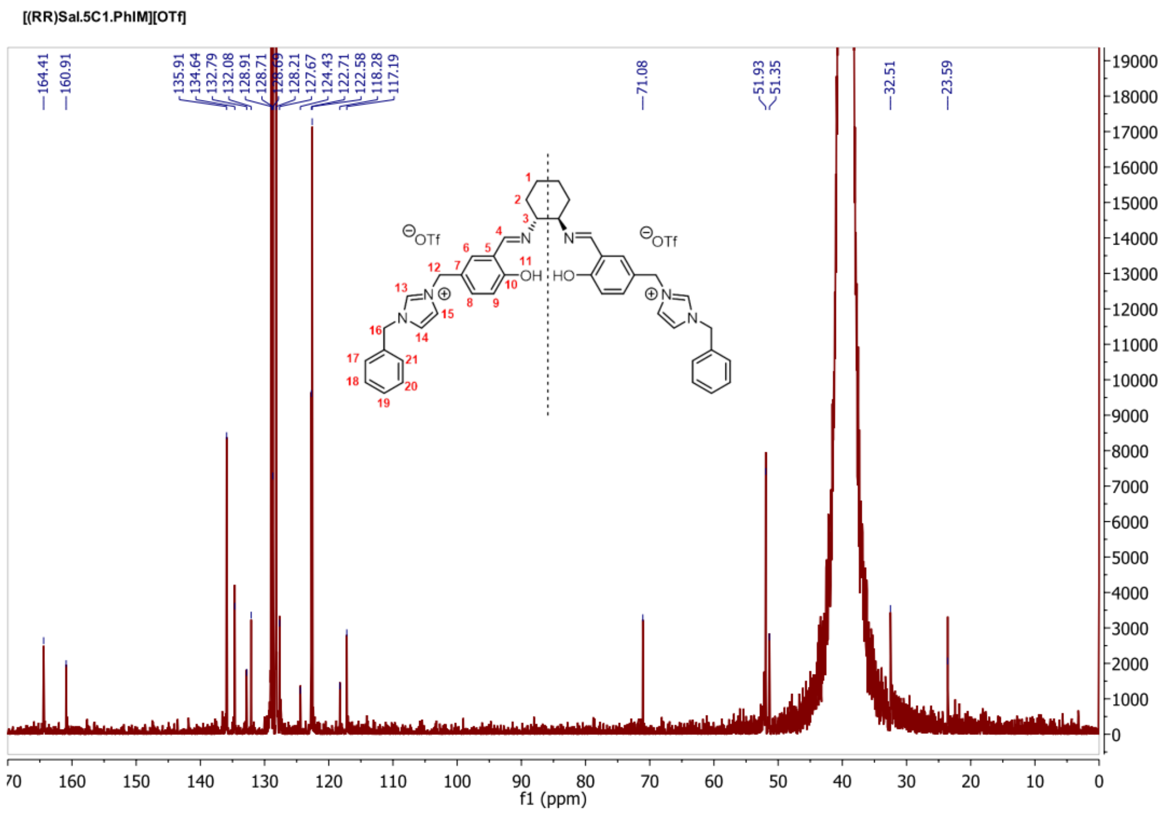

N,N′-bis-[5-((1-benzylimidazol-3-ium)methylene)-salicylidene]-*trans*-(1*R*,2*R*)-cyclohexanediamine di[bis(trifluoromethanesulfonyl)imide], **[(RR)Sal.5C1.PhIM][NTf₂]** in DMSO-d6

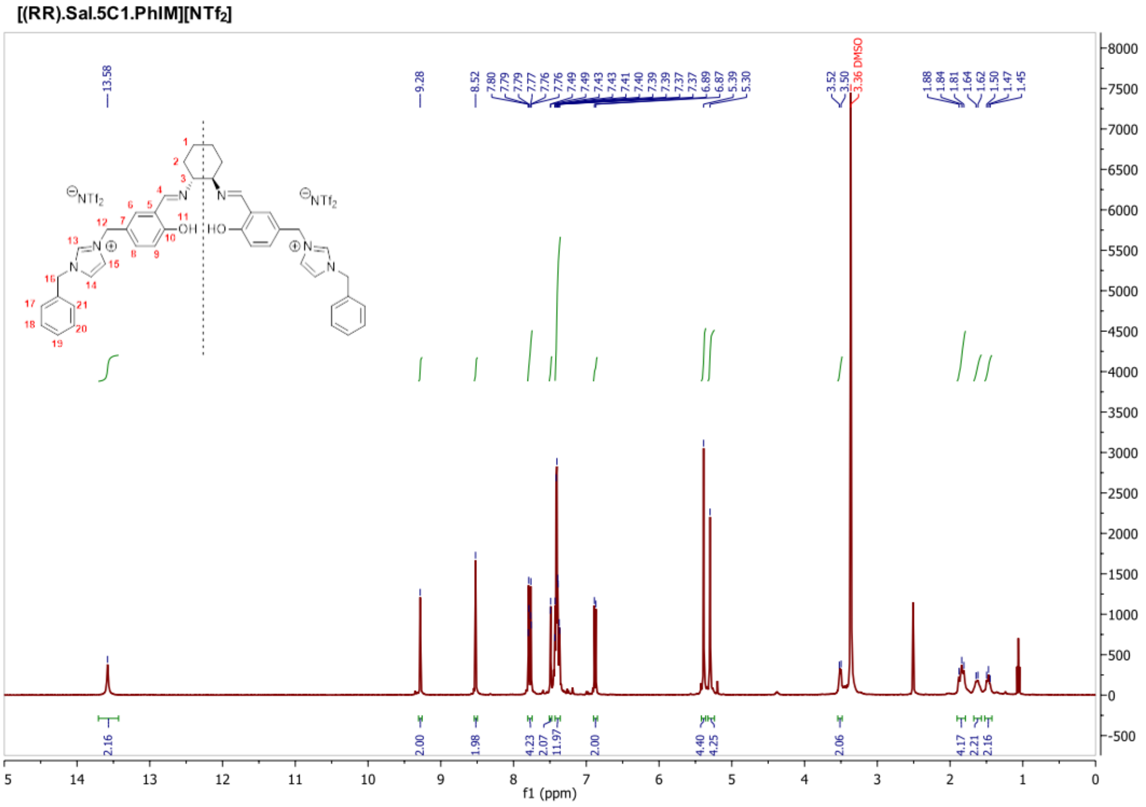

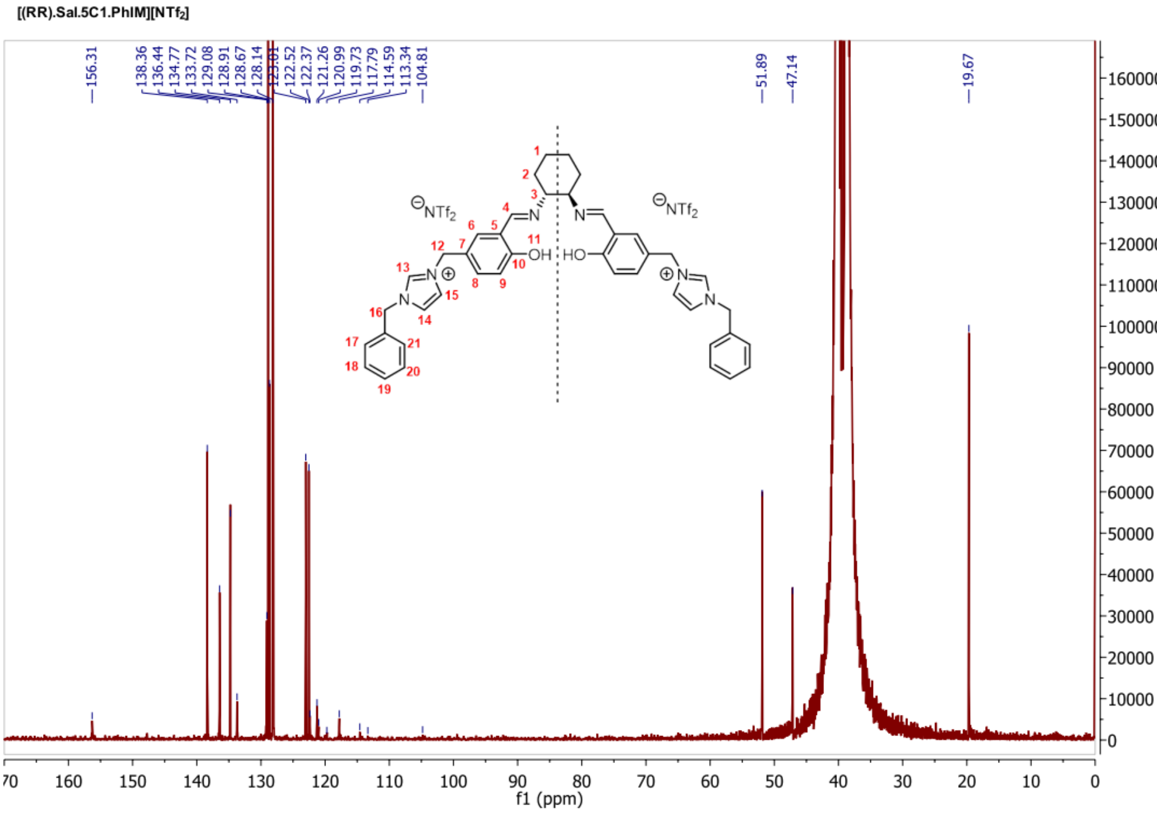

N,N′-bis-[5-((pyridinium)methylene)]-salicylidene]-*trans*-(1*R*,2*R*)-cyclohexanediamine dichloride, **[(RR)Sal.5C1.Pyr][Cl]** in DMSO-d6

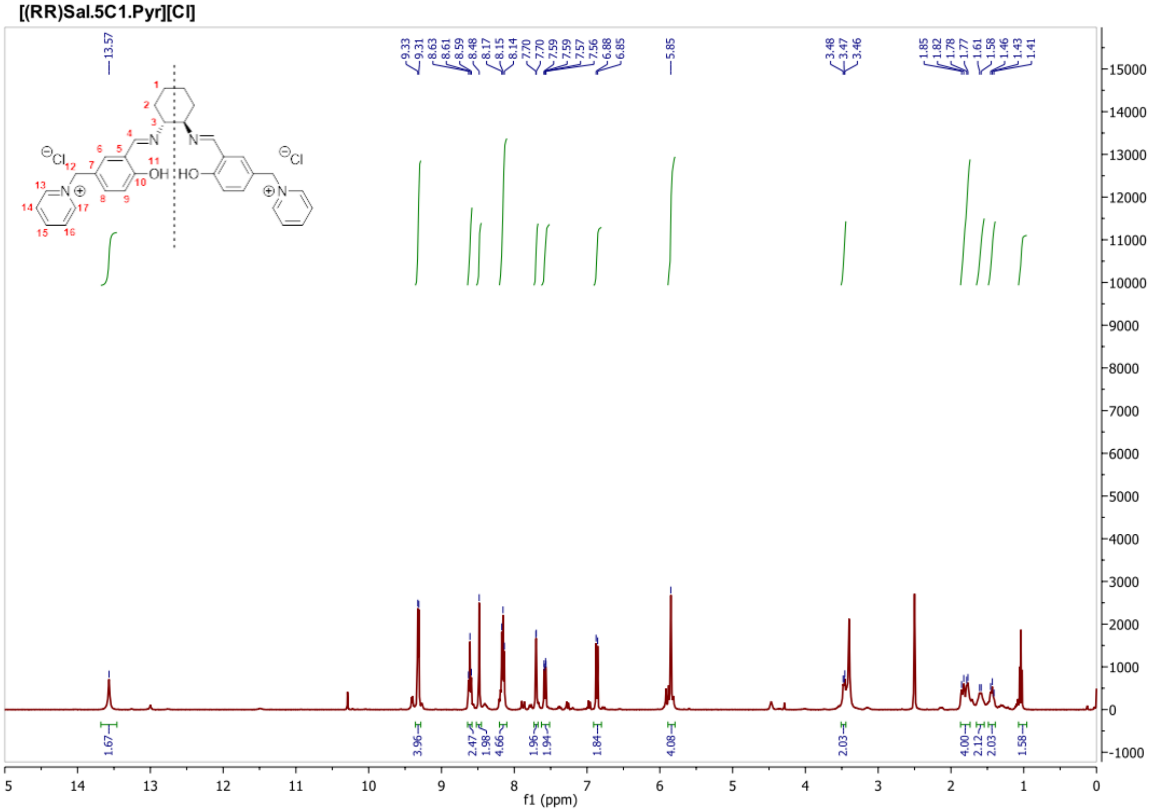

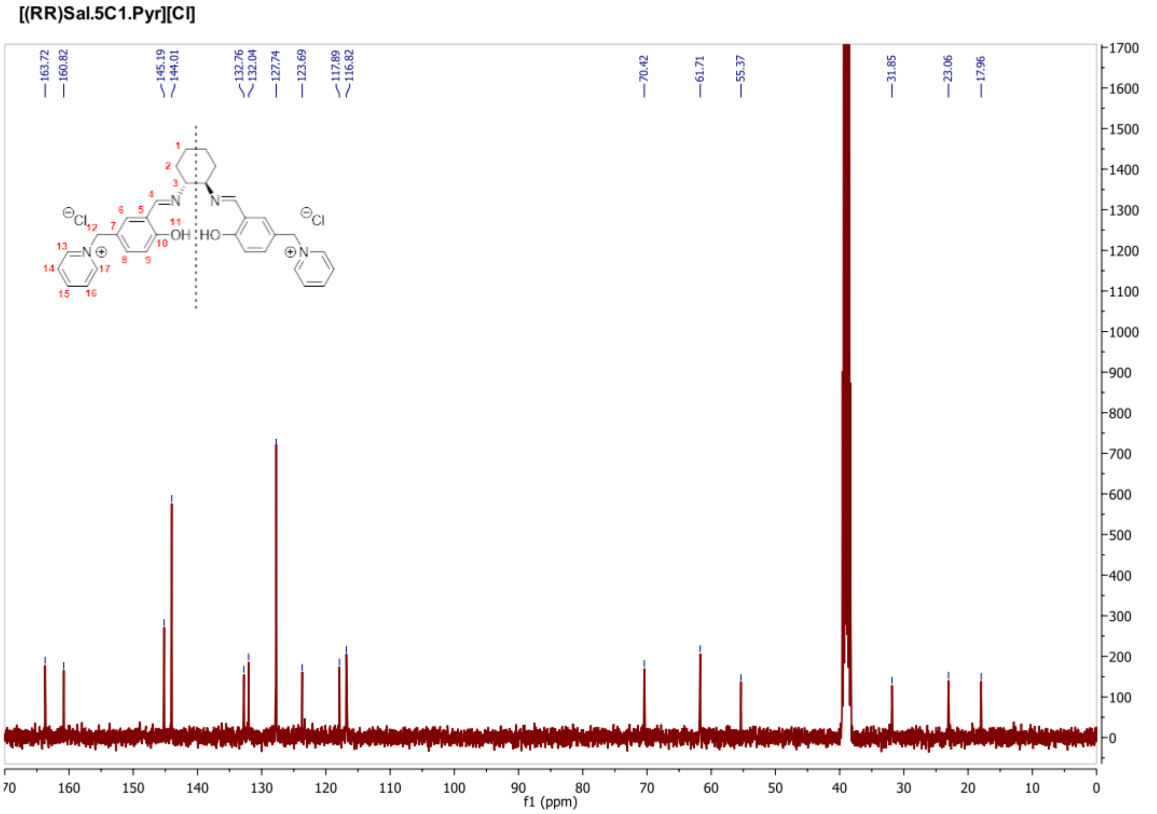

N,N′-bis-[5-((pyridinium)methylene)]-salicylidene]-*trans*-(1*R*,2*R*)-cyclohexanediamine ditetrafluoroborate, **[(RR)Sal.5C1.Pyr][BF$_4$]** in DMSO-d6

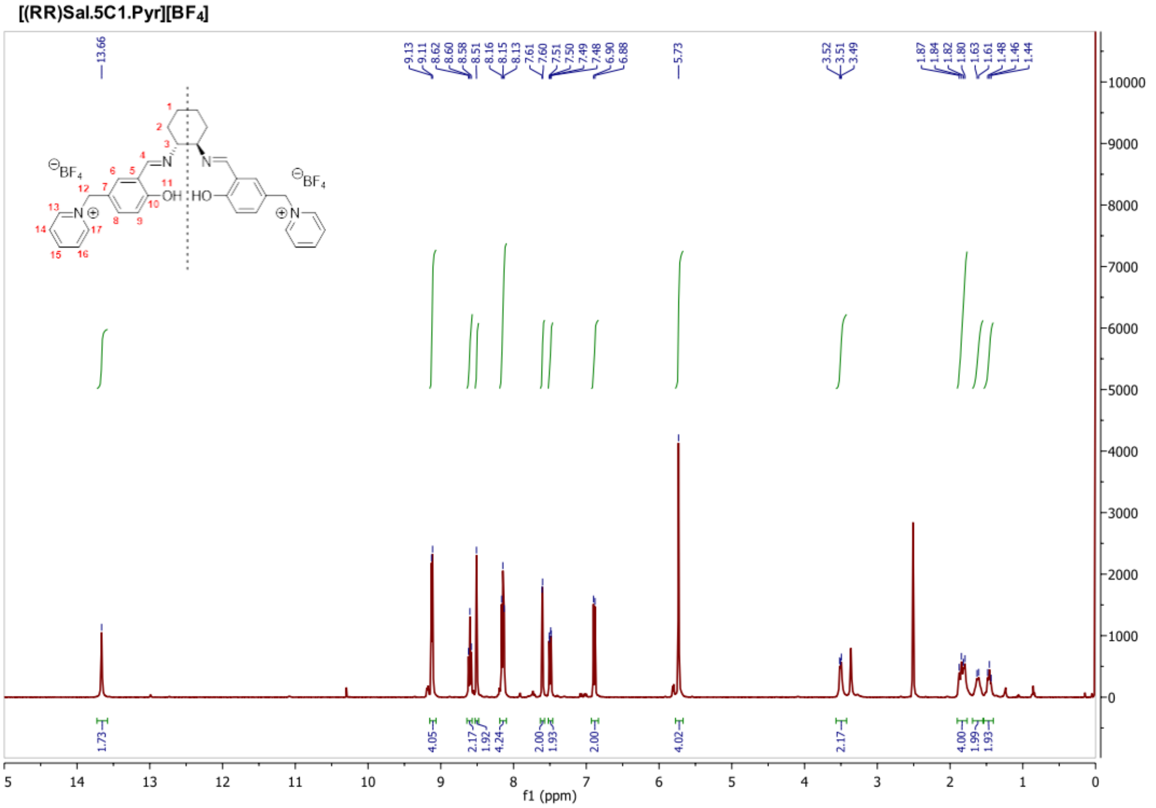

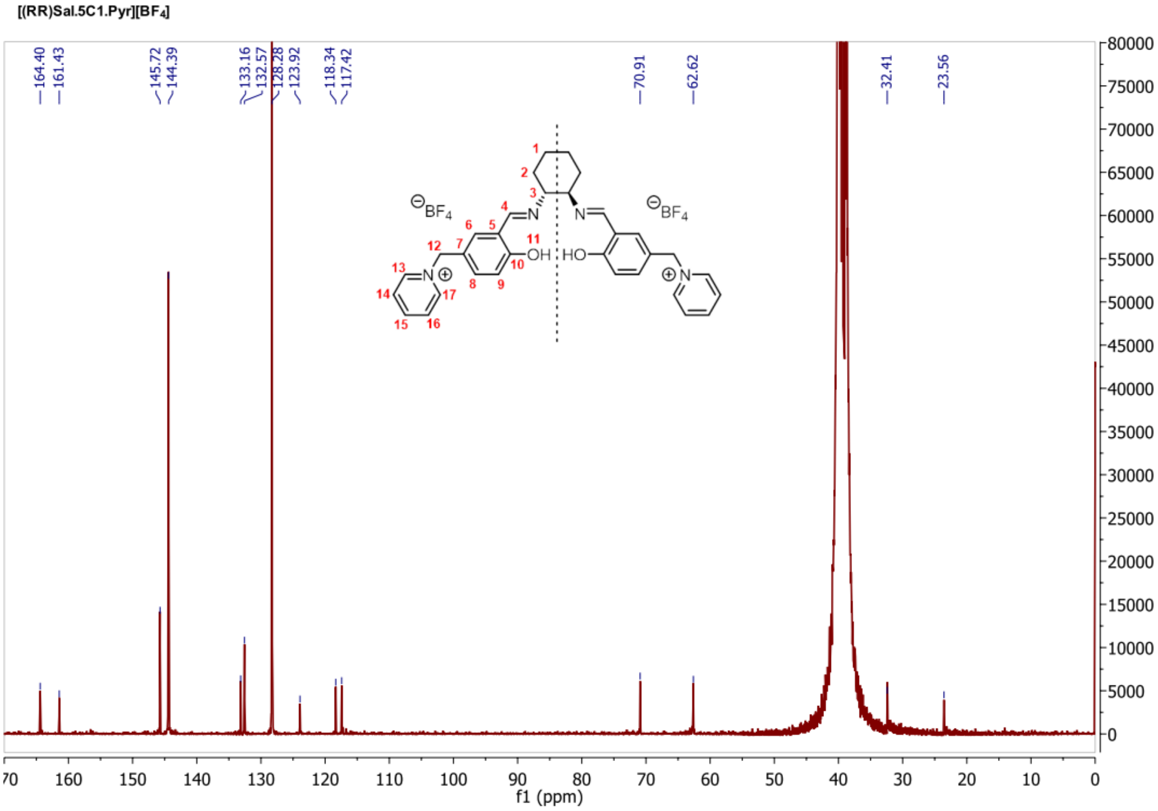

N,N′-bis-[5-((pyridinium)methylene)]-salicylidene]-*trans*-(1*R*,2*R*)-cyclohexanediamine ditrifluoromethanesulfonate, **[(RR)Sal.5C1.Pyr][OTf]** in DMSO-d6

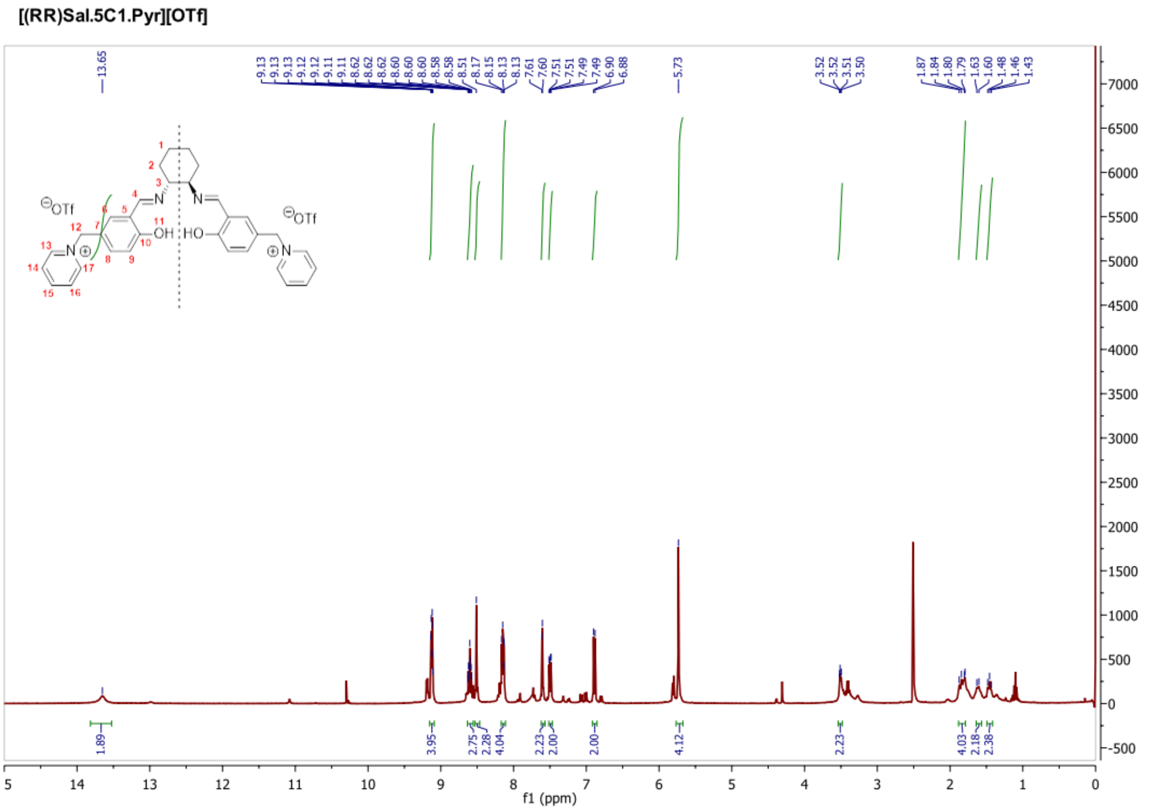

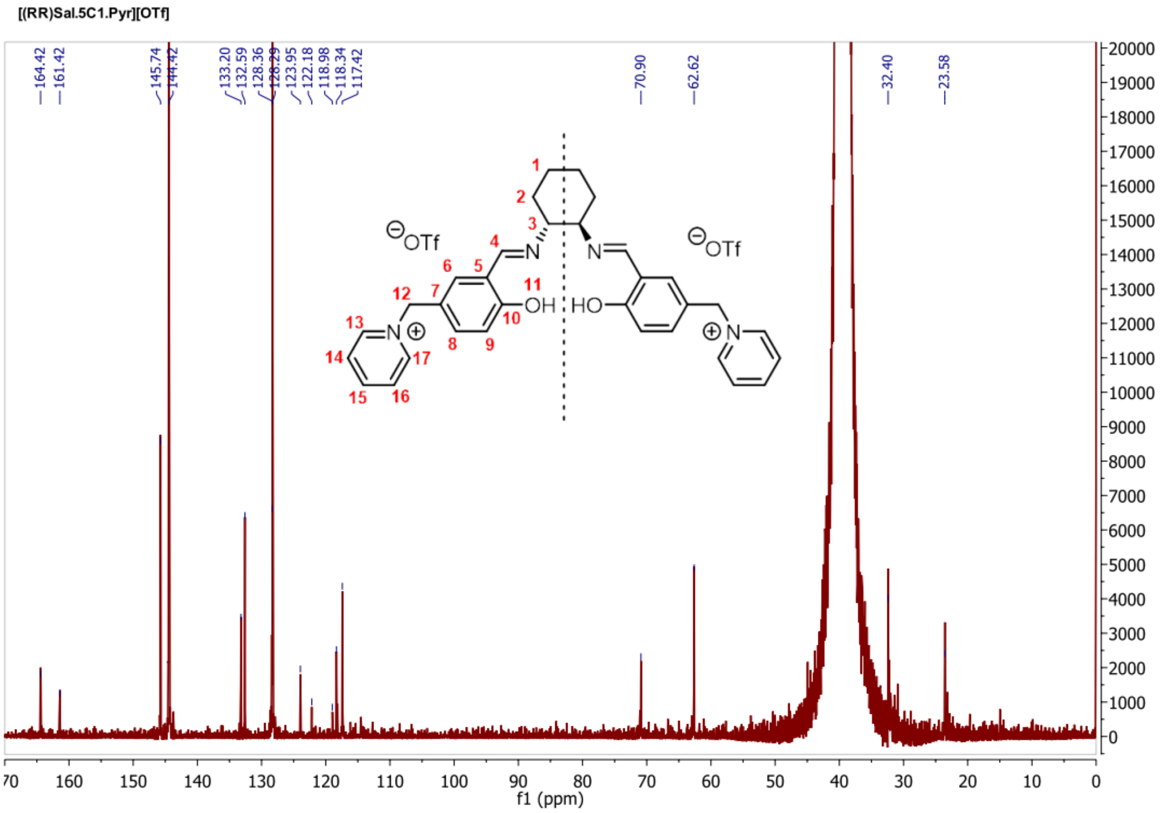

N,N′-bis-[5-((pyridinium)methylene)-salicylidene]-*trans*-(1*R*,2*R*)-cyclohexanediamine di[bis(trifluoromethanesulfonyl)imide], **[(RR)Sal.5C1.Pyr][NTf$_2$]** in DMSO-d6

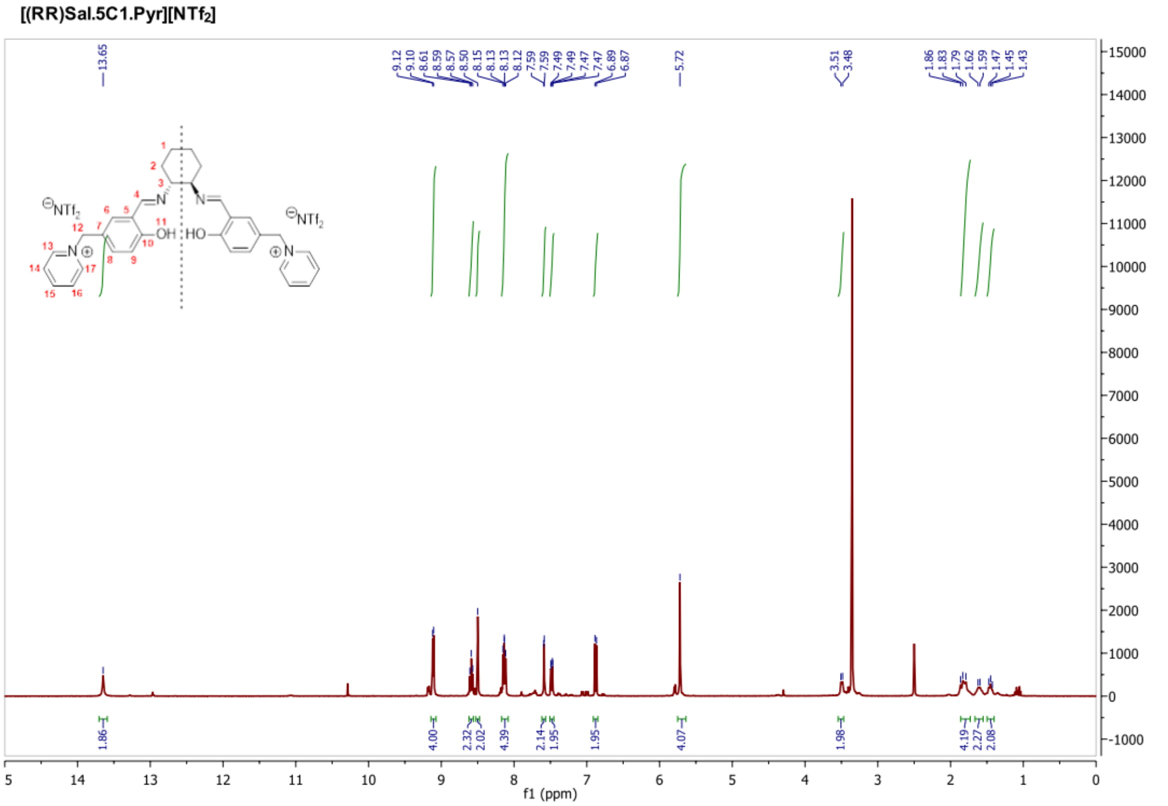

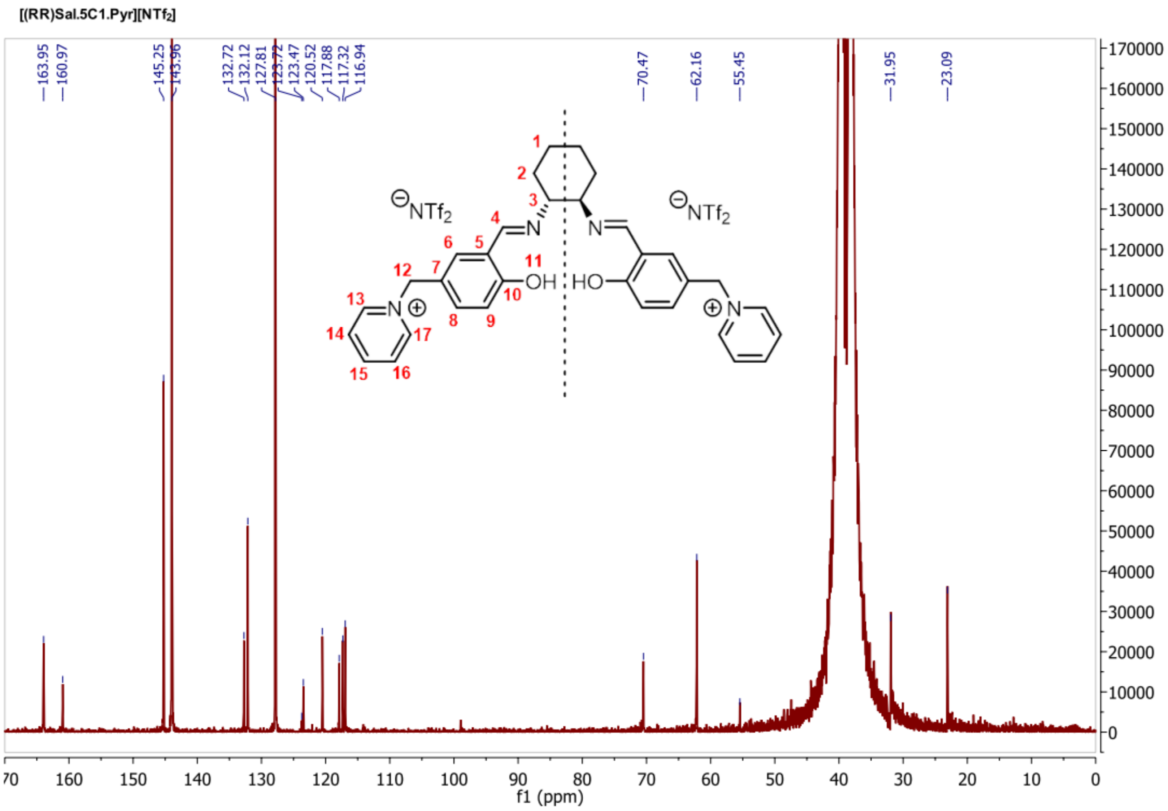

2. Copies of FTIR spectra of chiral salen organic salts.

N,N′-bis-[5-((1-methylimidazol-3-ium)methylene)-salicylidene]-*trans*-(1*R*,2*R*)-cyclohexanediamine dichloride, **[(RR)Sal.5C1.MIM][Cl]**

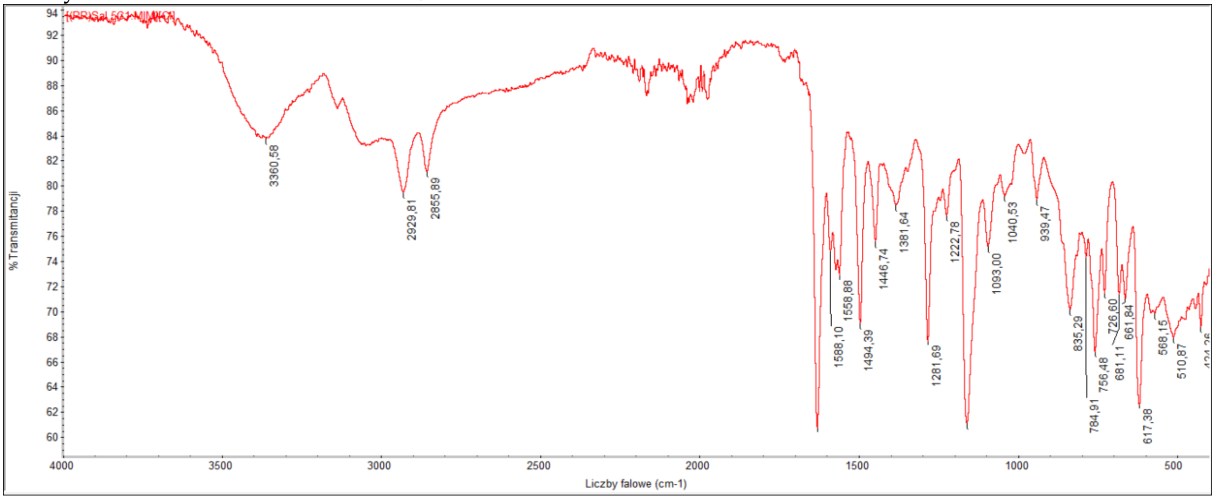

N,N′-bis-[5-((1-methylimidazol-3-ium)methylene)-salicylidene]-*trans*-(±)-cyclohexanediamine dichloride, **[(rac)Sal.5C1.MIM][Cl]**

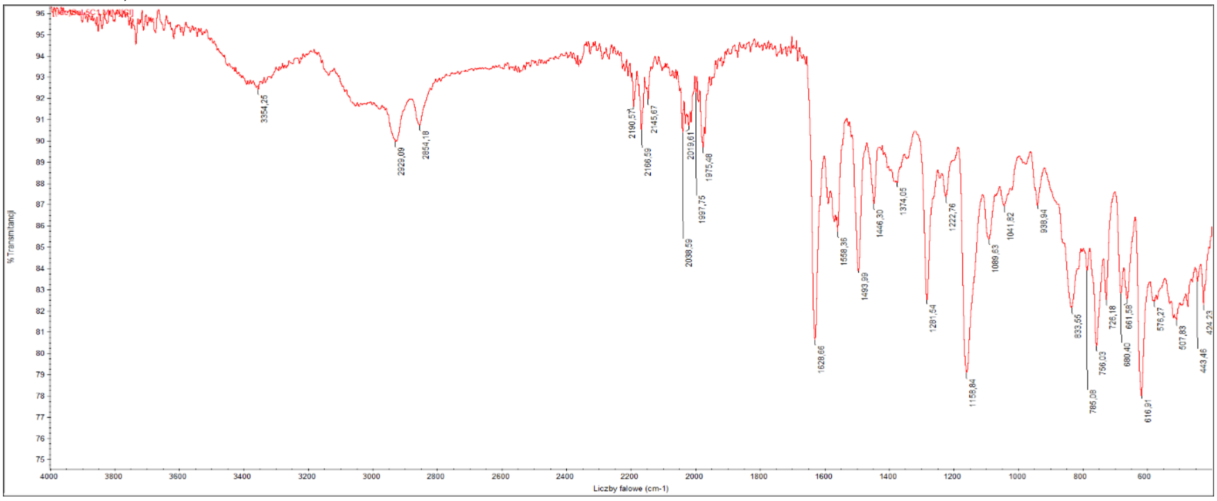

N,N′-bis-[5-((1-methylimidazol-3-ium)methylene)-salicylidene]-*trans*-(1*R*,2*R*)-cyclohexanediamine ditetrafluoroborate, **[(RR)Sal.5C1.MIM][BF$_4$]**

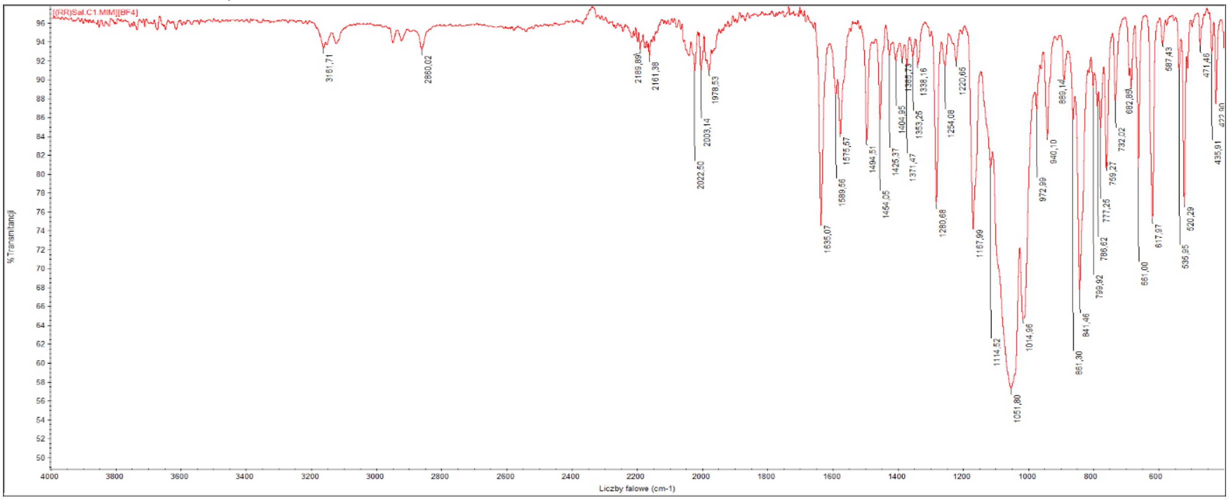

N,N′-bis-[5-((1-methylimidazol-3-ium)methylene)-salicylidene]-*trans*-(1*R*,2*R*)-cyclohexanediamine ditrifluoromethanesulfonate, **[(RR)Sal.5C1.MIM][OTf]**

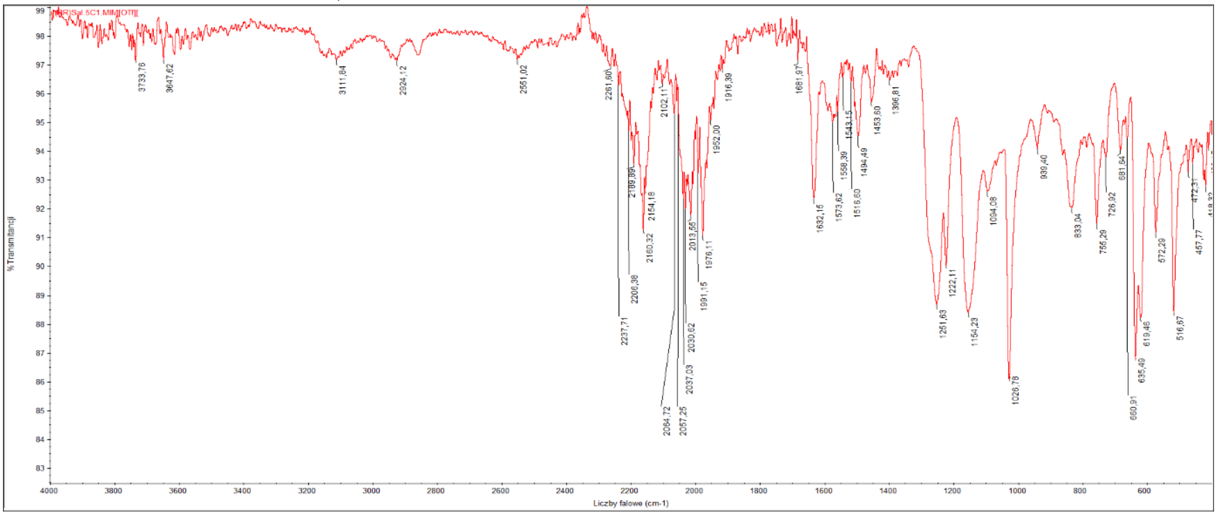

N,N′-bis-[5-((1-methylimidazol-3-ium)methylene)-salicylidene]-*trans*-(1*R*,2*R*)-cyclohexanediamine di[bis(trifluoromethanesulfonyl)imide], **[(RR)Sal.5C1.MIM][NTf$_2$]**

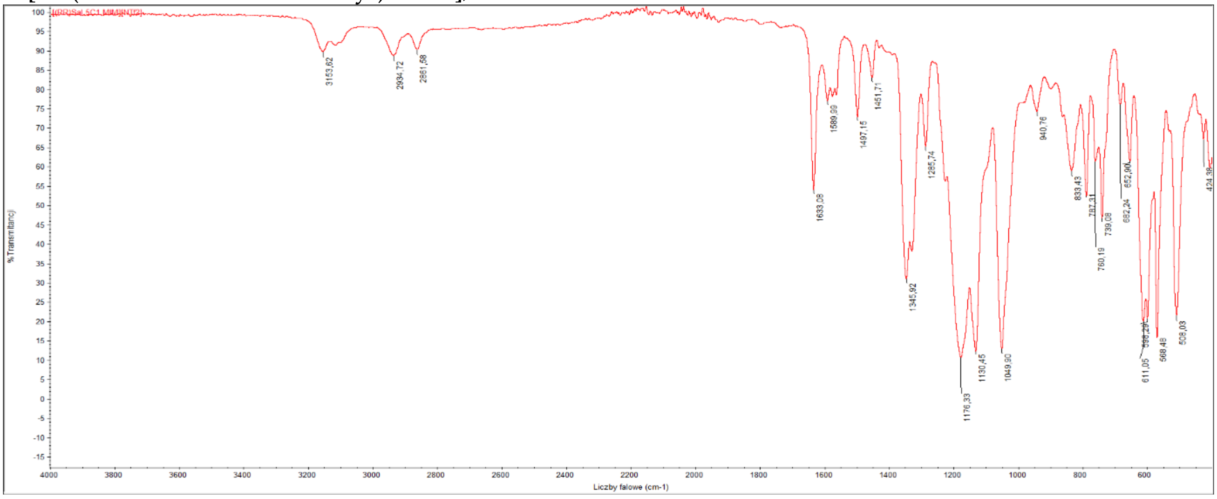

N,N′-bis-[5-((1-benzylimidazol-3-ium)methylene)-salicylidene]-*trans*-(1*R*,2*R*)-cyclohexanediamine dichloride **[(RR)Sal.5C1.PhIM][Cl]**

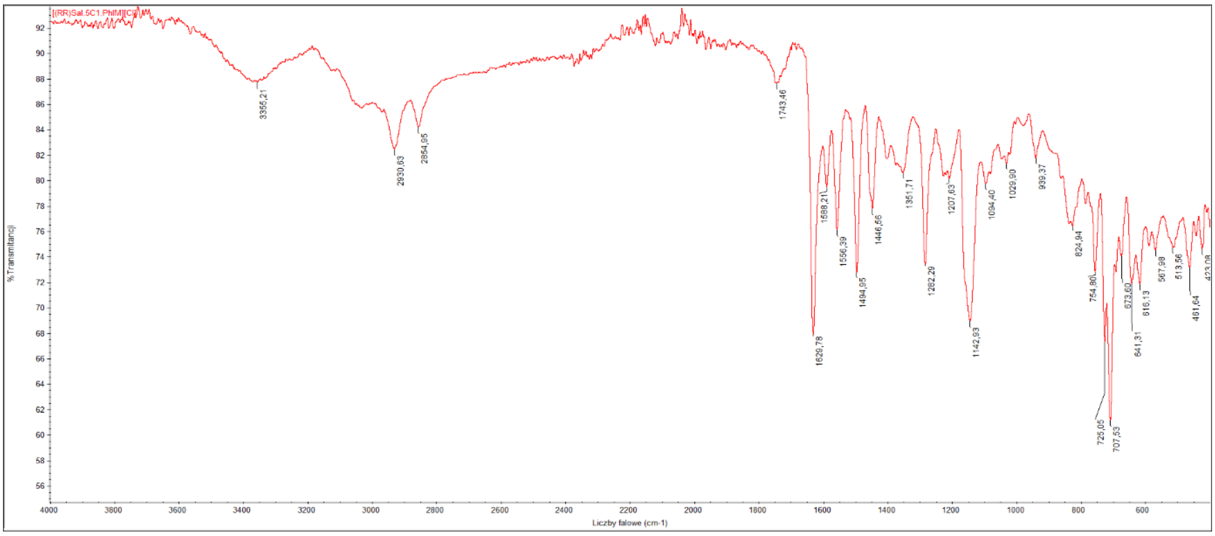

N,N'-bis-[5-((1-benzylimidazol-3-ium)methylene)-salicylidene]-*trans*-(1*R*,2*R*)-cyclohexanediamine ditetrafluoroborate, **[(RR)Sal.5C1.PhIM][BF$_4$]**

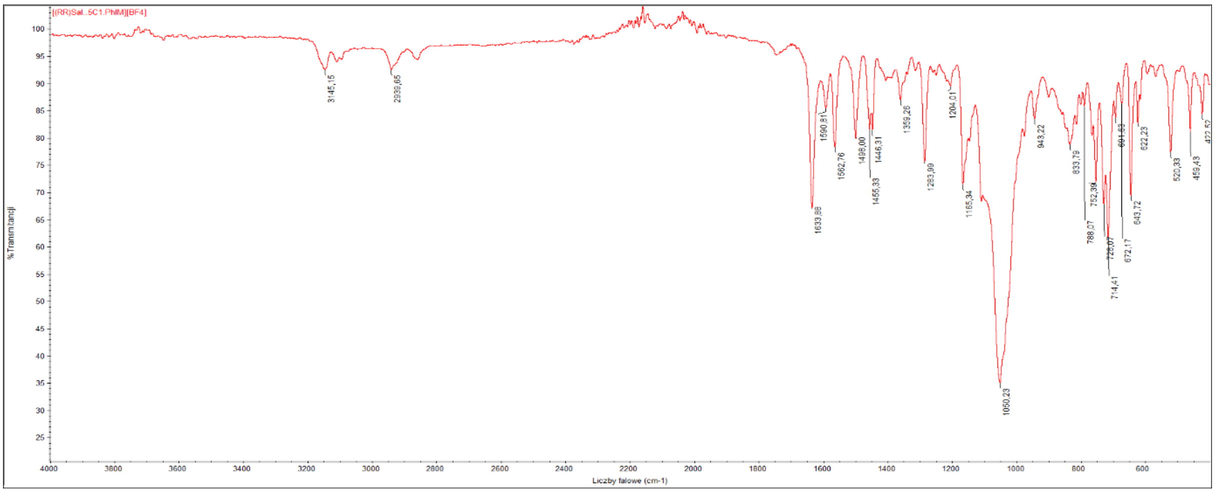

N,N'-bis-[5-((1-benzylimidazol-3-ium)methylene)-salicylidene]-*trans*-(1*R*,2*R*)-cyclohexanediamine ditrifluoromethanesulfonate, **[(RR)Sal.5C1.PhIM][OTf]**

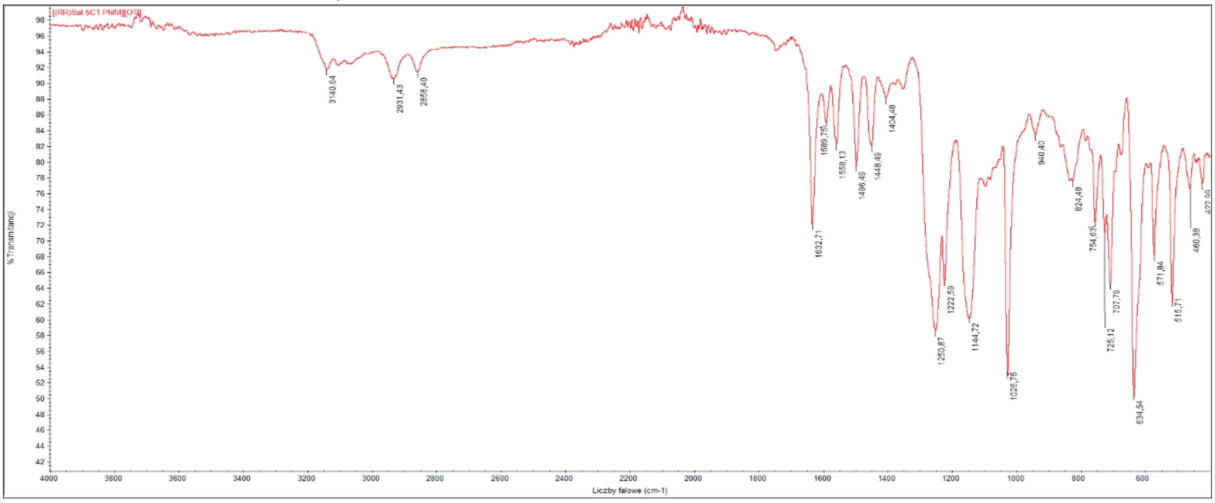

N,N'-bis-[5-((1-benzylimidazol-3-ium)methylene)-salicylidene]-*trans*-(1*R*,2*R*)-cyclohexanediamine di[bis(trifluoromethanesulfonyl)imide], **[(RR)Sal.5C1.PhIM][NTf$_2$]**

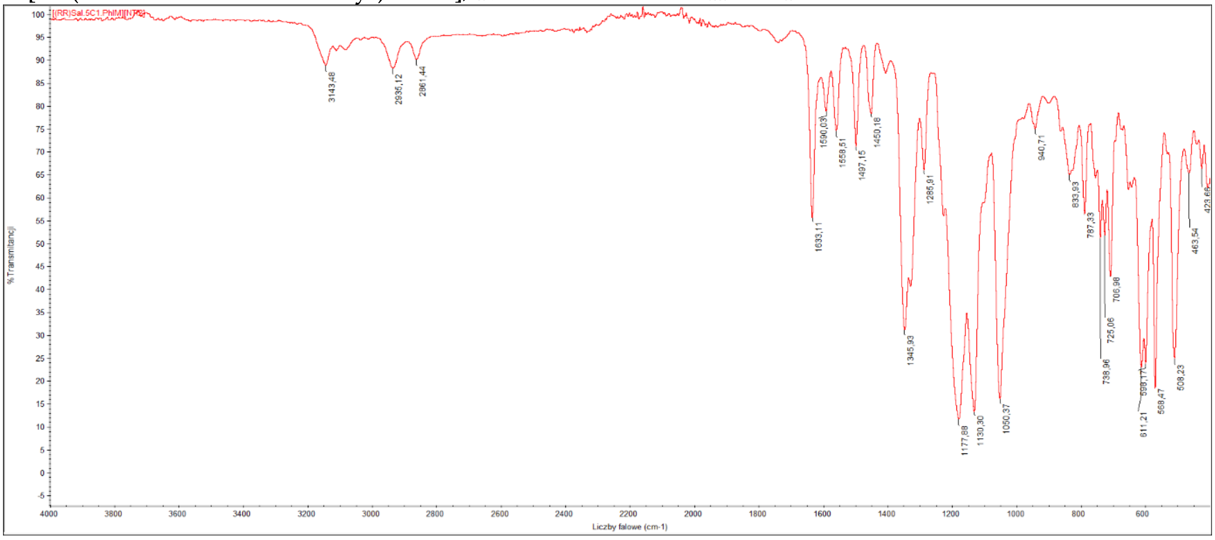

N,N'-bis-[5-((pyridinium)methylene)-salicylidene]-*trans*-(1*R*,2*R*)-cyclohexanediamine dichloride, **[(RR)Sal.5C1.Pyr][Cl]**

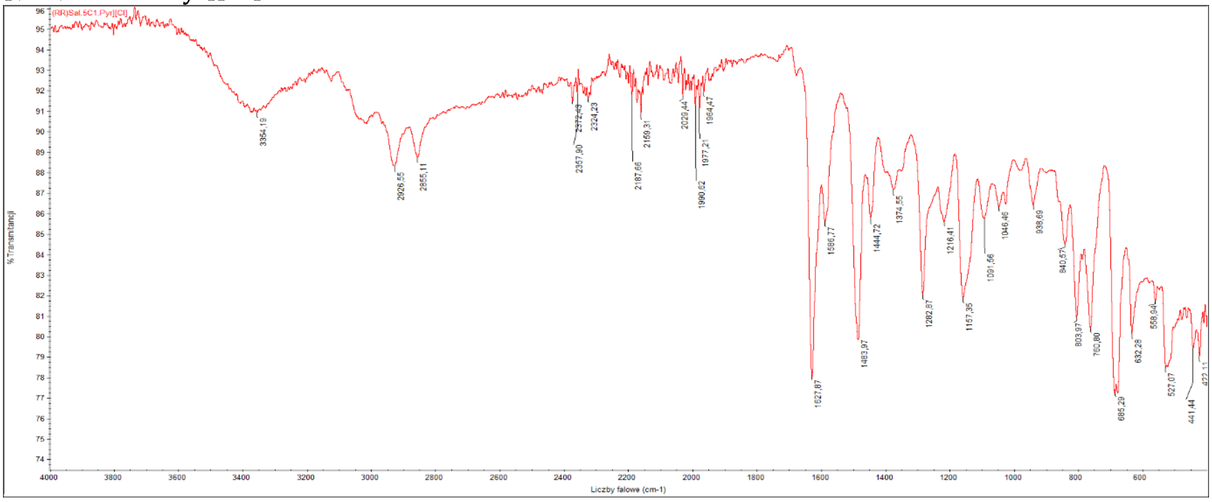

N,N'-bis-[5-((pyridinium)methylene)-salicylidene]-*trans*-(1*R*,2*R*)-cyclohexanediamine ditetrafluoroborate, **[(RR)Sal.5C1.Pyr][BF_4]**

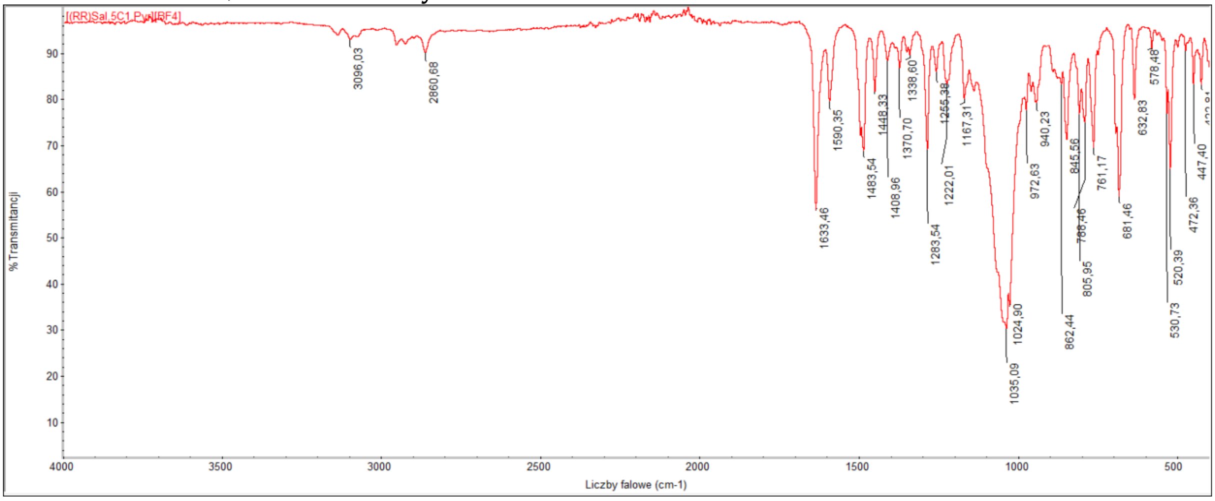

N,N'-bis-[5-((pyridinium)methylene)-salicylidene]-*trans*-(1*R*,2*R*)-cyclohexanediamine ditrifluoromethanesulfonate, **[(RR)Sal.5C1.Pyr][OTf]**

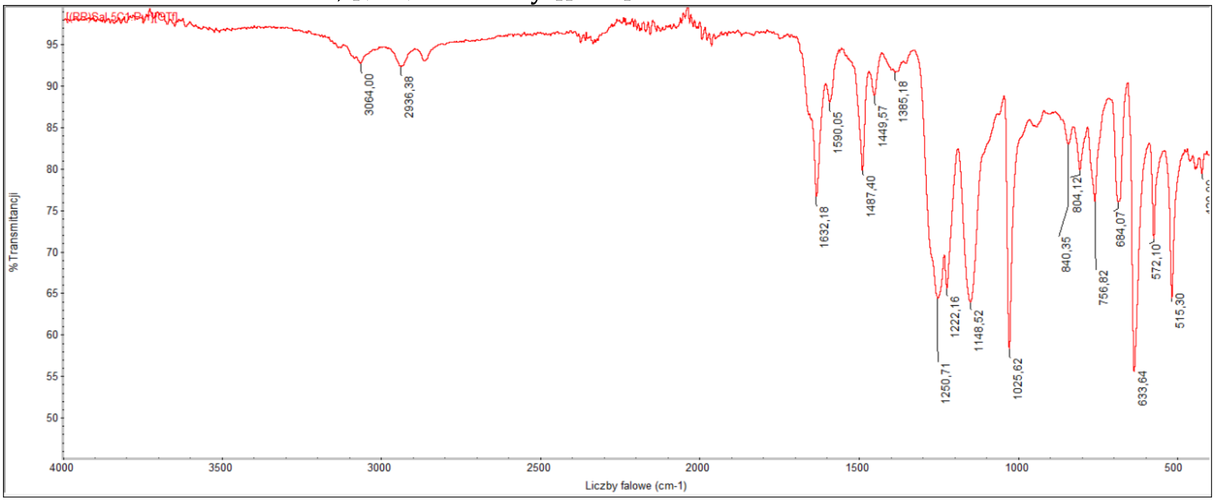

N,N′-bis-[5-((pyridinium)methylene)-salicylidene]-*trans*-(1*R*,2*R*)-cyclohexanediamine di[bis(trifluoromethanesulfonyl)imide], **[(RR)Sal.5C1.Pyr][NTf₂]**

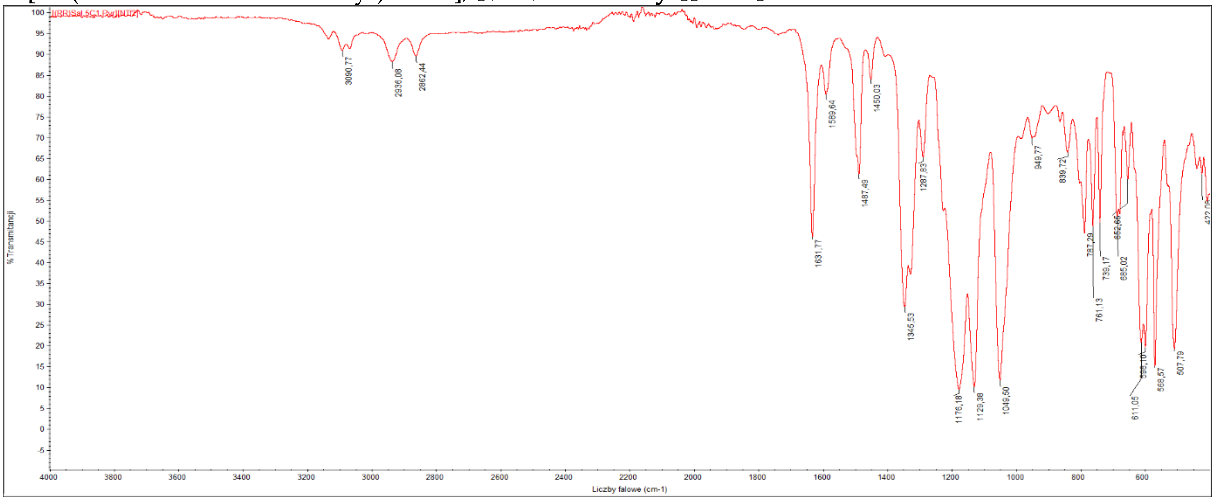

3. Curves from thermogravimetric analysis chiral salen organic salts.

N,N'-bis-[5-((1-methylimidazol-3-ium)methylene)-salicylidene]-*trans*-(1*R*,2*R*)-cyclohexanediamine dichloride, **[(RR)Sal.5C1.MIM][Cl]**

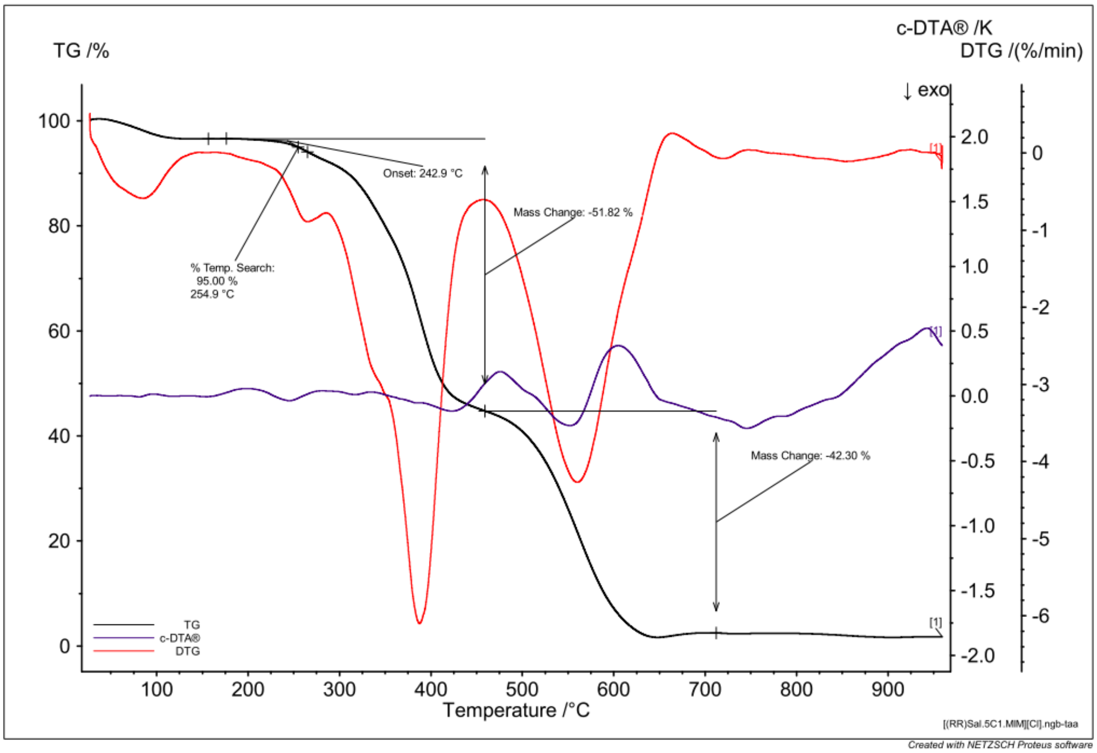

N,N'-bis-[5-((1-methylimidazol-3-ium)methylene)-salicylidene]-*trans*-(±)-cyclohexanediamine dichloride, **[(rac)Sal.5C1.MIM][Cl]**

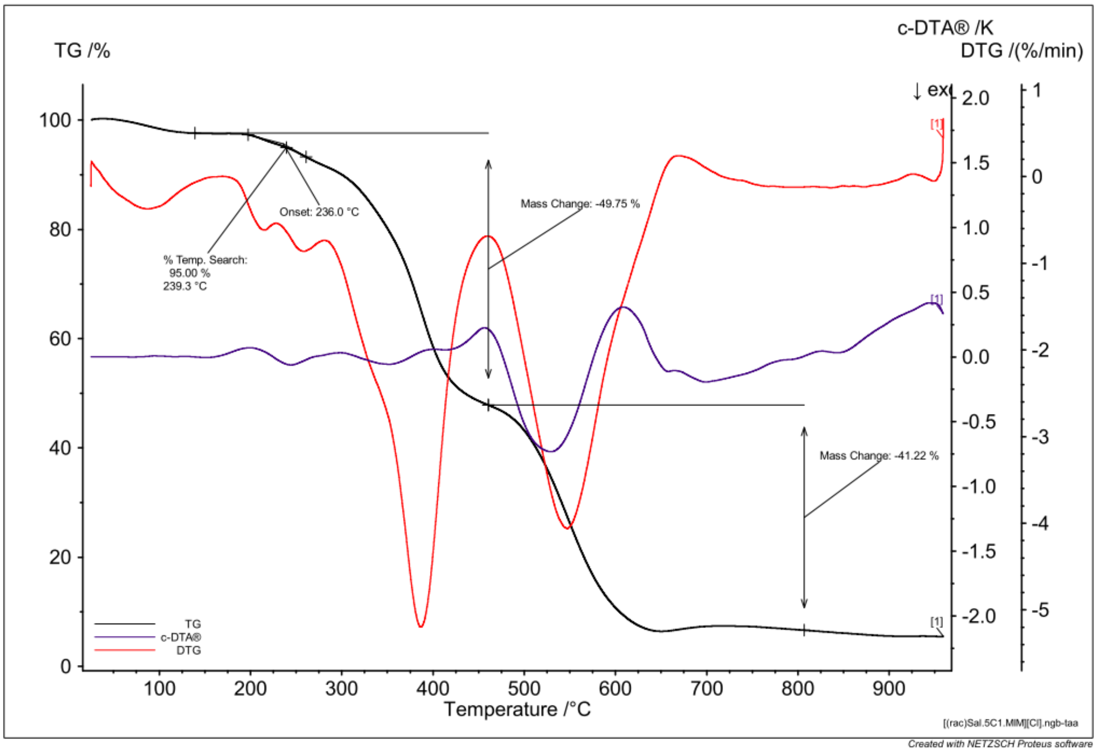

N,*N′*-bis-[5-((1-methylimidazol-3-ium)methylene)-salicylidene]-*trans*-(1*R*,2*R*)-cyclohexanediamine ditetrafluoroborate, **[(RR)Sal.5C1.MIM][BF$_4$]**

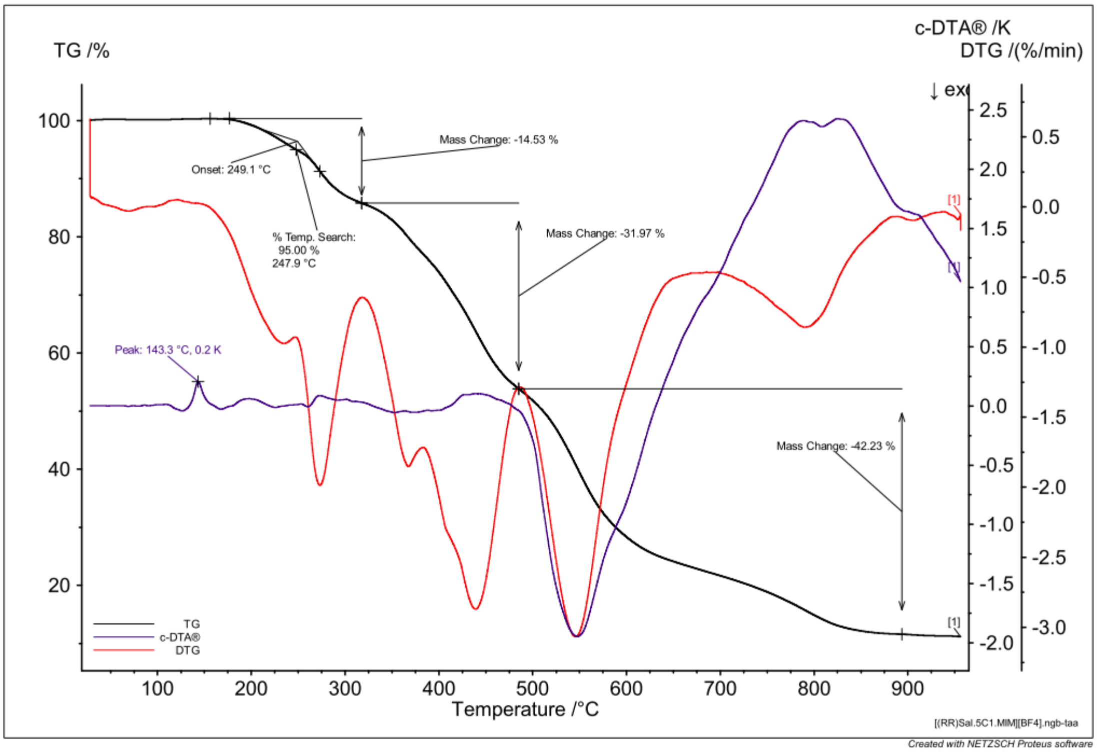

N,*N′*-bis-[5-((1-methylimidazol-3-ium)methylene)-salicylidene]-*trans*-(1*R*,2*R*)-cyclohexanediamine ditrifluoromethanesulfonate, **[(RR)Sal.5C1.MIM][OTf]**

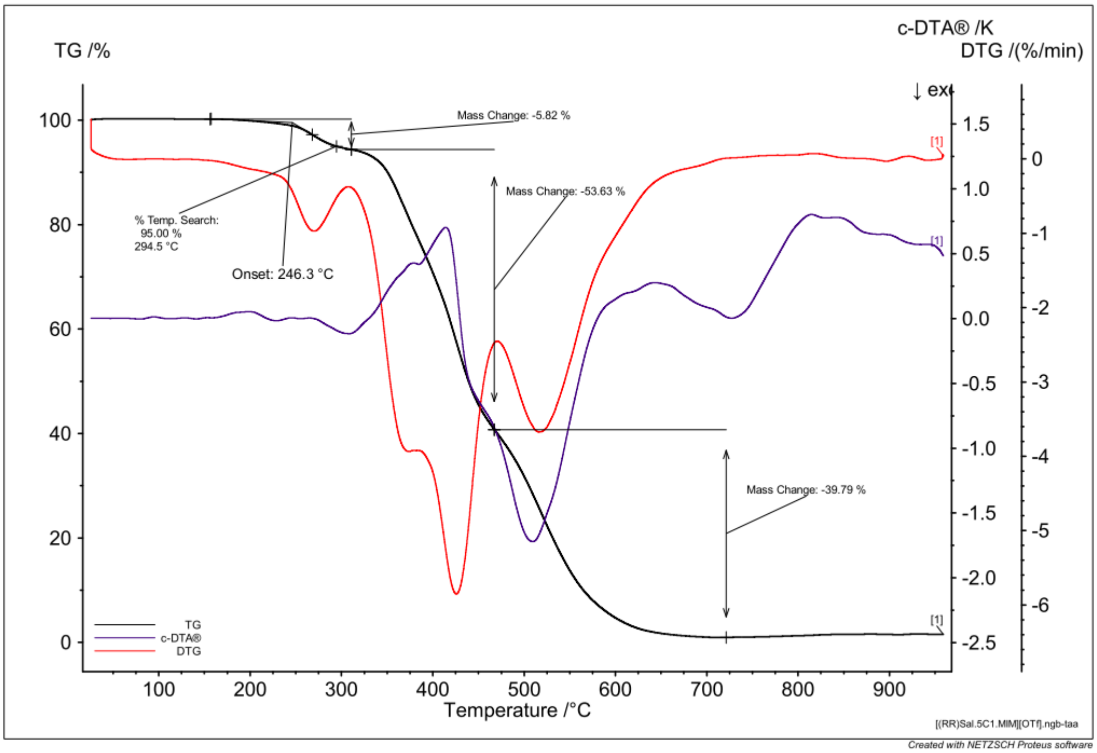

N,N′-bis-[5-((1-methylimidazol-3-ium)methylene)-salicylidene]-*trans*-(1*R*,2*R*)-cyclohexanediamine di[bis(trifluoromethanesulfonyl)imide], **[(RR)Sal.5C1.MIM][NTf$_2$]**

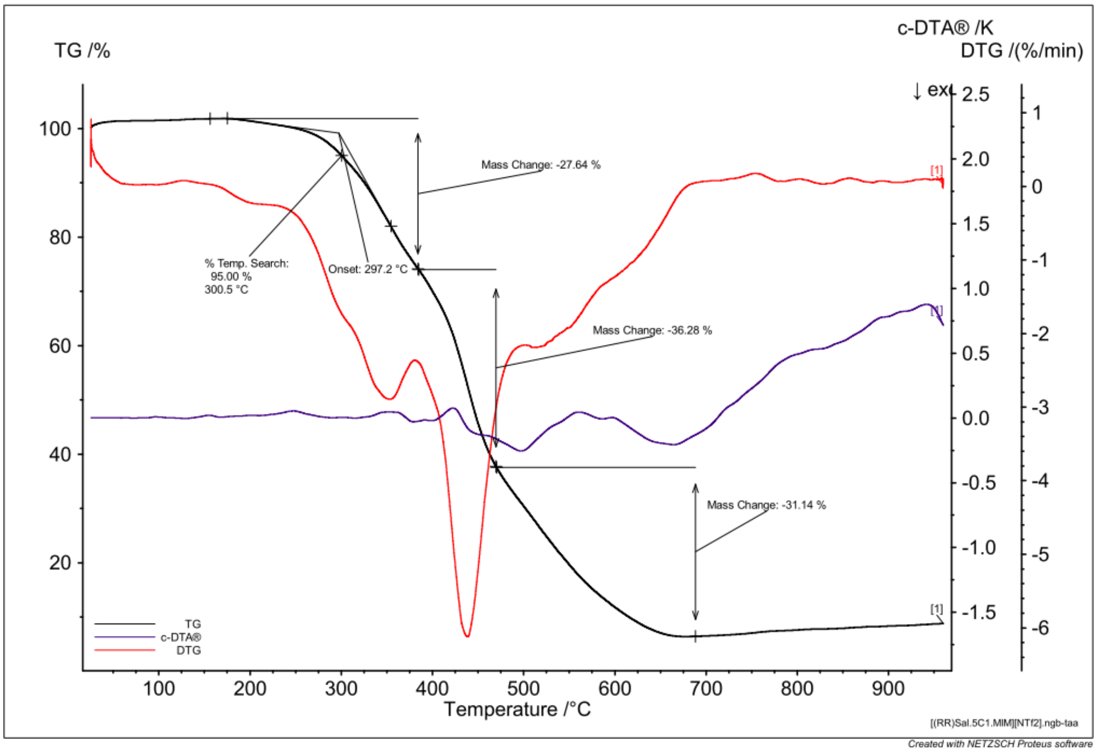

N,N′-bis-[5-((1-benzylimidazol-3-ium)methylene)-salicylidene]-*trans*-(1*R*,2*R*)-cyclohexanediamine dichloride, **[(RR)Sal.5C1.PhIM][Cl]**

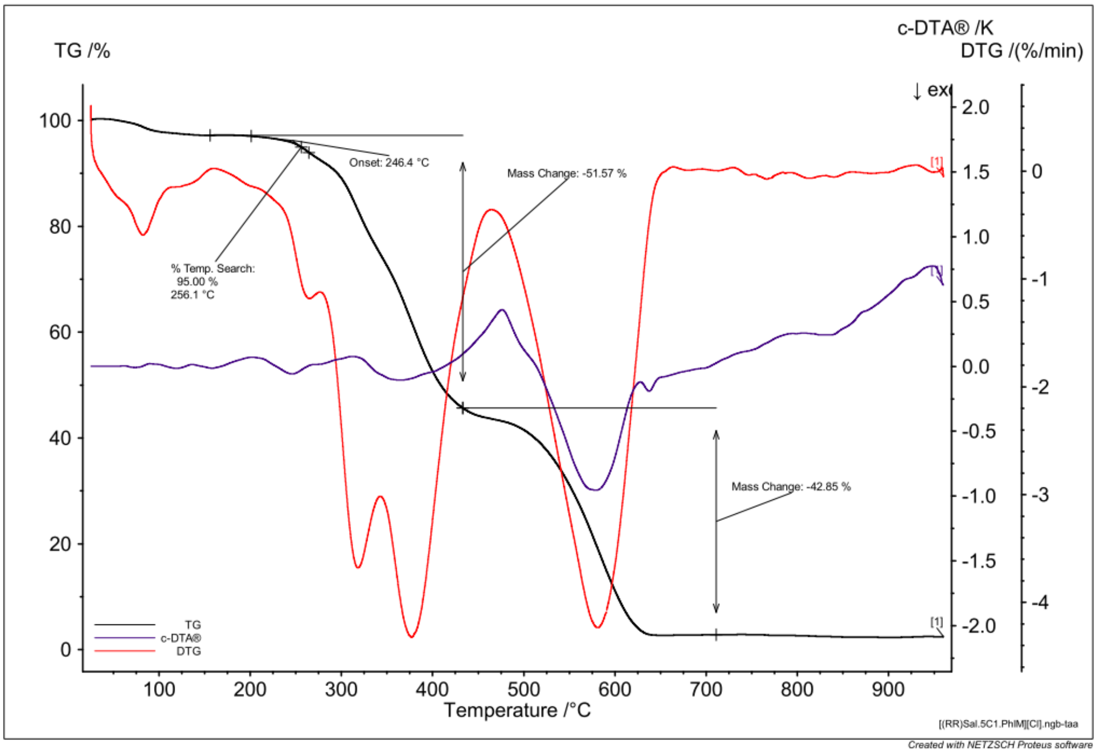

N,N'-bis-[5-((1-benzylimidazol-3-ium)methylene)-salicylidene]-*trans*-(1*R*,2*R*)-cyclohexanediamine ditetrafluoroborate, **[(RR)Sal.5C1.PhIM][BF$_4$]**

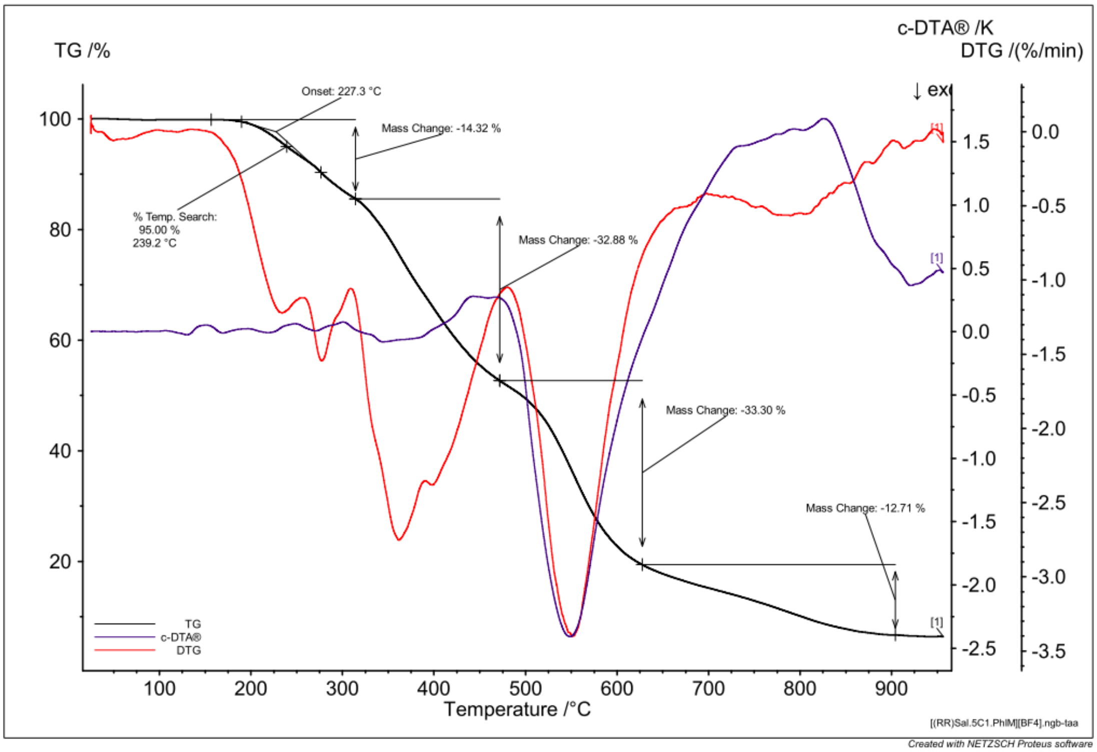

N,N'-bis-[5-((1-benzylimidazol-3-ium)methylene)-salicylidene]-*trans*-(1*R*,2*R*)-cyclohexanediamine ditrifluoromethanesulfonate, **[(RR)Sal.5C1.PhIM][OTf]**

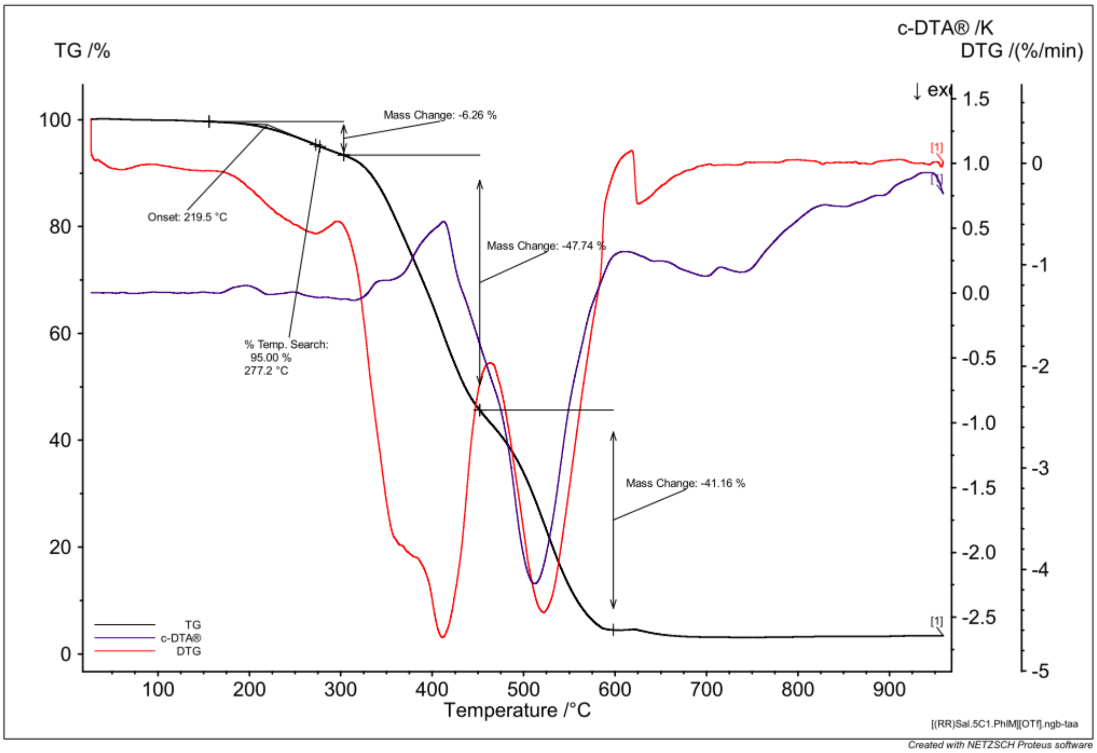

N,N′-bis-[5-((1-benzylimidazol-3-ium)methylene)-salicylidene]-*trans*-(1*R*,2*R*)-cyclohexanediamine di[bis(trifluoromethanesulfonyl)imide], **[(RR)Sal.5C1.PhIM][NTf₂]**

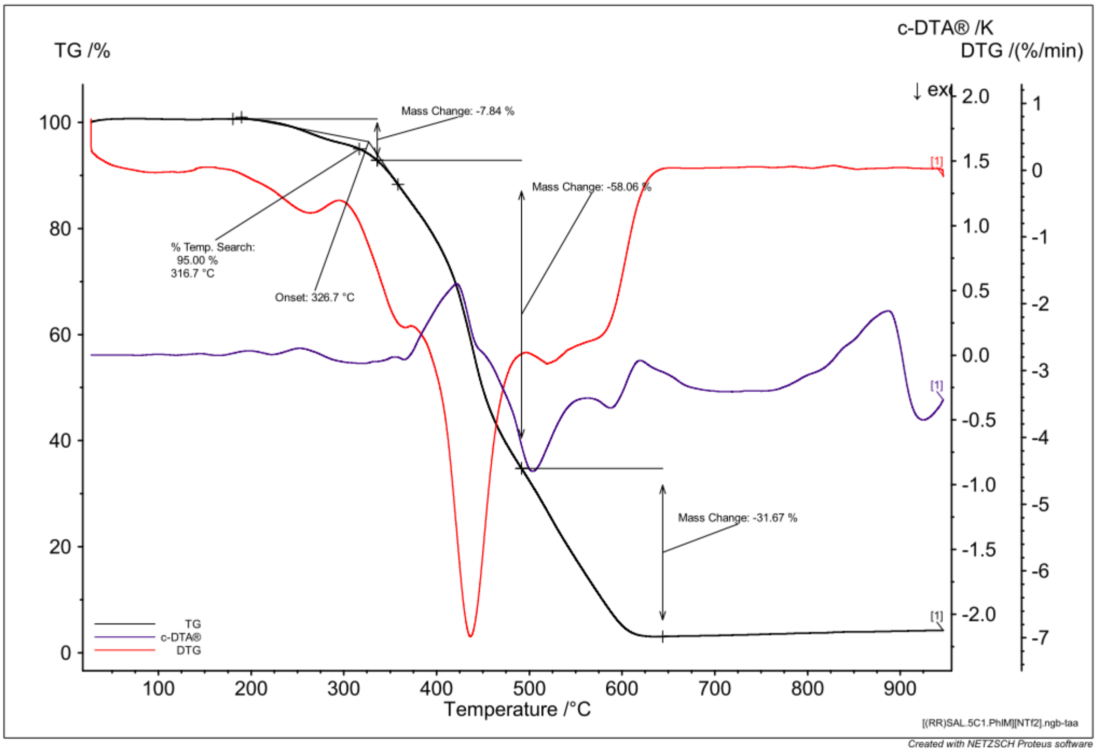

N,N′-bis-[5-((pyridinium)methylene)-salicylidene]-*trans*-(1*R*,2*R*)-cyclohexanediamine dichloride, **[(RR)Sal.5C1.Pyr][Cl]**

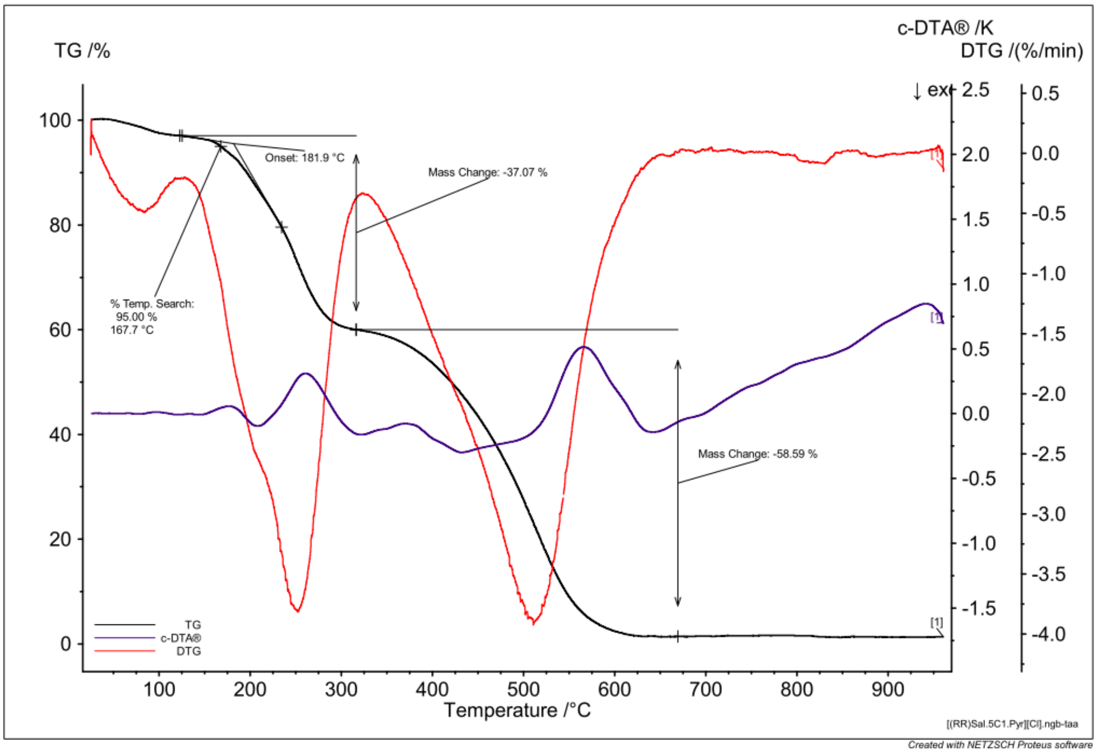

N,N′-bis-[5-((pyridinium)methylene)-salicylidene]-*trans*-(1*R*,2*R*)-cyclohexanediamine ditetrafluoroborate, **[(RR)Sal.5C1.Pyr][BF$_4$]**

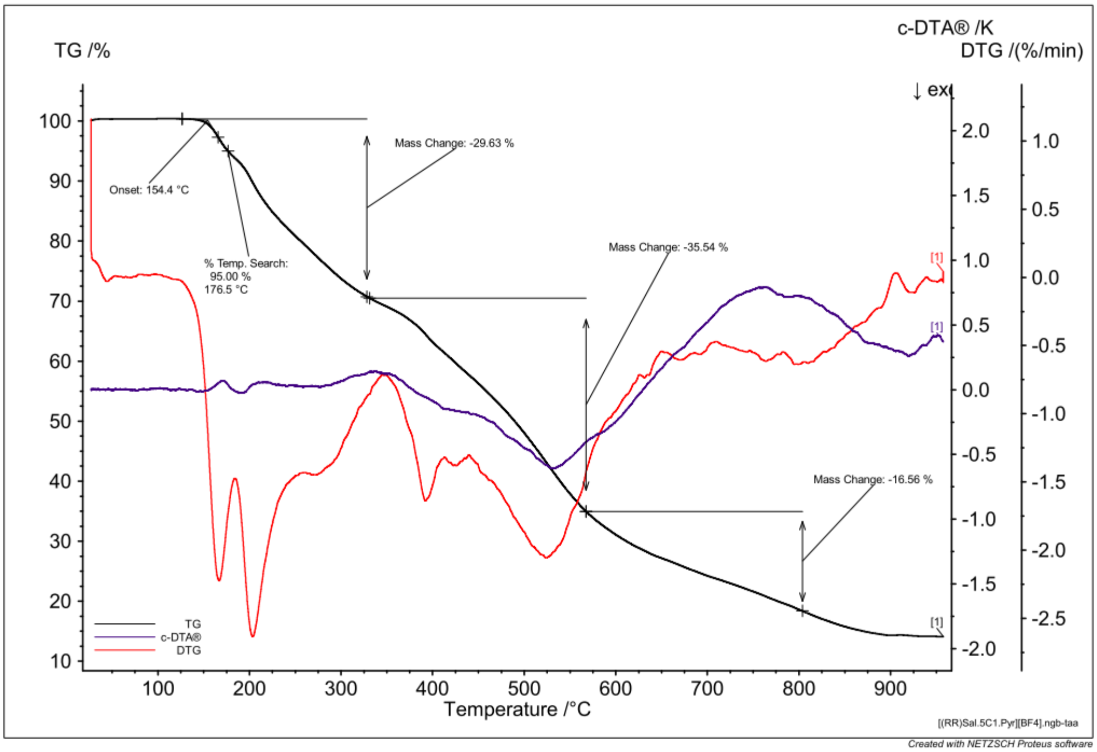

N,N′-bis-[5-((pyridinium)methylene)-salicylidene]-*trans*-(1*R*,2*R*)-cyclohexanediamine ditrifluoromethanesulfonate, **[(RR)Sal.5C1.Pyr][OTf]**

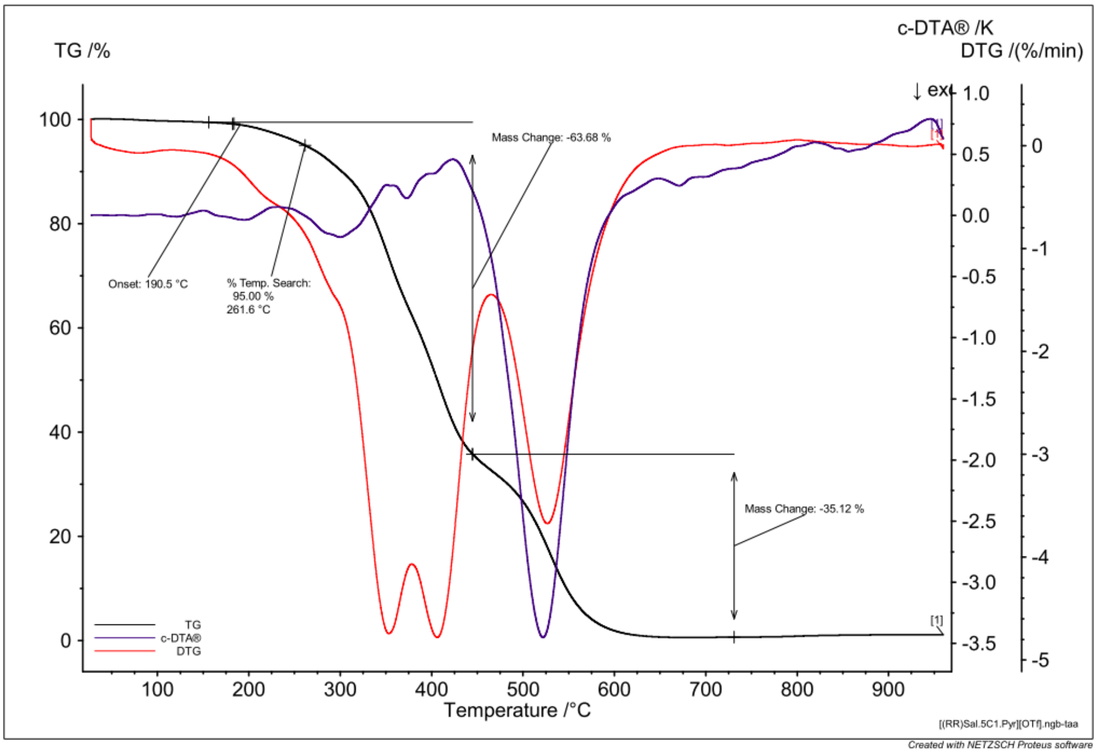

N,N′-bis-[5-((pyridinium)methylene)-salicylidene]-*trans*-(1*R*,2*R*)-cyclohexanediamine di[bis(trifluoromethanesulfonyl)imide], **[(RR)Sal.5C1.Pyr][NTf₂]**

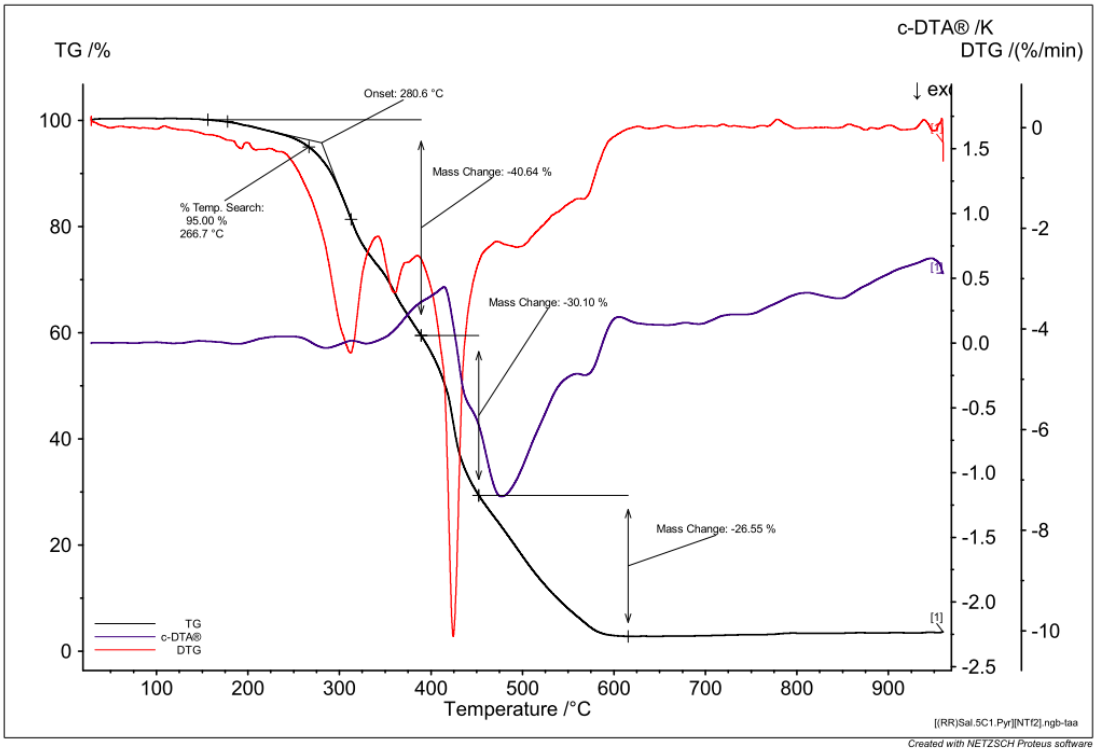

Supplement: Supplementary file 1 [file molecules-30-02173-s001.zip › molecules-3496326-supplementary.pdf]
